# miR-214-5p Regulating Differentiation of Intramuscular Preadipocytes in Goats *via* Targeting *KLF*12

**DOI:** 10.3389/fgene.2021.748629

**Published:** 2021-12-22

**Authors:** Yu Du, Yong Wang, Yanyan Li, Quzhe Emu, Jiangjiang Zhu, Yaqiu Lin

**Affiliations:** ^1^ Key Laboratory of Qinghai-Tibetan Plateau Animal Genetic Resource Reservation and Utilization, Ministry of Education, Southwest Minzu University, Chengdu, China; ^2^ Key Laboratory of Sichuan Province for Qinghai-Tibetan Plateau Animal Genetic Resource Reservation and Exploitation, Southwest Minzu University, Chengdu, China; ^3^ College of Animal Scienceand Veterinary Medicine, Southwest Minzu University, Chengdu, China; ^4^ Animal Breeding and Genetics Key Laboratory of Sichuan Province, Sichuan Animal Science Academy, Chengdu, China

**Keywords:** goat, MIR-214-5p, *KLF*12, intramuscular adipocyte, adipocyte differentiation

## Abstract

Intramuscular fat (i.m.) is an adipose tissue that is deposited between muscle bundles. An important type of post-transcriptional regulatory factor, miRNAs, has been observed as an important regulator that can regulate gene expression and cell differentiation through specific binding with target genes, which is the pivotal way determining intramuscular fat deposition. Thus, this study intends to use RT-PCR, cell culture, liposome transfection, real-time fluorescent quantitative PCR (qPCR), dual luciferase reporter systems, and other biological methods clarifying the possible mechanisms on goat intramuscular preadipocyte differentiation that is regulated by miR-214-5p. Ultimately, our results showed that the expression level of miR-214-5p peaked at 48 h after the goat intramuscular preadipocytes were induced for adipogenesis. Furthermore, after inhibition of the expression of miR-214-5p, the accumulation of lipid droplets and adipocyte differentiation in goat intramuscular adipocytes were promoted by the way of up-regulation of the expression level of lipoprotein lipase (*LPL*) (*p* < 0.05) and peroxisome proliferator-activated receptor gamma (*PPARγ*) (*p* < 0.01) but inhibited the expression of hormone-sensitive lipase (*HSL*) (*p* < 0.01). Subsequently, our study confirmed that Krüppel-like factor 12 (*KLF*12) was the target gene of miR-214-5p. Inhibition of the expression of *KLF*12 promoted adipocyte differentiation and lipid accumulation by upregulation of the expression of *LPL* and CCAAT/enhancer binding protein (*C/EBPα*) (*p* < 0.01). Overall, these results indicated that miR-214-5p and its target gene *KLF*12 were negative regulators in progression of goat preadipocyte differentiation. Our research results provided an experimental basis for finally revealing the mechanism of miR-214-5p in adipocytes.

## Introduction

Intramuscular fat (i.m.) content is an extremely important indicator that affects the tenderness, flavor, and juiciness of goat meat; moreover, the intramuscular fat deposition mainly depends on the differentiation of intramuscular preadipocytes and the accumulation of triglycerides. With the general application of genome sequencing, researchers found that the complexity of biology is the difference in the proportion of non-protein-coding genomes, and most of the long or small non-coding RNAs coordinated protein expression at transcription or translation levels (Taft et al., 2007; Vienberg et al., 2017). Among them, microRNAs (miRNAs) are small non-coding RNAs of approximately 22 nucleotides. Each miRNA can regulate hundreds of target genes’ expression by the way of induced translational inhibition or degradation of transcription products of the target gene via binding to the complementary sites of the 3′-untranslated region (3′UTR) (Hammond, 2015; Colamatteo et al., 2019; Roberts, 2015; Hammond, 2015; Roberts, 2015; Colamatteo et al., 2019). In the process of adipocyte differentiation, miRNAs and its target genes have been extensively studied, that is, miRNAs could target some transcription factors related to adipocyte differentiation (such as PPARs, C/EBPs, KLFs, and SERBPs, etc.) or activate/inhibit certain signaling pathways (such as MAPK, PI3k/Akt, cAMP/PKA/CREB, and Wnt/b-catenin, etc.) to play regulatory roles (Sarjeant and Stephens, 2012; Son et al., 2014; Vienberg et al., 2017). Therefore, the miRNA pathway should be a key mechanism for gene expression. Elucidating the key genes and molecular regulatory networks during differentiation of adipocytes is essential for understanding the physiological process of adipogenesis.

MiR-214-5p is a product of miR-214 in the non-coding RNA transcript dynamin 3 (DNM-3) gene intron on human chromosome 1-NC_000001.10 (Lee et al., 2009; Iizuka et al., 2012). Existing research prompted that miR-214-5p may play an important role in fat formation. For instance, miR-214-5p can promote the adipogenic differentiation of bone marrow stem cells (BMSCs) by regulating TGFβ/Smad2/COL4A1 signaling (Qiu et al., 2018). Using RNA sequencing methods constructing a miRNA-mRNA combinatorial network closely related to the differentiation of chicken abdominal preadipocytes and adipocytes, the research found that miR-214 may play a key role in the differentiation of chicken abdominal adipocytes (Ma et al., 2020). In addition, overexpression of miR199a/214 inhibits brown adipocyte differentiation by directly targeting *PRDM*16 and peroxisome PGC-1α (He et al., 2018). However, the regulatory mechanism of miR-214-5p in goat adipocyte differentiation is still unclear.

Here, we show that miR-214-5p is highly expressed in goat intramuscular adipocytes, and then, we examine the role of regulation and its possible molecular mechanism of miR-214-5p on the differentiation of intramuscular preadipocytes in goats. Our work suggests that miR-214-5p is a negative molecular signal during the goat intramuscular adipocyte differentiation.

## Materials and Methods

### Experimental Animals

The experimental samples came from longissimus dorsi of three healthy 7-day old Jianzhou goats. The experimental animals were anesthetized by intraperitoneal injection of barbiturate at a dose of 100 mg/kg and then bled to death. All experimental procedures involving animals were performed in accordance with the guidelines and regulations approved by the Animal Care and Use Committee of the Southwest Minzu University (Chengdu, Sichuan, China). Detailed procedures for the collection of intramuscular preadipocytes have previously been published (Xu et al., 2018; Xu et al., 2019).

### Cell Culture and Transfection

The 7-day-old goats were euthanized and disinfected for experiment material acquisition using a scalpel to separate the longissimus dorsi muscle and rinsed with sterile PBS. Then, it was digested with type I collagenase (Sigma, United States) for 1 h. The digested mixture was filtered with a 70 µm sieve and centrifuged at 2000 r/min for 5 min. Red blood cell lysate was added for 5 min then centrifuged at 2000 r/min for 5 min. The pellet was washed with PBS, and the goat intramuscular preadipocytes were resuspended in the DMEM/F12 culture medium containing 10% (v/v) fetal bovine serum (FBS, Hyclone, United States). The cells were diluted to 10 ^6^/ml for the subsequent experiment. The F1 of goat intramuscular preadipocytes was cultured in 10% FBS DMEM/F12 culture medium and put in a humidified incubator at 5% CO2 and 37°C. Transient transfections were performed in cell culture plates using Lipofectamine 3000 (Invitrogen, Carlsbad, United States) and the RNAiMAX (Invitrogen, Carlsbad, United States) transfection reagent. Opti-MEM (Gibco BRL Co. United States) was used for dilution. The orginal medium was replaced 6 h after transfection with the adipogenic induction medium, which contained 10% FBS and 50 μmol•L^-1^ oleic acid, to induce preadipocyte differentiation (Shang et al., 2014). The cells were collected after 48 h for RNA extraction.

### Construction of Plasmids and RNA Oligonucleotides

The negative mimics, negative inhibitor, miR-214-5p mimics, and miR-214-5p inhibitor were purchased from GenePharma (GenePharma, Shanghai, China). Coding sequences (CDSs) of goat *KLF*12 were amplified from goat genomic DNA using polymerase chain reaction (PCR), and the *KLF*12 overexpression plasmid was constructed with the pcDNA3.1 vector, *Kpn*I and *Xba*I restriction enzymes (Thermo, MA, United States). The siRNA for *KLF*12 was purchased from Invitrogen (Invitrogen, Shanghai, China). In addition, the binding sites of MT-KLF12 and WT-KLF12 were inserted into the pmirGLO dual luciferase vector (Promega, Madison, United States) using restriction enzymes Xho1 and Xba1(Thermo, MA, United States). The detailed sequences are provided in Table 1.

**TABLE 1:** Sequence of miR-214-5p mimics and inhibitors.

miRNA name	Sequence (5-3′)
Negative mimic	UUC​UCC​GAA​CGU​GUC​ACG​UTT
Negative inhibitor	CAG​UAC​UUU​UGU​GUA​GUA​CAA
miR-214-5p mimic	UGC​CUG​UCU​ACA​CUU​GCU​GUG​C
	ACA​GCA​AGU​GUA​GAC​AGG​CAU​U
miR-214-5p inhibitor	GCA​CAG​CAA​GUG​UAG​ACA​GGC​A
SI-*KLF*12-	UGG​ACA​AGU​CCA​CUG​GCU​CAG​UUU​G
Negative control	F: UUC​UCC​GAA​CGU​GUC​ACG​UTT
	R: ACG​UGA​CAC​GUU​CGG​AGA​ATT
*KLF*12-RT PCR	F: TTA​GCG​CAT​CAT​GTG​ATC​CG
	R: TGG​GGT​GCC​GCT​AAG​AGA​T
OE-*KLF*12	F: GGG​GTA​CCC​CTG​GAT​GAA​TGA​ATA​TCC​ATA​TGA​AG
	R: GCT​CTA​GAG​CCT​TCC​TCA​CTA​TGC​CTA​CCA​GC
*KLF*12-3′Outer	F: CTC​ACC​TGA​AGG​CTC​ATC​GG
	R: TAC​CGT​CGT​TCC​ACT​AGT​GAT​TT
*KLF*12-3′Inner	F: GAG​GCA​TTA​CCG​CAA​ACA​CAC
	R: CGC​GGA​TCC​TCC​ACT​AGT​GAT​TTC​ACT​ATA​GG
*KLF*12-WT	F: CCC​TCG​AGG​AGG​CAT​TAC​CGC​AAA​CAC
	R: GCT​CTA​GAA​AAT​GGC​AGA​GGA​CAC​AGC​AC
*KLF*12*-*MT	F: CCC​TCG​AGG​AGG​CAT​TAC​CGC​AAA​CAC
	MF: CAA​TGC​GGC​GCT​CTT​CAG​CAT​C
	MR: GAT​GCT​GAA​GAG​CGC​CGC​ATT​G
	R: GCT​CTA​GAA​AAT​GGC​AGA​GGA​CAC​AGC​AC

F. sense primer; R. antisense primer.

### Oil Red O and Bodipy Staining

As described in previous investigation with minor modifications (Xu et al., 2019), The cells for morphological observation were cultured in 24-well plates and visualized by Oil red O and Bodipy staining. Before staining, the differentiated adipocytes were fixed with 10% formaldehyde for 30 min and then stained using Oil red O or Bodipy working solution for 15–20 min. After that, the cells were washed three times with PBS and photographed under a microscope.

### Prediction of miR-214-5p Target Genes and the Luciferase Reporter Assay

Target genes of miR-214-5p were predicted using four online databases, which were miRDB (http://mirdb.org/), TargetScan (http://www.targetscan.org/vert_71/), miRT-CDS (http://www.microrna.gr/microT-CDS), and microRNAseq (https://www.encodeproject.org/microrna/microrna-seq/). The miR-214-5p mimic, NC, and KLF12-WT/MT were cotransfected into the goat intromuscular preadipocytes and harvested after adipogenic induction 48 h. Using a Dual-Luciferase Reporter Assay System kit (Promega, Madison, WI, United States), we detected the activity of dual luciferase.

### RNA Extraction and qRT-PCR

Using TRIzol (TaKaRa, Otsu, Japan), total RNA was extracted and stored at −80°C. According to manufacturer instructions, reverse transcription of mRNA was performed using a Revert Aid First Strand cDNA Synthesis Kit (TaKaRa, Otsu, Japan). Using Primer Premier 5, we designed the qRT-PCR primers, which are listed in Table 2. The reaction volume for qRT-PCR was 20 μL and consisted of 1 μL cDNA, 1 μL reverse and forward primers (per gene), 7 μL double-distilled water, and 10 μL TB Green™ Premix Ex Taq™ II (TaKaRa, Otsu, Japan). *U*6 small nucleolar RNA and the ubiquitously expressed transcript (*UXT*) as were used as endogenous controls for miRNA and mRNA, respectively. All reactions were performed three times, and the relative expression levels were determined by the 2^−ΔΔCt^ method.

**TABLE 2:** Sequences of information of primers.

Gene	Reference in GenBank	Primer sequence (5-3′)	Tm (°C)
*PPARγ*	NM_001285658.1	F: AAG​CGT​CAG​GGT​TCC​ACT​ATG	60
		R: GAA​CCT​GAT​GGC​GTT​ATG​AGA​C	
*AP2*	NM_001285623.1	F: TGA​AGT​CAC​TCC​AGA​TGA​CAG​G	58
		R: TGA​CAC​ATT​CCA​GCA​CCA​GC	
*LPL*	NM_001285607.1	F: TCC​TGG​AGT​GAC​GGA​ATC​TGT	60
		R: GAC​AGC​CAG​TCC​ACC​ACG​AT	
*C/EBPβ*	XM_018058020.1	F: CAA​GAA​GAC​GGT​GGA​CAA​GC	60
		R: AACAAGTTCCGCAGGGTG	
*SREBP1*	NM_001285755.1	F: AAG​TGG​TGG​GCC​TCT​CTG​A	58
		R: GCAGGGGTTTCTCGGACT	
*C/EBPα*	XM_018062278.1	F: CCG​TGG​ACA​AGA​ACA​GCA​AC	60
		R: AGG​CGG​TCA​TTG​TCA​CTG​GT	
*FASN*	NM_001285629.1	F: TGTGCAACTGTGCCCTAG	58
		R: GTCCTCTGAGCAGCGTGT	
*HSL*	XM_018062484.1	F: AGG​GTC​ATT​GCC​GAC​TTC​C	60
		R: GTC​TCG​TTG​CGT​TTG​TAG​TGC	
*ACC*	XM_018064169.1	F: GGA​GAC​AAA​CAG​GGA​CCA​TT	
		R: ATCAGGGACTGCCGAAAC	
*UXT*	XP_005700899.1	F: GCA​AGT​GGA​TTT​GGG​CTG​TAA​C	60
		R: TGG​AGT​CCT​TGG​TGA​GGT​TGT	
*U6*	NR_*13*8085.1	F: GGA​ACG​ATA​CAG​AGA​AGA​TTA​GC	64
		R: TGG​AAC​GCT​TCA​CGA​ATT​TGC​G	
*KLF*12	XM_005687692.3	F: TCT​AAG​GTC​ACA​TTT​GGC​AGG​TC	60
		R: CCA​ATC​GGT​GCC​TGT​TGT​CTA​C	
miR-214-5p	MIMAT0036058	UGC​CUG​UCU​ACA​CUU​GCU​GUG​C	62
miR-214-5p RT	—	GTC​GTA​TCC​AGT​GCA​GGG​TCC​GAG​GTA​TTC​GCA​CTG​GAT​ACG​ACG​CAC​AGC​A	—
miR-214-5p qPCR	—	F: GCC​GAG​TGC​CTG​TCT​ACA​CT	58
		R: GTGCAGGGTCCGAGGT	

F. sense primer; R. antisense primer.

### Statistical Analysis

Statistical analyses were performed by SPSS 22 software (SPSS Inc. Chicago, IL, United States), with one-way analysis of variance, and the Tukey method was used to analyze the significance of the difference. qRT-PCR data were analyzed using the 2^−ΔΔCt^ method, GraphPad Prism 5 software was used to plot the data, and the data are expressed as the mean ± SE of ≧ 4 independent experiments, that is, “Mean ± SEM.” All data in the experiment were tested for three times of repeatability. Significant differences between different samples were calculated using the *t*-test in excel. *p* < 0.05 = *; *p* < 0.01 = **.

## Results

### The Expression Pattern of miR-214-5p in Goat Intramuscular Preadipocytes

**FIGURE 1:**
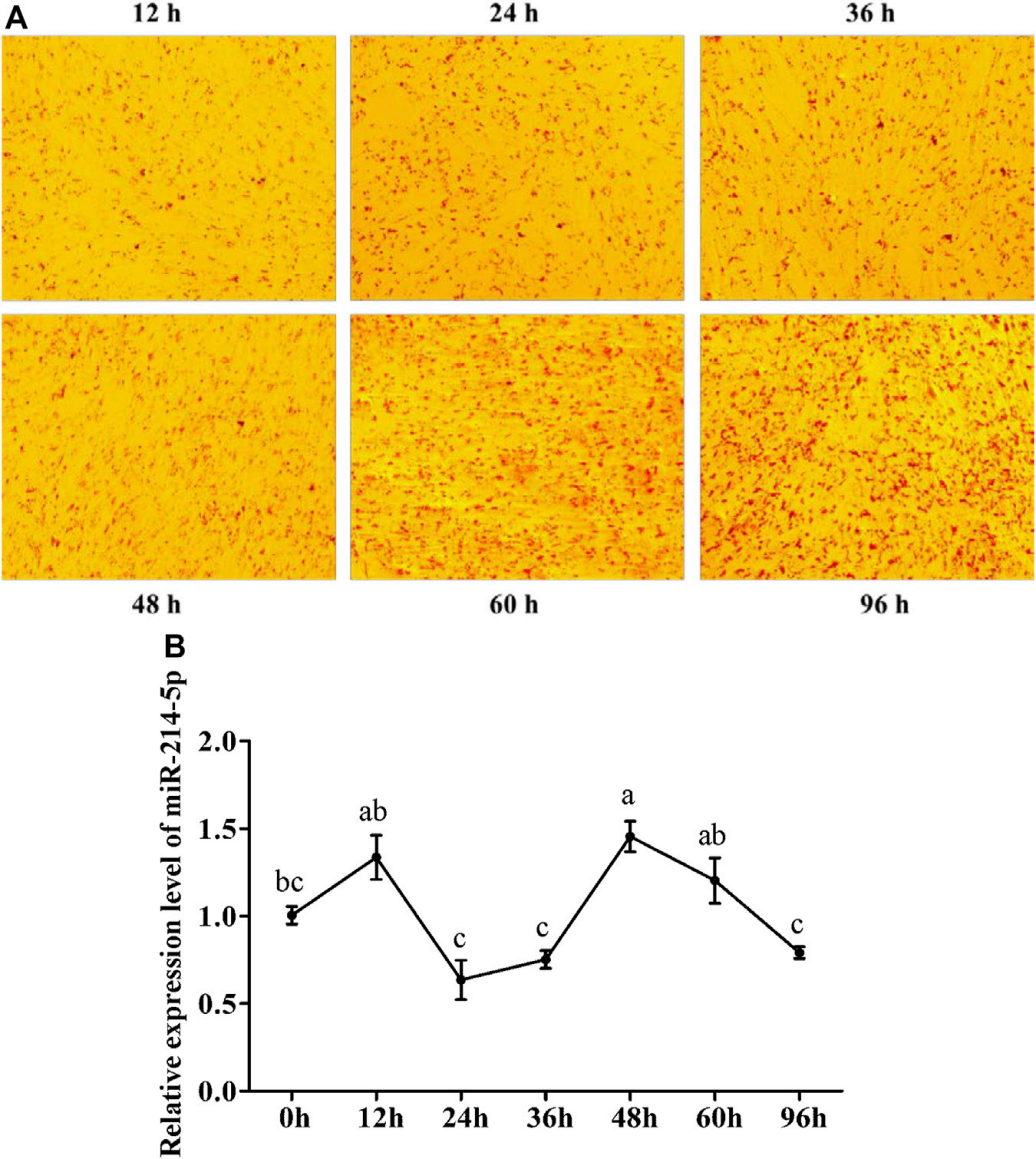
Expression pattern of miR-214-5p. **(A)** Oil Red O staining of goat intramuscular preadipocytes with adipogenic differentiation at different times. **(B)** Expression level of miR-214-5p in different stages of goat intramuscular adipocytes. *p < 0.05; **p < 0.01 vs. NC.

To explore the optimal expression level of miR-214-5p in adipocyte differentiation, we constructed the goat intramuscular adipocyte differentiation model *in vitro* (Figure 1A). The qRT-PCR technique was used for detecting the expression level of miR-214-5p after induced adipogenesis for 0–96 h (Figure 1B), and our results showed that the expression level of miR-214-5p peaked at 48 h, which was significantly higher than that at 0 h (*p* < 0.01).

### Inhibiting miR-214-5p Promoted Goat Preadipocyte Differentiation

**FIGURE 2:**
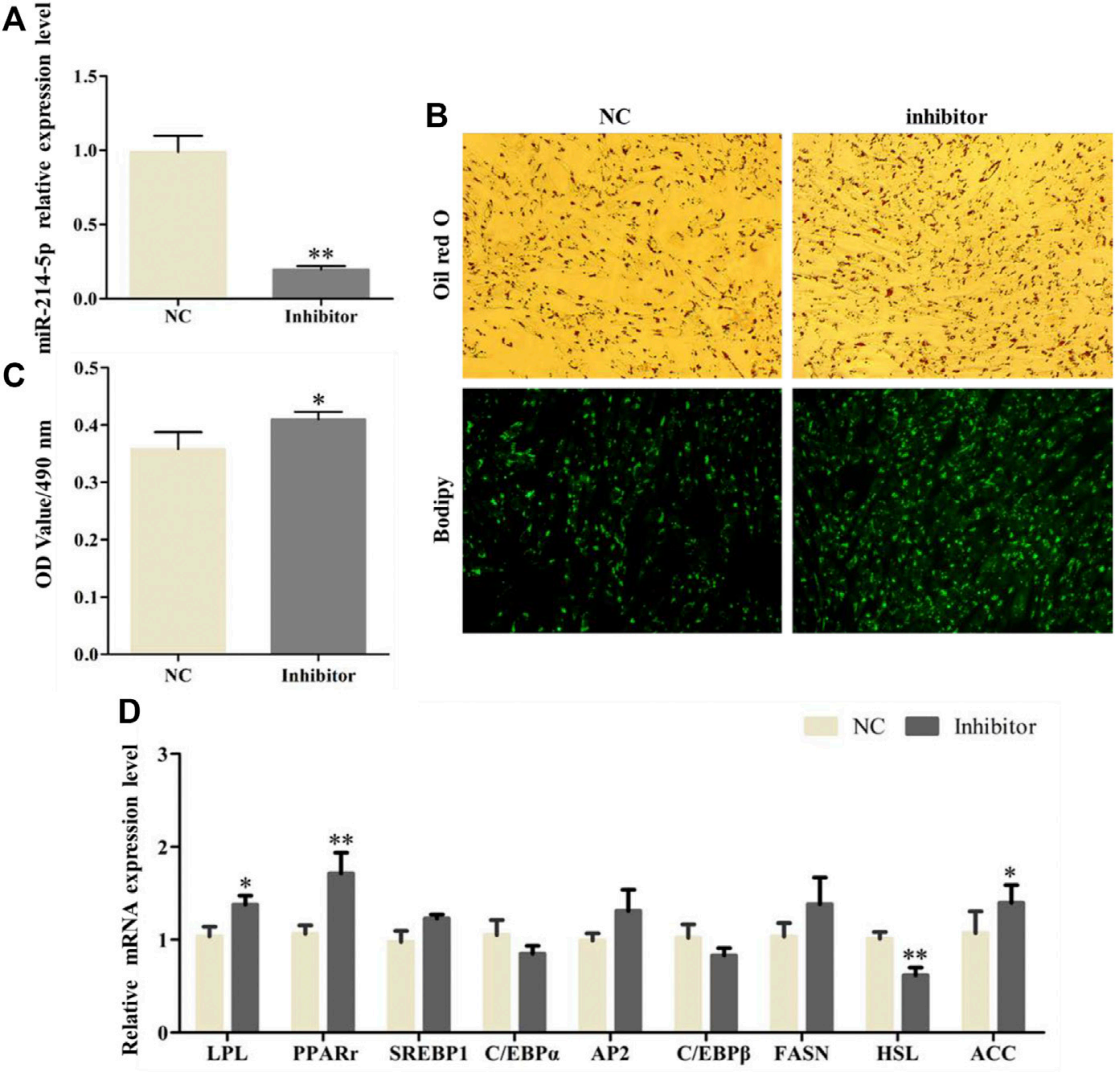
Inhibiting miR-214-5p promoted goat intramuscular adipocyte differentiation. **(A)** Efficiency of the miR-214-5p inhibitor. **(B)** Oil red O staining and Bodipy staining (×200). **(C)** OD value at 490 nm. **(D)** mRNA expression levels of key adipogenic regulatory genes after transfection of the miR-214-5p inhibitor.

After transfection with the miR-214-5p inhibitor in goat intramuscular preadipocytes, the expression of miR-214-5p was lower 79.3% (Figure 2A). In addition, the results of Oil Red O and Bodipy staining showed that inhibiting miR-214-5p could significantly promote the accumulation of lipid droplets in adipocytes (Figure 2B), and the OD value at 490 nm was significantly increased. That is, inhibiting miR-214-5p could elevate triglyceride levels (Figure 2C). To further explore the regulatory role of miR-214-5p, we detected the expression level of key regulatory genes during adipocyte differentiation (Figure 2D). Our results showed that compared with NC, the expression levels of *LPL*, *ACC*, and *PPARγ* were significantly upregulated after inhibiting miR-214-5p, while the expression of *HSL* was significantly downregulated. The above results indicate that inhibiting the expression of miR-214-5p can promote adipocyte differentiation and lipid accumulation by upregulating the expression of *LPL*, *PPARγ*, and *ACC*.

### Overexpression of miR-214-5p Restrained Goat Preadipocyte Differentiation

**FIGURE 3:**
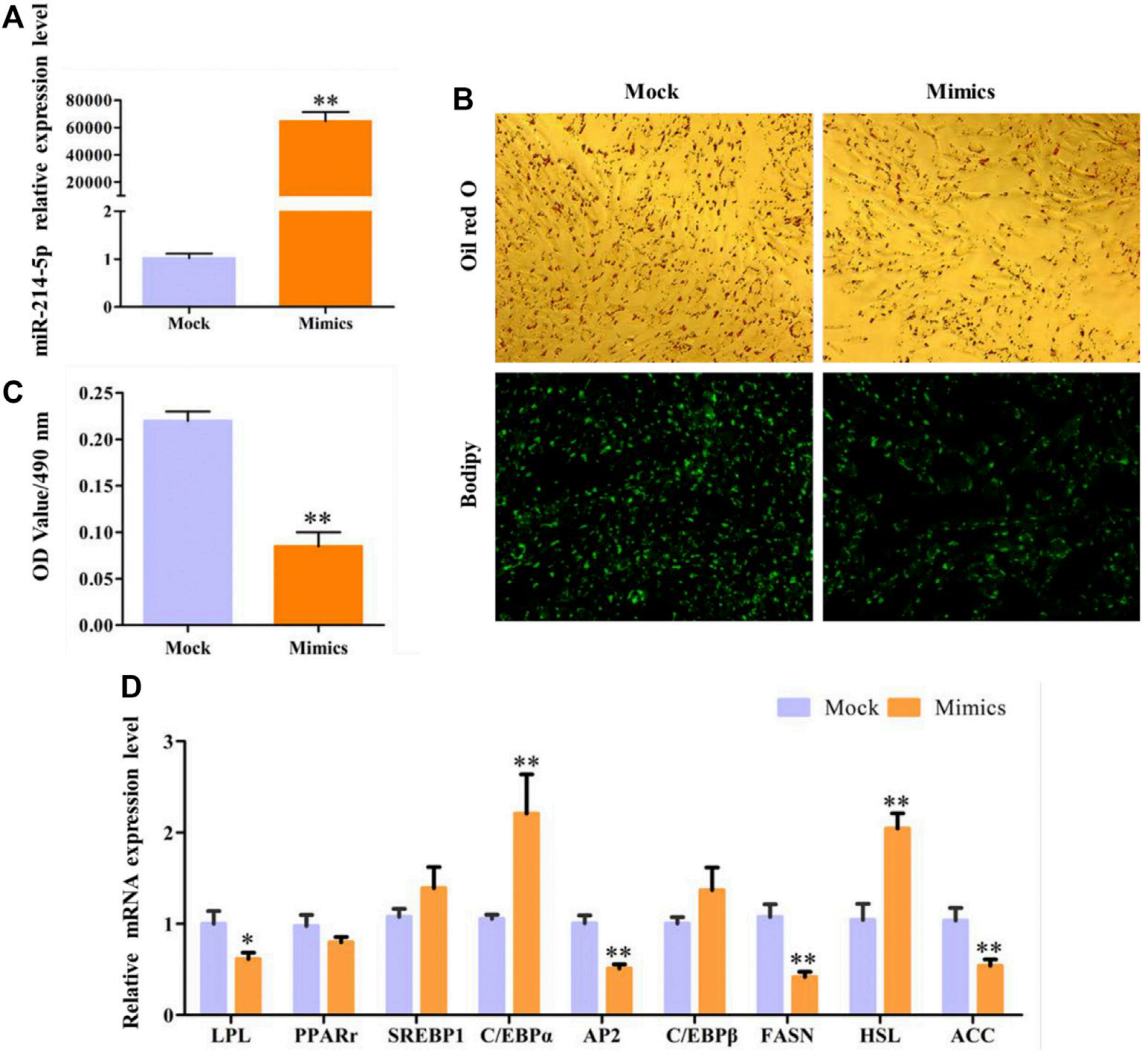
Overexpression of miR-214-5p restrained goat intramuscular adipocyte differentiation. **(A)** Efficiency of miR-214-5p mimics. **(B)** Oil red O staining and Bodipy staining (×200). **(C)** OD value at 490 nm. **(D)** mRNA expression levels of key adipogenic regulatory genes after transfection with miR-214-5p mimics.

In this study, overexpression of miR-214-5p, whose efficiency reached 64635%, can significantly inhibit the accumulation of lipid droplets in preadipocytes in goat muscles, with the OD value at 490 nm (Figures 3A–C). In addition, with the detection of the expression of key regulatory genes during adipocyte differentiation (Figure 3D), we found that overexpression of miR-214-5p can significantly downregulate the expression of *LPL*, *AP*2, *FASN*, and *PPARγ* to promote adipocyte differentiation and lipid droplet accumulation.

### KLF12 as a Target Gene of miR-214-5p

**FIGURE 4:**
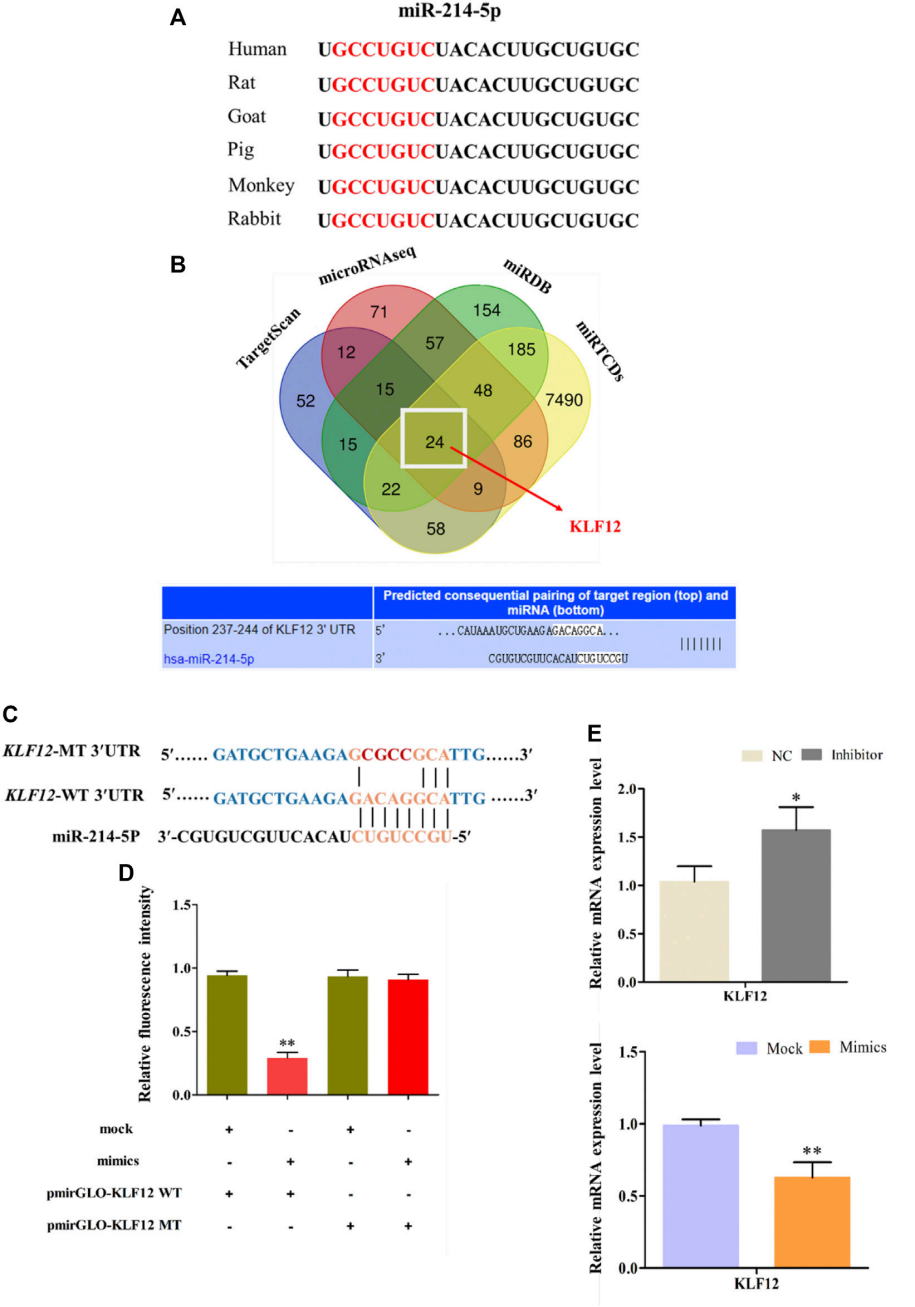
KLF12 as a target gene of miR-214-5p. **(A)** Seed sequences of miR-214-5p. **(B)** Predicting of miR-214-5p target genes. **(C)** Sequence of *KLF*12 3′UTR wide type and mutation type. **(D)** Result of dual luciferase reporter experiment. **(E)** Effect of miR-214-5p on *KLF*12 expression.

Comparing the mature sequence of miR-214-5p among different species, we found that it is highly conserved among mammals (Figure 4A). We used four online pieces of software to predict the common target gene of miR-214-5p (Figure 4B). Then, we selected *KLF*12, which may be related to fat differentiation as the target gene (Shen et al., 2019). Furthermore, in goat intramuscular preadipocytes, dual luciferase report experiment results show that miR-214-5p mimics can significantly inhibit the luciferase activity of Pmir-GLO-*KLF*12 WT. However, it has no effect on Pmir-GLO-*KLF*12 MT (Figures 4C,D). In addition, *KLF*12 mRNA levels in goat intramuscular preadipocytes were significantly upregulated or downregulated after transfection with the miR-214-5p inhibitor or mimics (Figure 4E).

### Interference of KLF12 Promoted Goat Preadipocyte Differentiation

**FIGURE 5:**
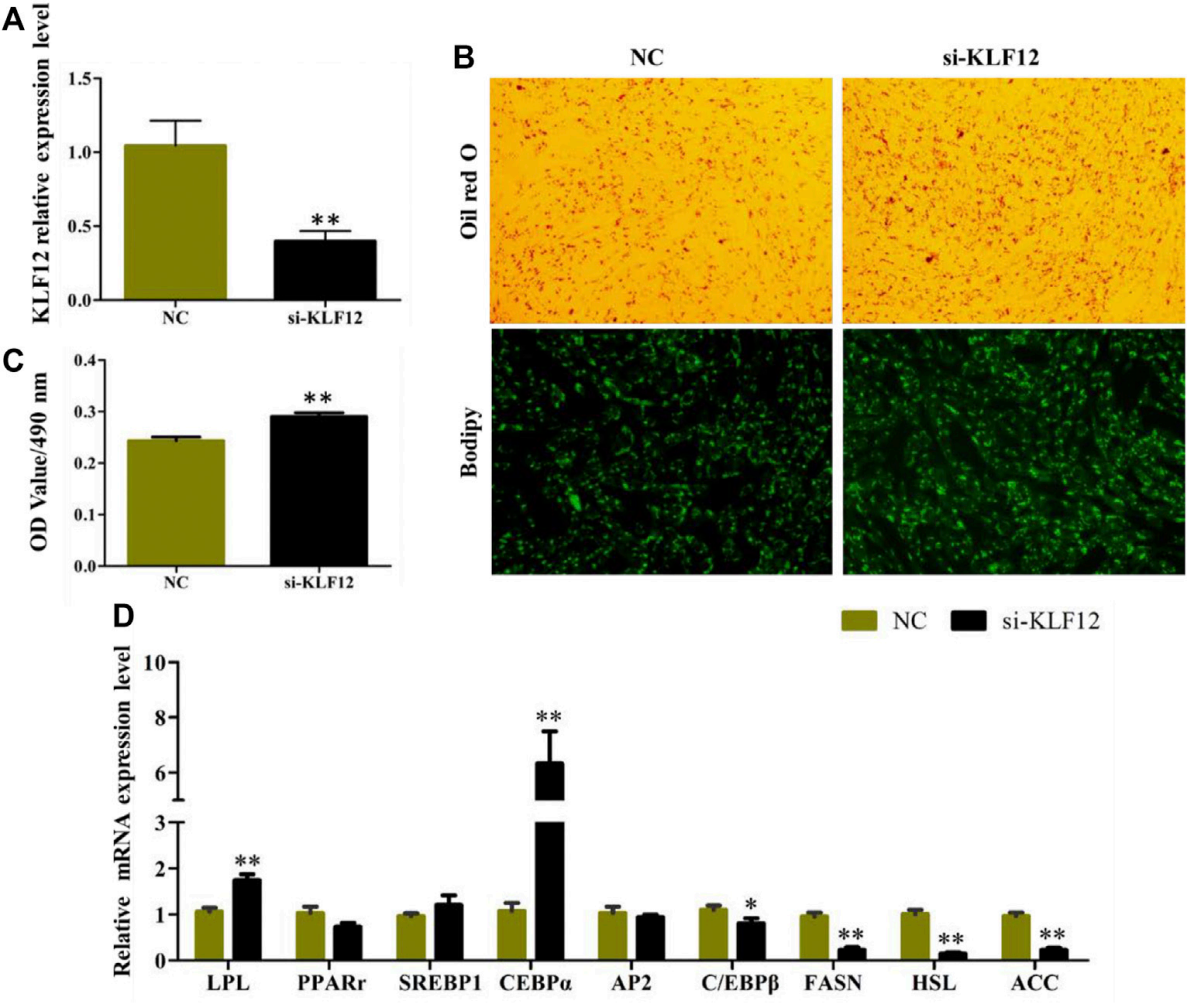
Interference of KLF12 promoted goat intramuscular adipocyte differentiation. **(A)** Efficiency of *KLF*12 siRNA. **(B)** Oil red O staining and Bodipy staining (×200). **(C)** OD value at 490 nm. **(D)** mRNA expression levels of key adipogenic regulatory genes after transfection with si-*KLF*12.

The efficiency of *KLF*12 siRNA in goat intramuscular preadipocytes reached 60.2% (Figure 5A). The results of Oil Red O and Bodipy staining showed that Interference *KLF*12 could promote the accumulation of lipid droplets and the OD value (Figures 5B,C). Moreover, the expression levels of key regulatory genes like *LPL* and *CEBPα* (*p* < 0.01) were significantly upregulated after transfection *KLF*12 siRNA (Figure 5D). The above results indicated that inhibiting the expression of *KLF*12 promoted adipocyte differentiation and lipid accumulation by upregulating the expression of *LPL* and *CEBPα*.

### Overexpression of KLF12 Inhibited Goat Preadipocyte Differentiation

**FIGURE 6:**
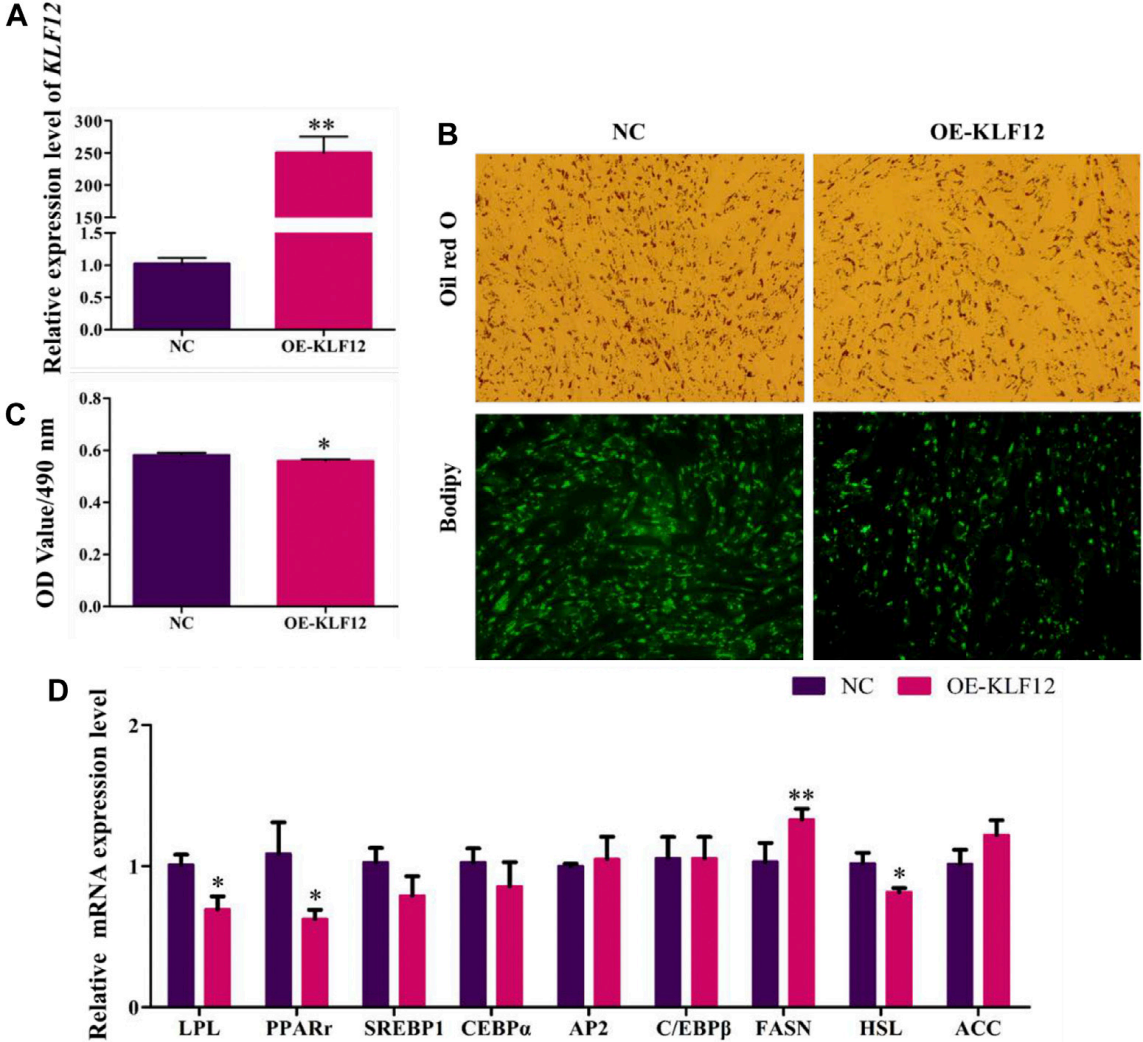
Overexpression of KLF12 inhibited goat preadipocyte differentiation. **(A)** Efficiency of OE-*KLF*12. **(B)** Oil red O staining and Bodipy staining (×200). **(C)** OD value at 490 nm. **(D)** mRNA expression levels of key adipogenic regulatory genes after overexpression of KLF12.

For a further study, *KLF*12 was overexpressed in goat intramuscular preadipocytes, which upregulated to 249% (Figure 6A). According to our results, overexpression of *KLF*12 can significantly inhibit the accumulation of lipid droplets in preadipocytes in goat muscles, with the OD value at 490 nm (Figures 6B,C). Moreover, we found that this effect was achieved by inhibiting the expression level of *LPL*, *PPARγ*, and *HSL* (*p* < 0.05) (Figure 6D).

### KLF12 is a Functional Target of miR-214-5p

**FIGURE 7:**
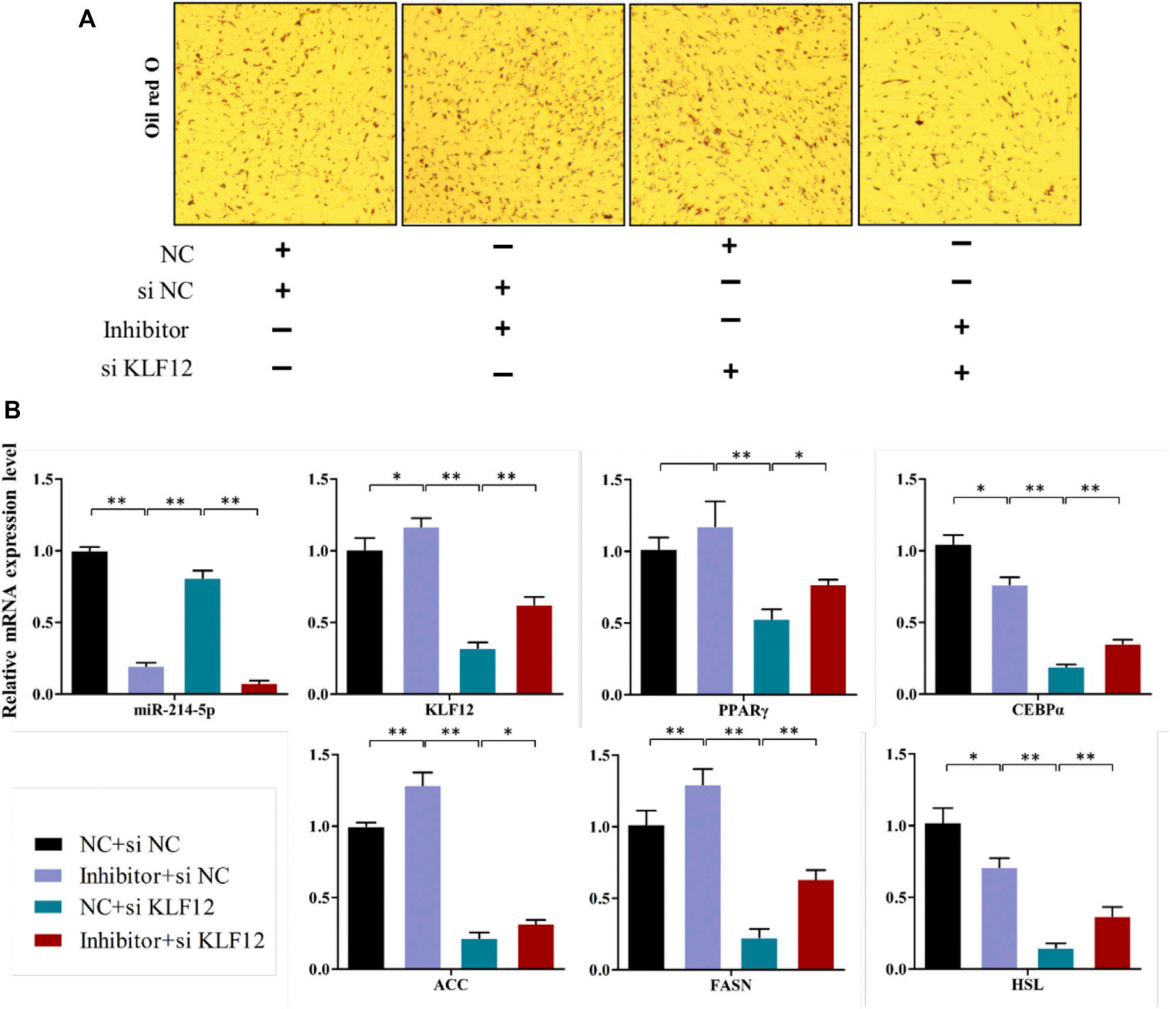
KLF12 is a functional target of miR-214-5p. **(A)** Oil red O staining (×200). **(B)** mRNA expression levels of key adipogenic regulatory genes.

The above studies indicate that *KLF*12 is a potential target of miR-214-5p, and it can inhibit the differentiation of goat intramuscular preadipocytes. Therefore, we verified whether *KLF*12 can counteract the repression effect of miR-214-5p on adipogenesis. Our results showed that inhibiting miR-214-5p could upregulate the expression of *KLF*12 in goat intramuscular preadipocytes. According to this phenomenon, we co-transfected the miR-214-5p inhibitor and si-*KLF*12 into goat intramuscular preadipocytes. Then, we found that inhibiting the expression of *KLF*12 can partially restore the lipid droplet accumulation and key adipogenicity gene expression upregulated, such as *PPARγ*, *CEBPα*, *ACC*, *FASN*, and *HSL*, which were caused by inhibiting the expression of miR-214-5p (Figures 7A,B). Overall, *KLF*12 is the functional target of miR-214-5p and can participate in the adipogenesis regulated by miR-214-5p.

## Discussion

MiRNAs are important gene expression. In animals, miRNAs can control each biological process via combining with the complementary sequence of the 3′ untranslated region (3′ UTR) on the target messenger RNA transcript (mRNA) and resulting in translation inhibition or gene silencing (Hafner et al., 2010; Zhou et al., 2013; Dai and Zhou, 2010; Dai and Zhou, 2010; Hafner et al., 2010; Zhou et al., 2013). Far more than that, one miRNA can target hundreds of mRNAs at the same time, and a 3′ UTR region of one target gene can also have multiple miRNA combination sites (Tili et al., 2007; Hou et al., 2009). Thus, identification the miRNA-mRNA regulatory network is essential for an in-depth understanding of the cell development and maintenance of cell homeostasis.

In this study, we first explored the role of miR-214-5p in differentiation of goat intramuscular preadipocytes. Through morphological observation, we found that inhibiting the expression of miR-214-5p promoted the accumulation of lipid droplets in adipocytes, while overexpression got the opposite result. Further exploring its molecular mechanism, we found that inhibiting the expression of miR-214-5p promoted the differentiation of goat intramuscular adipocytes by upregulating the expression levels of *LPL* and *PPARγ*, while downregulating the expression level of *HSL*. Otherwise, overexpression of miR-214-5p inhibited the differentiation of goat intramuscular adipocytes by inhibiting the expression of *LPL*, *AP*2, *FASN*, and *ACC*, while upregulating the expression levels of *C/EBPα* and *HSL*. Among them, *LPL* is mainly a triglyceride lipase secreted by fat cells, skeletal muscle cells, and cardiomyocytes. Studies have shown that inhibiting the expression of *LPL* in 3T3-L1 adipocytes during fat deposition can inhibit lipid accumulation (Kim et al., 2019; Nimonkar et al., 2020). *PPARγ* is the main regulator of adipogenesis, which can extensively control adipogenesis in adipocyte progenitor cells *in vitro* and *in vivo*, and the epigenomic activation of *PPARγ* can stimulate adipogenesis by inducing terminal differentiation of targeted preadipocytes (Cristancho and Lazar, 2011). *C/EBPα* plays an important role in promoting the early differentiation of preadipocytes and the terminal differentiation of adipocytes (Uysal et al., 2000; Furuhashi et al., 2007). A previous study showed that *C/EBPα* and *PPARγ* usually coordinate and maintain the expression of adipocyte genes in a synergistic manner during adipogenesis. Moreover, the ectopic expression of any of the transcription factors of *C/EBPα* or *PPARγ* will lead to the expression of the other (Farmer, 2006; Tontonoz and Spiegelman, 2008). Therefore, it plays an important regulatory mechanism in the synthesis and transportation of substances, the secretion of adipocyte-specific proteins, and various metabolic programs related to cell differentiation (Moseti et al., 2016). Adipocytes are the main expression place of adipocyte fatty acid-binding protein (*AP*2), and knockout of *AP*2 will significantly inhibit fatty acid transport to regulate lipid transport (Uysal et al., 2000; Furuhashi et al., 2007). Fatty acid synthase (*FASN*) is a key enzyme for fatty acid *de novo* synthesis, and inhibition of *FASN* can induce a rapid decrease in fat storage in mice (Schleinitz et al., 2010). *HSL* is an intracellular neutral lipase that catalyzes the rate-limiting step in adipose tissue lipolysis, and its activity is under acute hormonal and neuronal control, playing an important role in the differentiation of preadipocytes and lipid droplet accumulation (Casimir and Ntambi, 1996; Kong et al., 2017). Overexpression of *HSL* can downregulate the expression of key adipogenic genes such as *FASN*, *LPL*, and *ACOT*12 in the subcutaneous and visceral adipocytes to regulate fat deposition (Fang et al., 2017). Acetyl-CoA carboxylase (*ACC*) catalyzes the rate-limiting step of *de novo* fat formation, and *ACC* inactivation can reduce liver fat content in patients with non-alcoholic steatohepatitis (Bates et al., 2020). Based on the regulating roles of simulation or inhibition of miR-214-5p on adipogenic differentiation marker genes, we proved that miR-214-5p acted as a negative regulator in goat intramuscular adipocytes.

Subsequently, we used online computing software which predicted the possible target genes of miR-214-5p and proved that *KLF*12 was one of the targets of miR-214-5p. In addition, our results showed that mimicking and inhibiting the expression of miR-214-5p significantly down- or upregulated the expression of *KLF*12 mRNA; that is, miR-214-5p was a negative regulator of its target gene. *KLF*12 is a member of the *KLF* family. A large number of studies have confirmed that KLFs play important roles in regulating the differentiation of adipocytes. For example, clusterin CLU regulates adipocyte differentiation by reducing the ubiquitination of *KLF*5 (Oh et al., 2020). Overexpression of *KLF*7 promotes the proliferation of chicken abdominal preadipocytes and inhibits differentiation (Zhang et al., 2013). Furthermore, *C/EBPβ* combined with the *KLF*10 promoter to transactivate *KLF*10 expression, and overexpression of *KLF*10 in 3T3-L1 preadipocytes could inhibit adipogenesis and reduce the expression of *C/EBPα* and *PPARγ* (Liu et al., 2018). Here, we synthesized exogenous siRNA against *KLF*12 and constructed a *KLF*12 eukaryotic expression vector. After transfection into goat intramuscular preadipocytes, our results showed that interference of *KLF*12 promoted the differentiation of goat intramuscular adipocytes by significantly upregulating the expression levels of *LPL* and *C/EBPα*. Overexpression of *KLF*12 inhibits adipocyte differentiation by inhibiting the expression of *LPL*, *PPARγ*, and *HSL*. The above study proved that *KLF*12 was a negative regulator during goat intramuscular preadipocyte differentiation.

The regulatory effects of miR-214-5p and *KLF*12 on adipogenesis were demonstrated, and whether *KLF*12 was a functional target of miR-214-5p is still unknown. Therefore, a rescue experiment was performed and designed to verify whether *KLF*12 can counteract the repression effect of miR-214-5p on adipogenesis. Our results revealed that *KLF*12 could partially restore the lipid droplet accumulation and key adipogenicity gene expressions like *PPARγ*, *CEBPα*, *ACC*, *FASN*, and *HSL*. Taken together, our results indicated that *KLF*12 was a functional target of miR-214-5p and can participate in the adipogenesis that is regulated by miR-214-5p.

## Conclusion

In conclusion, our results support the concept that miR-214-5p acts to downregulate adipogenesis and *KLF*12 upregulates adipogenesis. This moderating effect of miR-214-5p was accomplished by regulating the expression level of key adipogenic genes and inhibiting the expression level of its target gene *KLF*12. Our results improved the target regulation network of miR-214-5p and provided insight into the potential value for a further study of the molecular mechanisms related to miR-214-5p and *KLF*12 regulating adipocyte differentiation and the lipid metabolism.

## Data Availability

The datasets presented in this study can be found in online repositories. The names of the repository/repositories and accession number(s) can be found in the article/Supplementary Material.
