# Supplementary material for: miR-214-5p Regulating Differentiation of Intramuscular Preadipocytes in Goats via Targeting KLF12
Source: Front Genet. 2021 Dec 22;12:748629. doi: 10.3389/fgene.2021.748629 (PMC8730364; doi:10.3389/fgene.2021.748629)
Supplement: Supplementary file 4 [file DataSheet1.PDF]

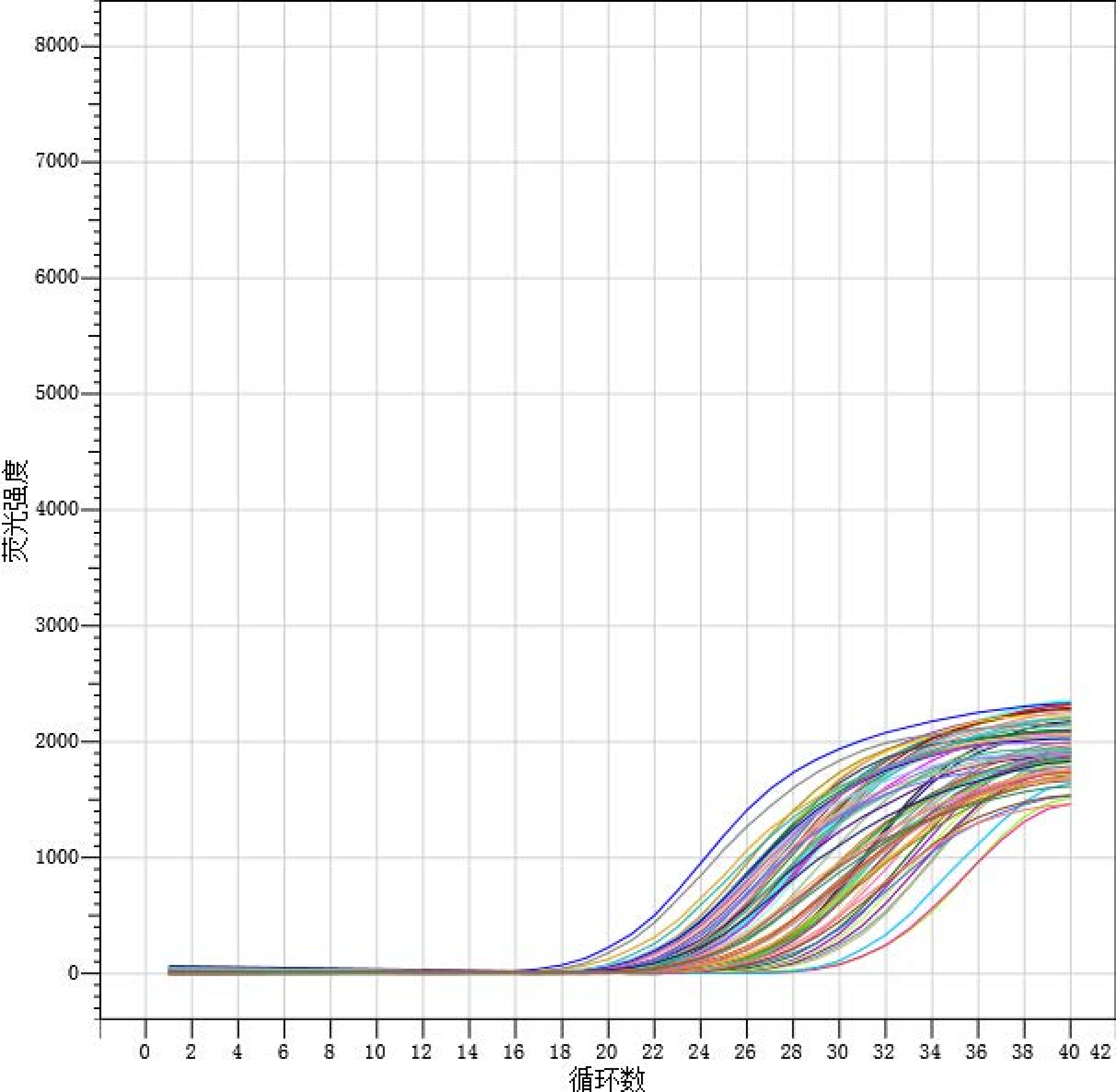

8000
7000
6000
5000
4000
3000
2000
1000
0
荧光强度
0 2 4 6 8 10 12 14 16 18 20 22 24 26 28 30 32 34 36 38 40 42
循环数

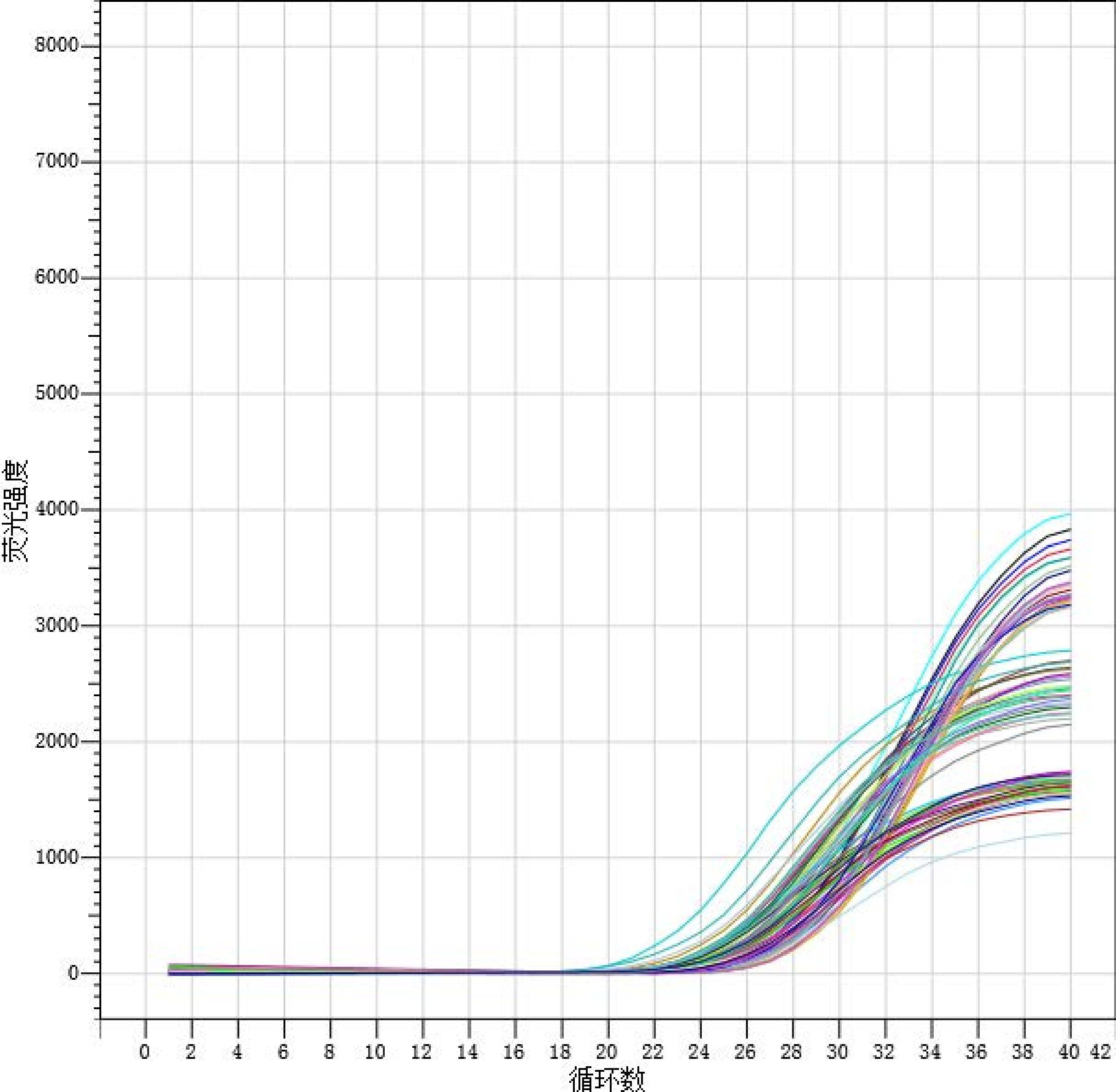

8000
7000
6000
5000
4000
3000
2000
1000
0
荧光强度
0
2
4
6
8
10
12
14
16
18
20
22
24
26
28
30
32
34
36
38
40
42
循环数

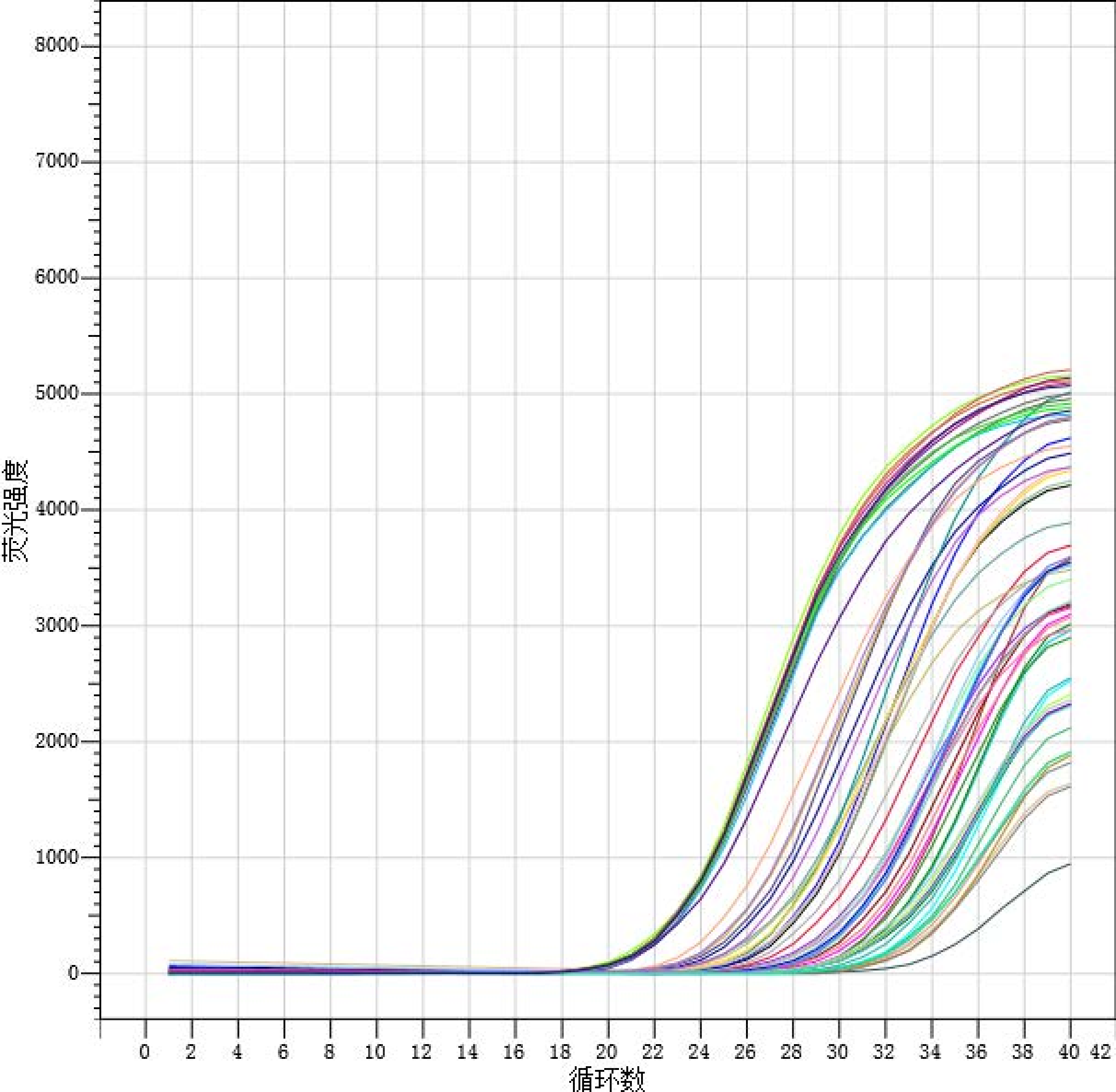

8000
7000
6000
5000
4000
3000
2000
1000
0
荧光强度
0 2 4 6 8 10 12 14 16 18 20 22 24 26 28 30 32 34 36 38 40 42
循环数

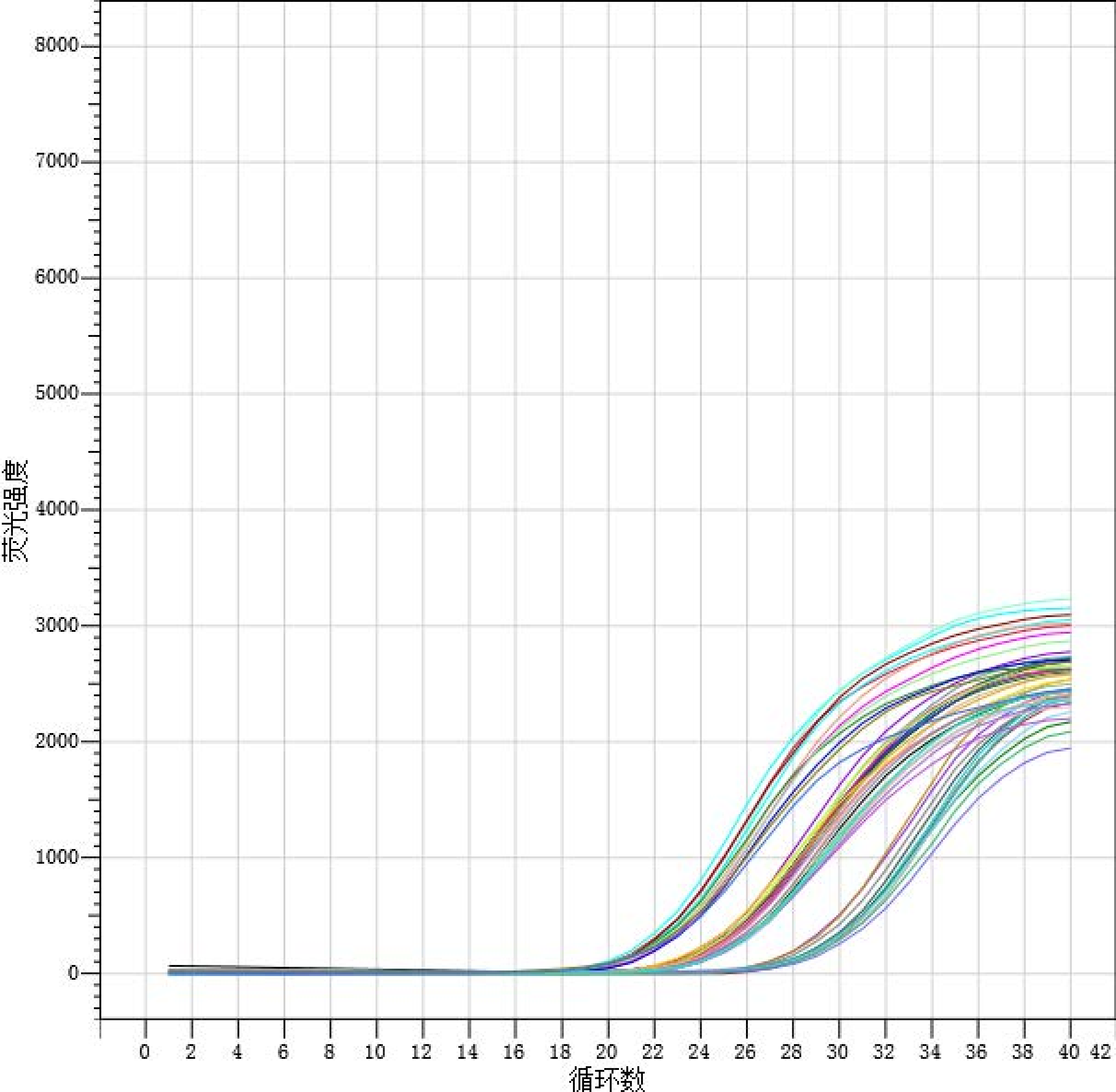

8000
7000
6000
5000
4000
3000
2000
1000
0
荧光强度
0 2 4 6 8 10 12 14 16 18 20 22 24 26 28 30 32 34 36 38 40 42
循环数

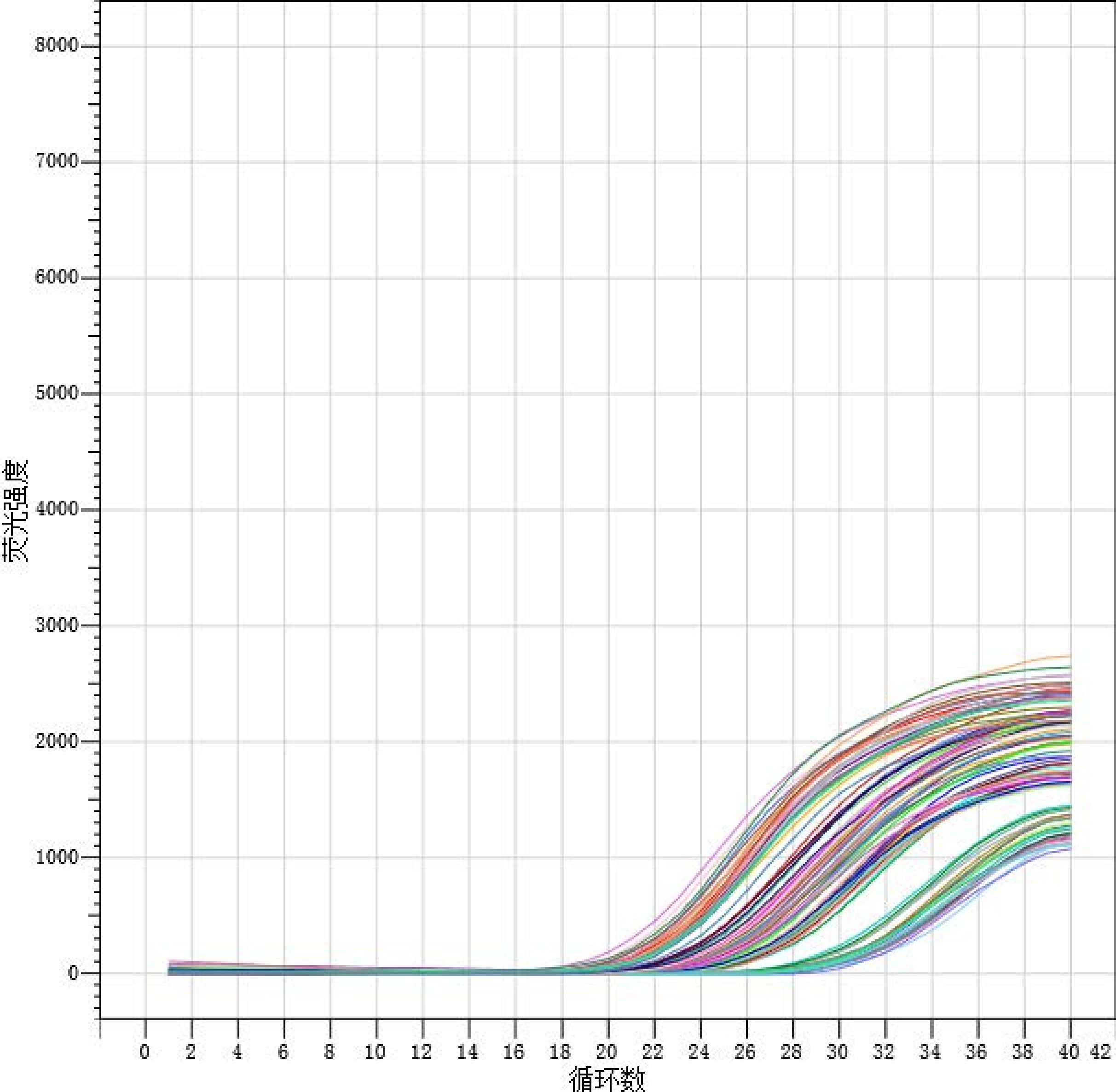

8000
7000
6000
5000
4000
3000
2000
1000
0
荧光强度
0 2 4 6 8 10 12 14 16 18 20 22 24 26 28 30 32 34 36 38 40 42
循环数

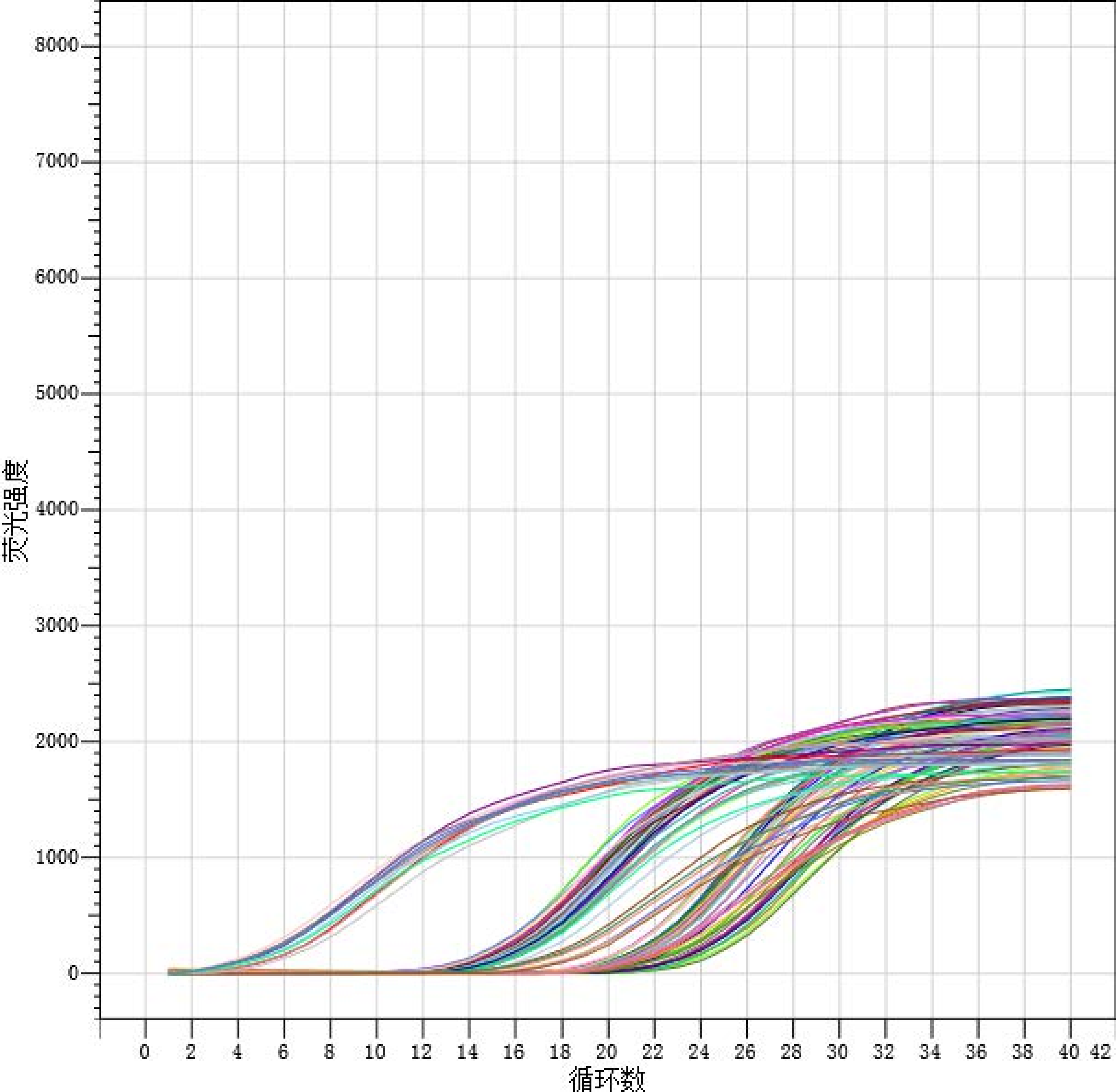

8000
7000
6000
5000
4000
3000
2000
1000
0
荧光强度
0 2 4 6 8 10 12 14 16 18 20 22 24 26 28 30 32 34 36 38 40 42
循环数

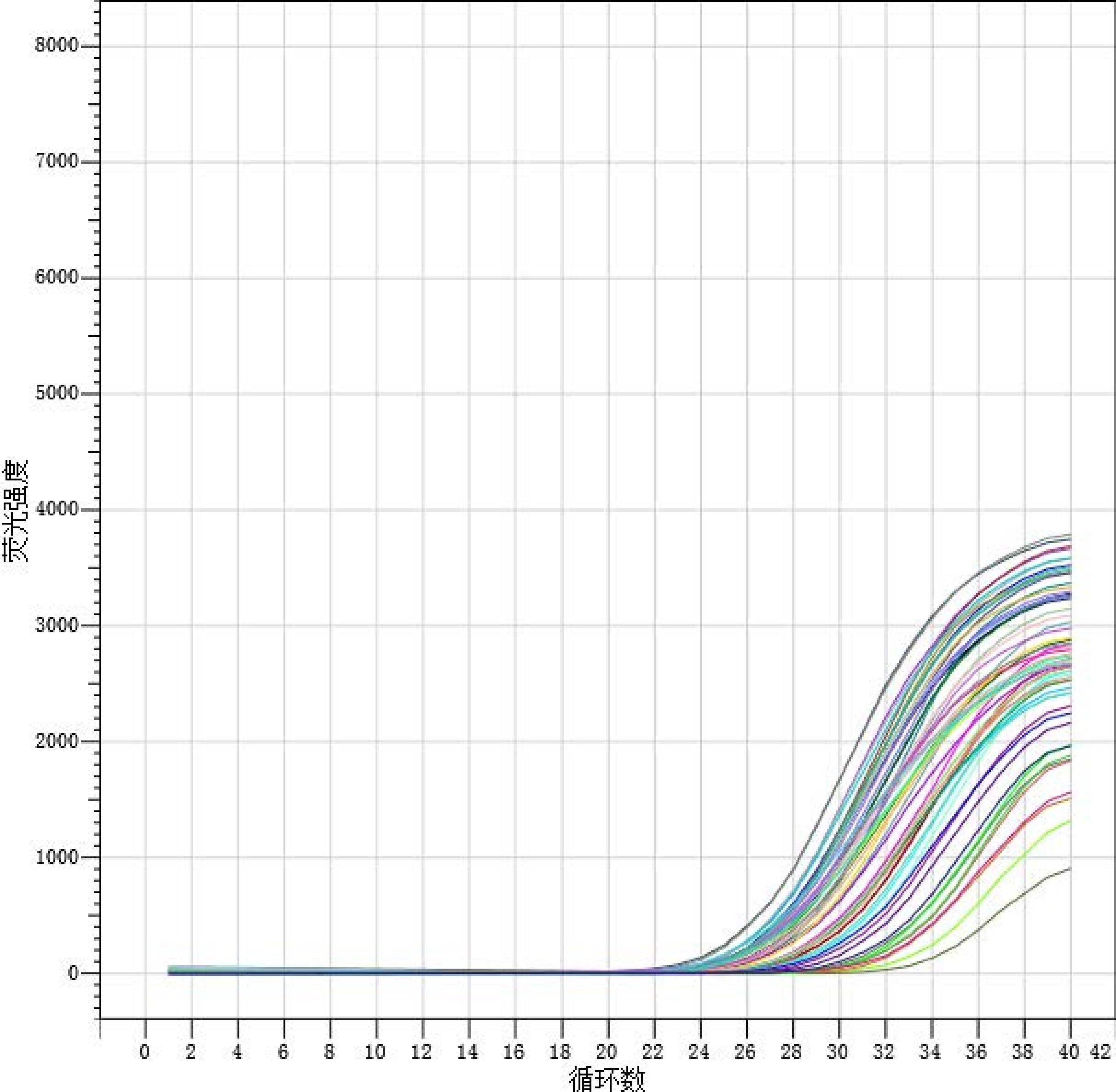

8000
7000
6000
5000
4000
3000
2000
1000
0
荧光强度
0 2 4 6 8 10 12 14 16 18 20 22 24 26 28 30 32 34 36 38 40 42
循环数

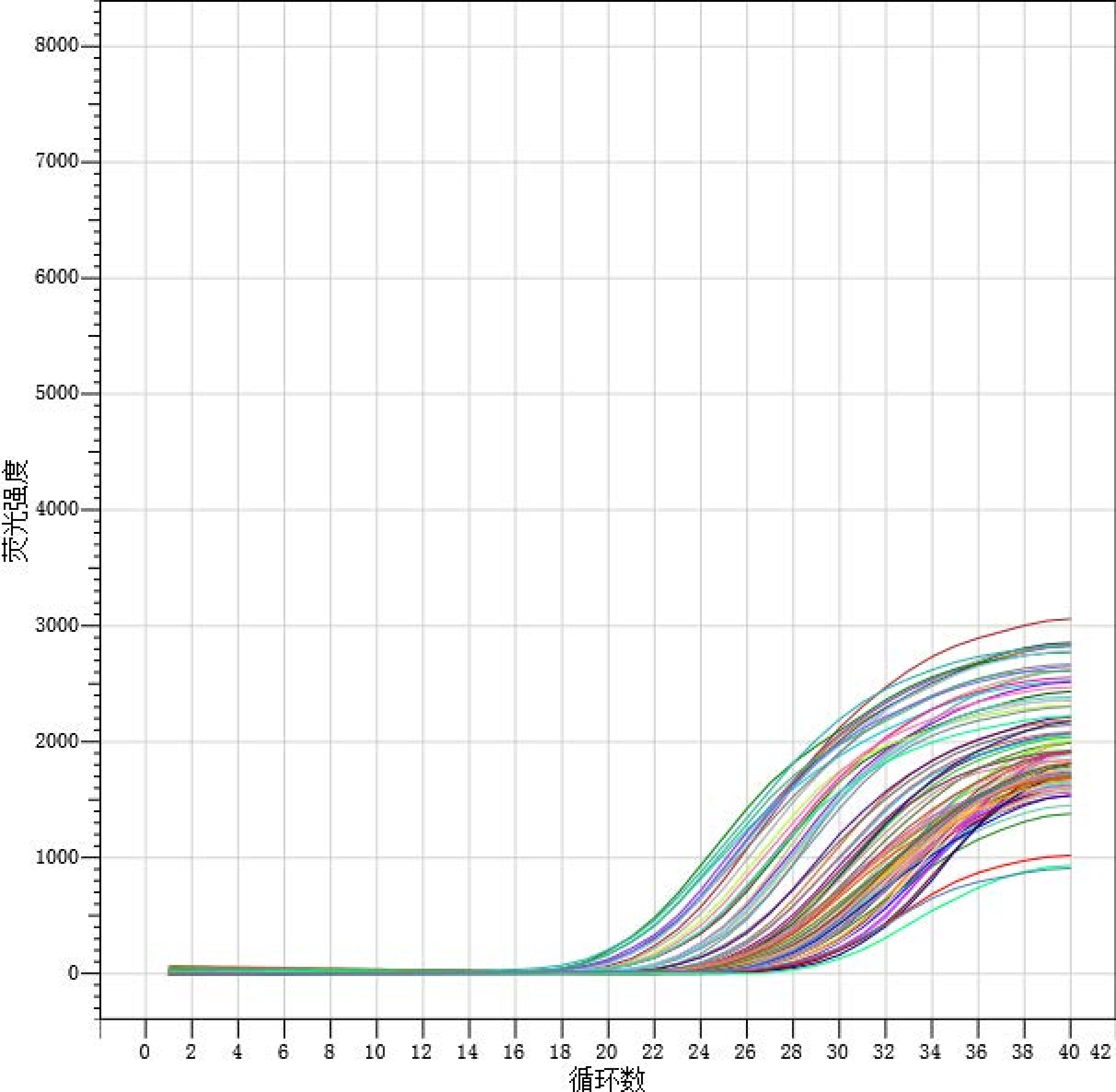

8000
7000
6000
5000
4000
3000
2000
1000
0
荧光强度
0
2
4
6
8
10
12
14
16
18
20
22
24
26
28
30
32
34
36
38
40
42
循环数

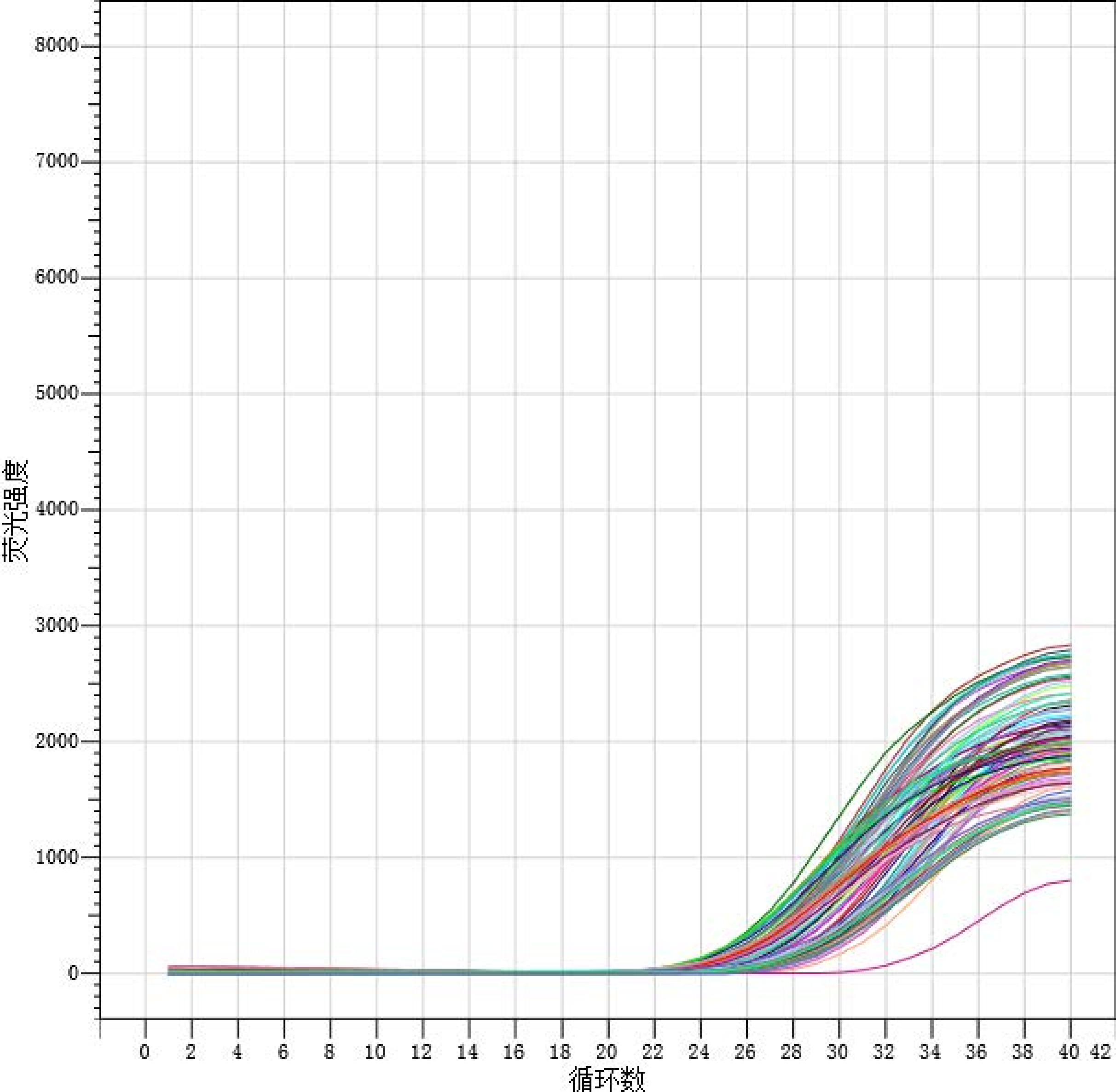

荧光强度
8000
7000
6000
5000
4000
3000
2000
1000
0
0 2 4 6 8 10 12 14 16 18 20 22 24 26 28 30 32 34 36 38 40 42
循环数

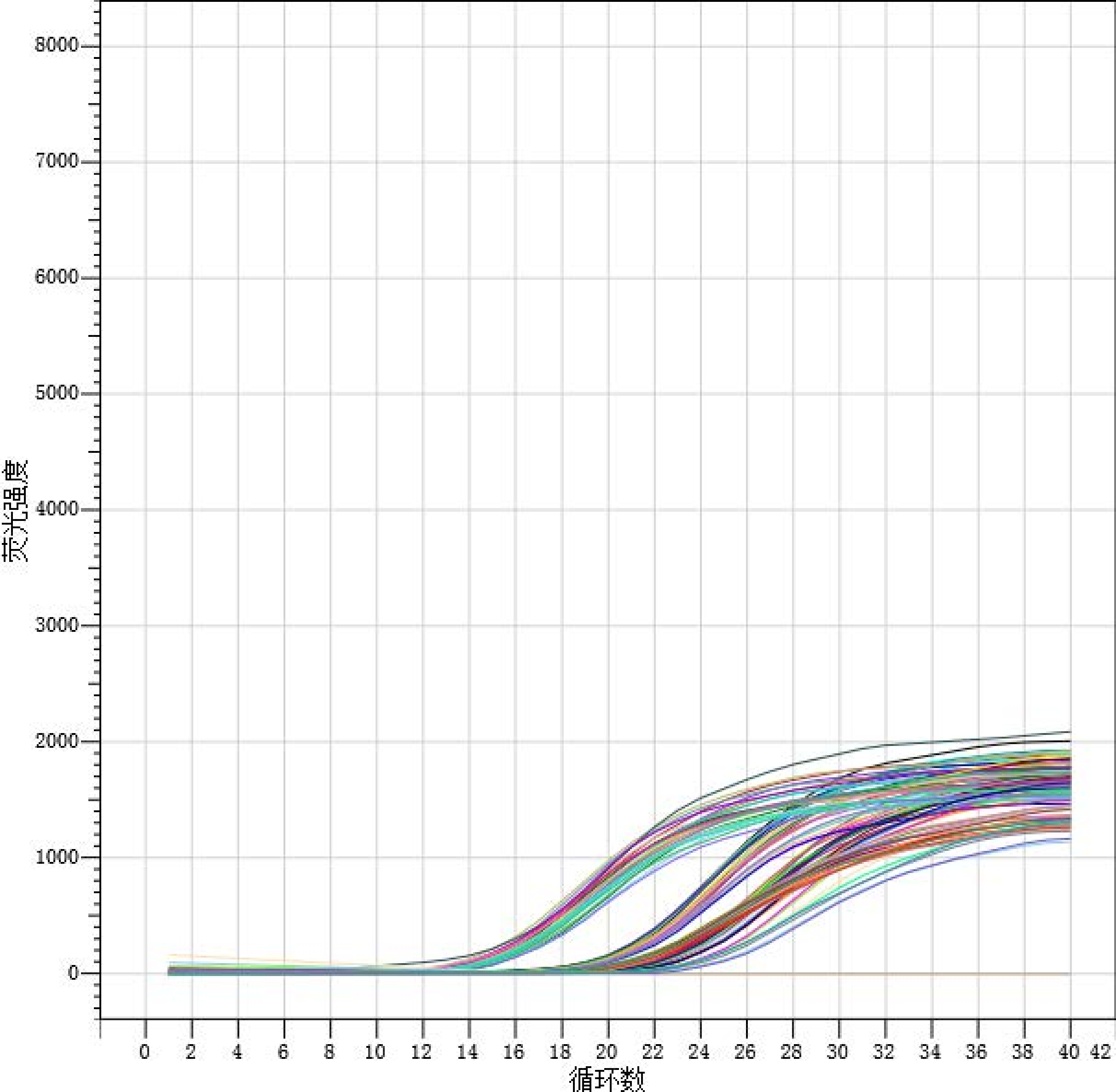

8000
7000
6000
5000
4000
3000
2000
1000
0
荧光强度
0 2 4 6 8 10 12 14 16 18 20 22 24 26 28 30 32 34 36 38 40 42
循环数

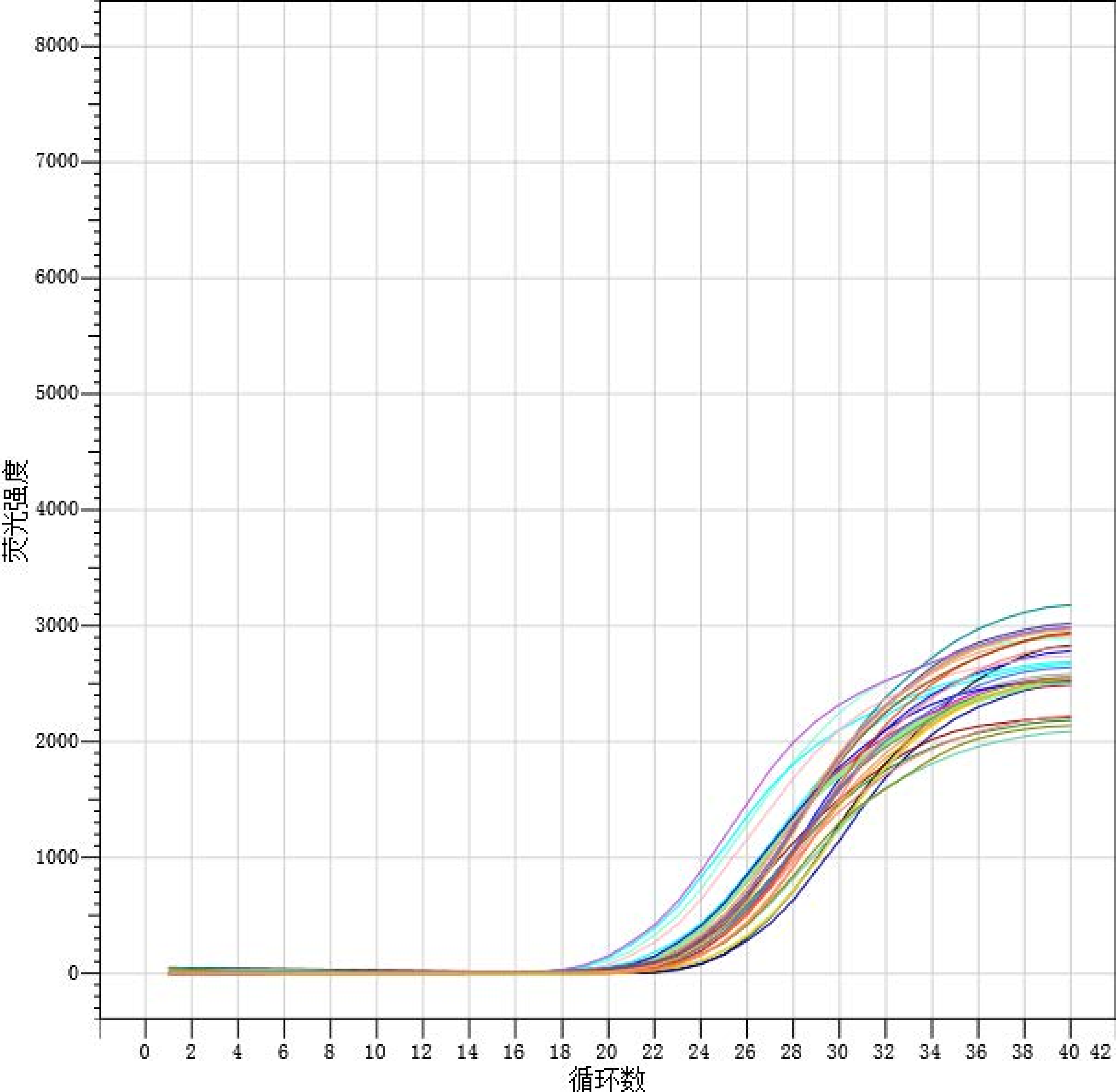
荧光强度
8000
7000
6000
5000
4000
3000
2000
1000
0
0 2 4 6 8 10 12 14 16 18 20 22 24 26 28 30 32 34 36 38 40 42
循环数

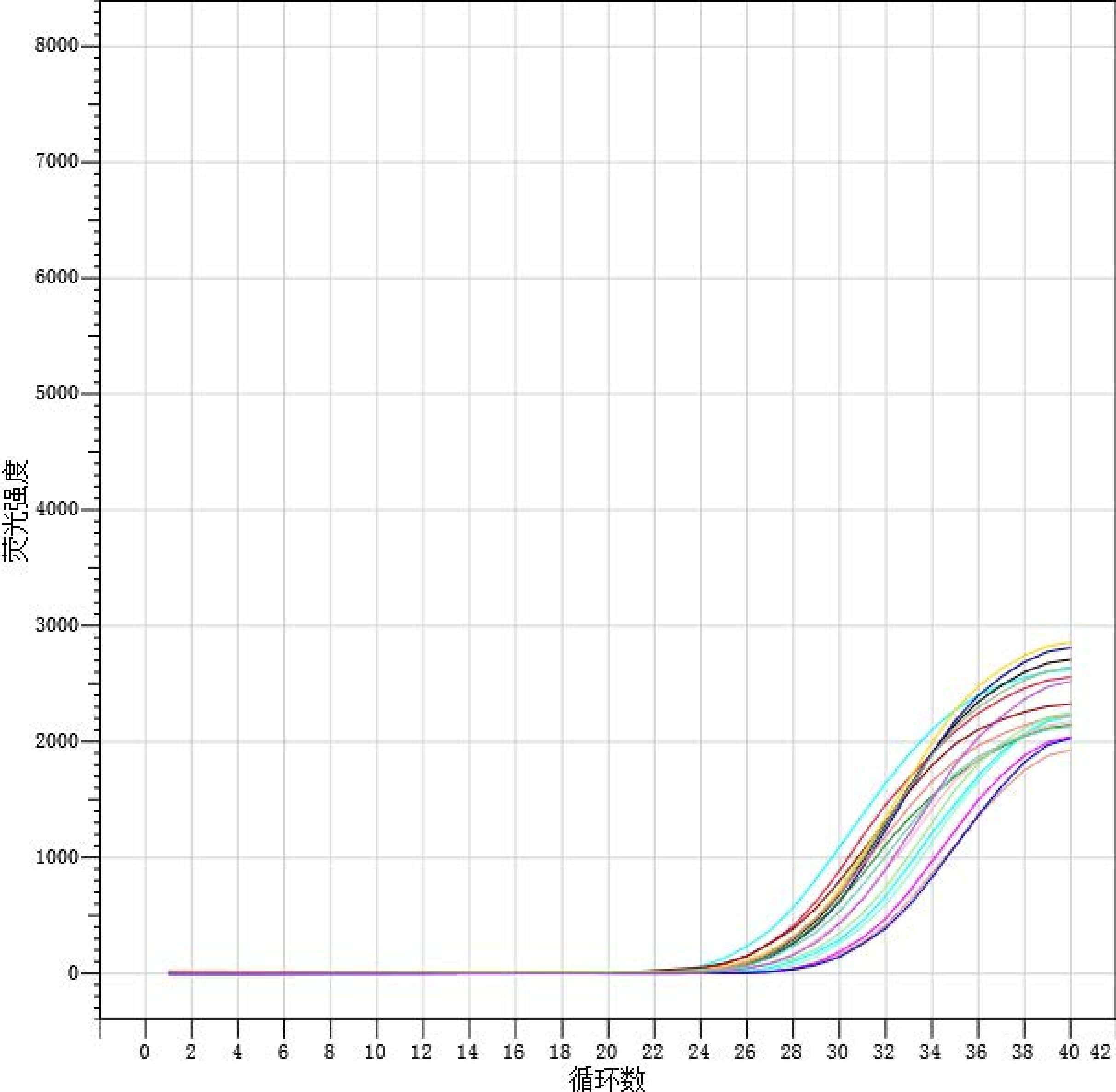

8000
7000
6000
5000
4000
3000
2000
1000
0
荧光强度
0
2
4
6
8
10
12
14
16
18
20
22
24
26
28
30
32
34
36
38
40
42
循环数

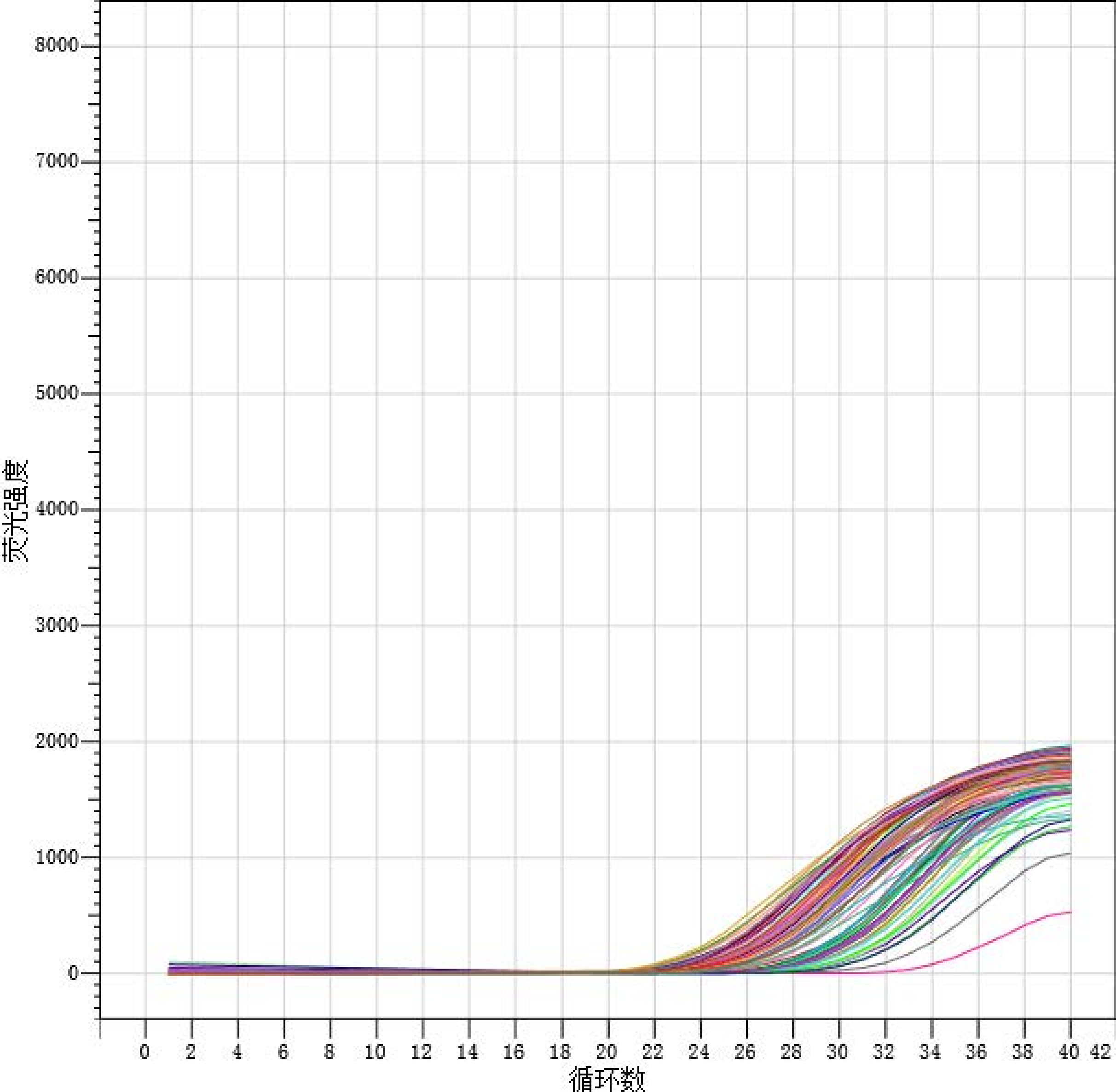

8000
7000
6000
5000
4000
3000
2000
1000
0
荧光强度
0 2 4 6 8 10 12 14 16 18 20 22 24 26 28 30 32 34 36 38 40 42
循环数

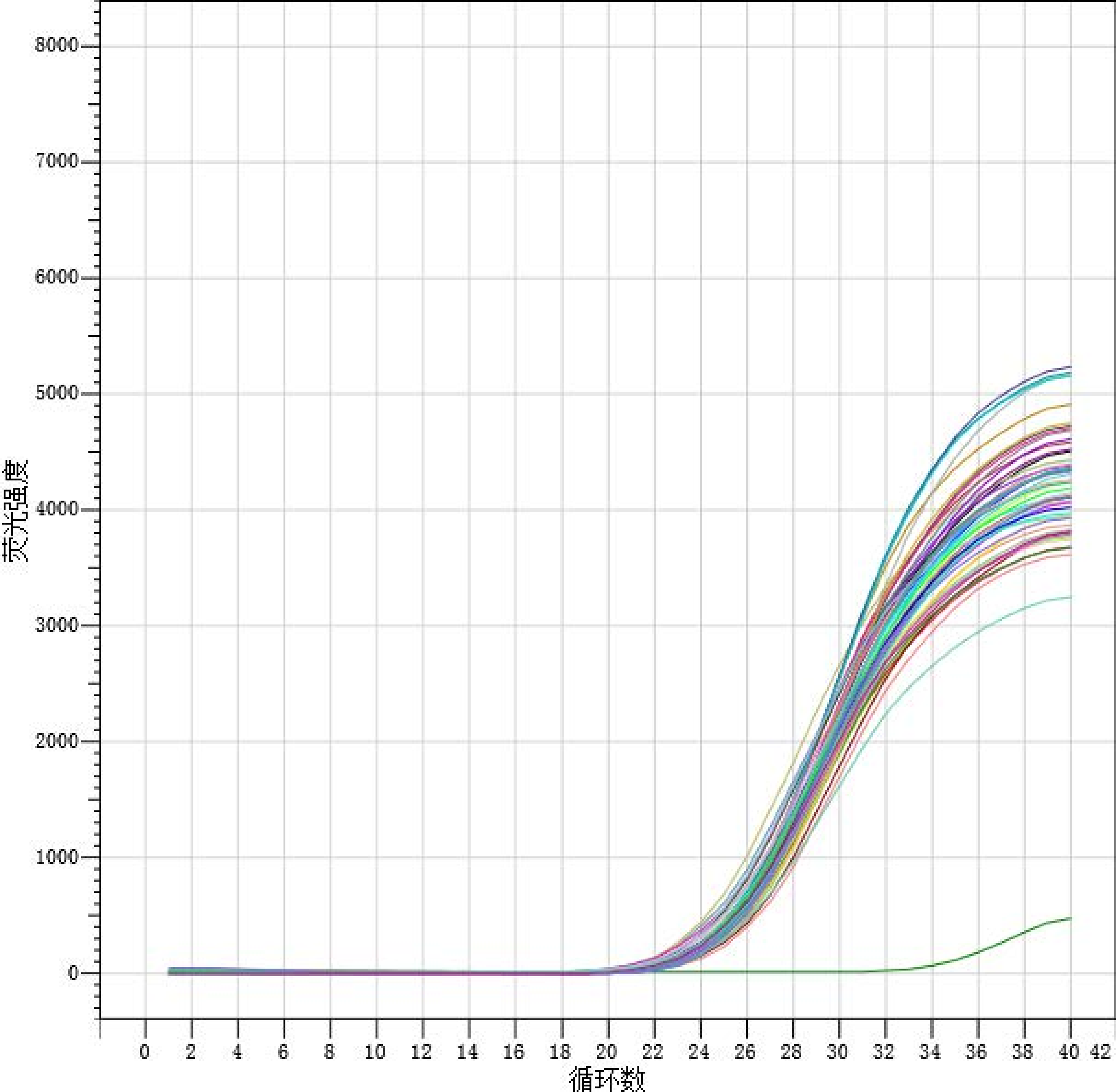

荧光强度
循环数
8000
7000
6000
5000
4000
3000
2000
1000
0
0 2 4 6 8 10 12 14 16 18 20 22 24 26 28 30 32 34 36 38 40 42

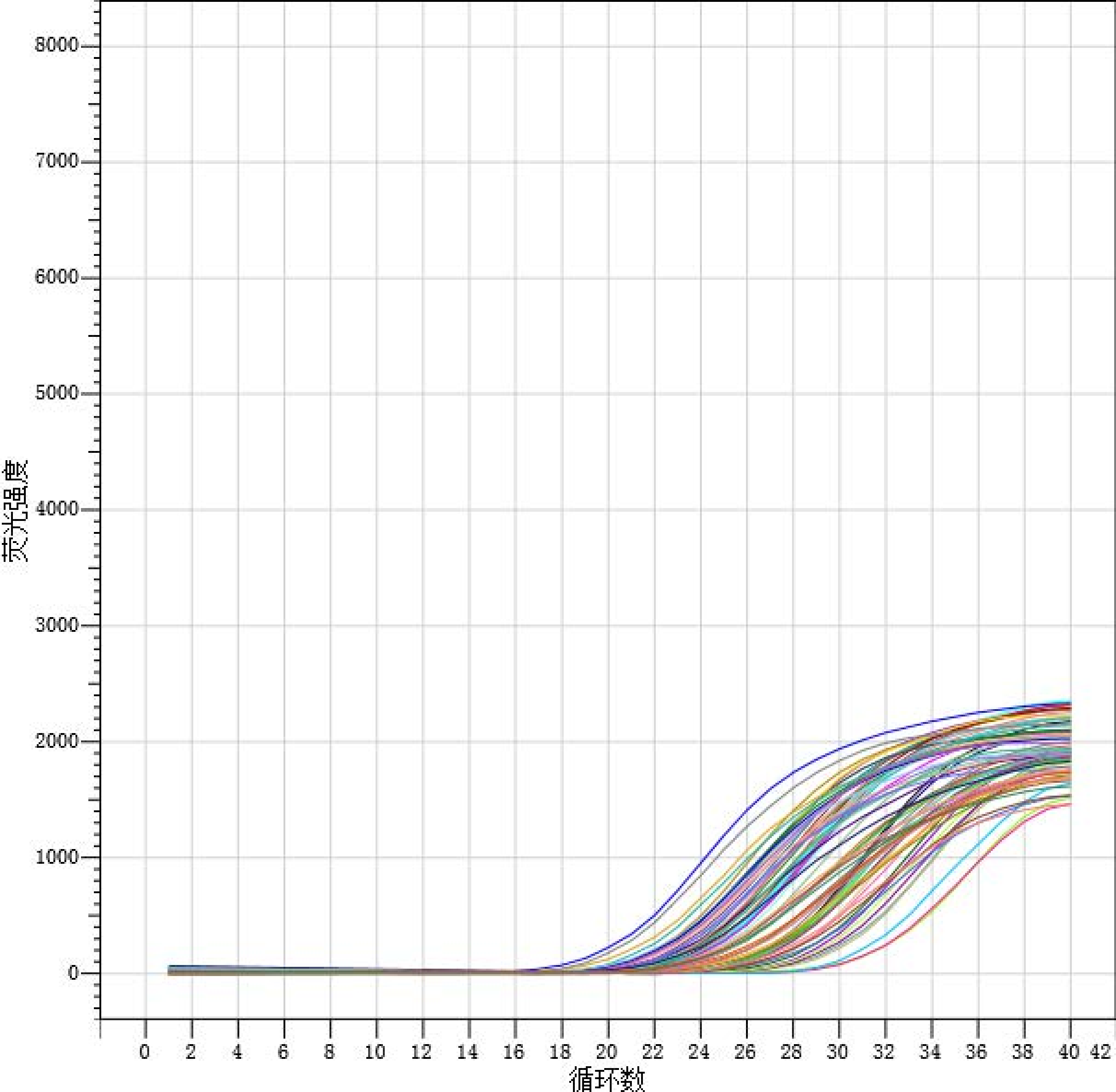

8000
7000
6000
5000
4000
3000
2000
1000
0
荧光强度
0
2
4
6
8
10
12
14
16
18
20
22
24
26
28
30
32
34
36
38
40
42
循环数

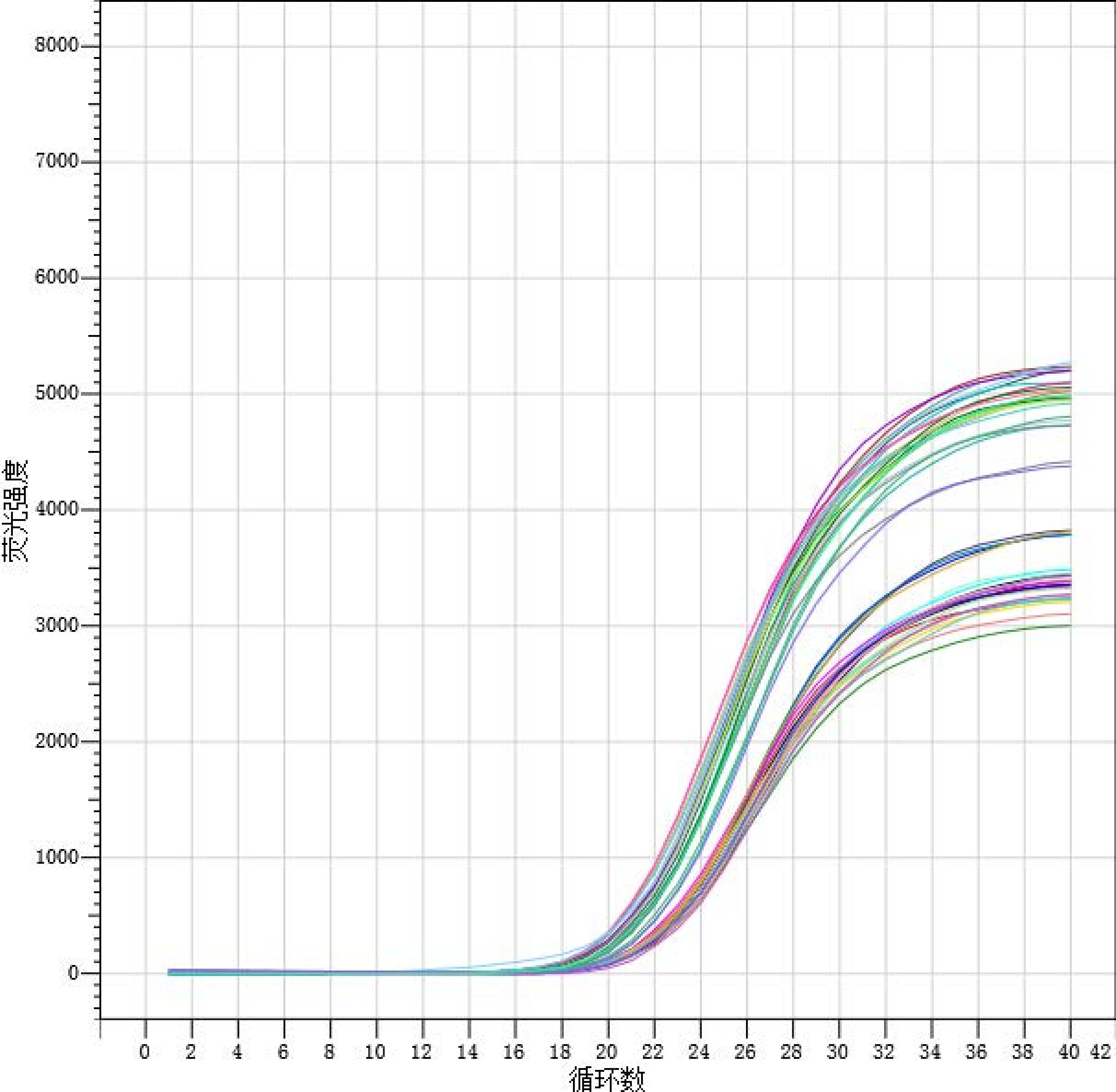
8000
7000
6000
5000
4000
3000
2000
1000
0
荧光强度
0 2 4 6 8 10 12 14 16 18 20 22 24 26 28 30 32 34 36 38 40 42
循环数

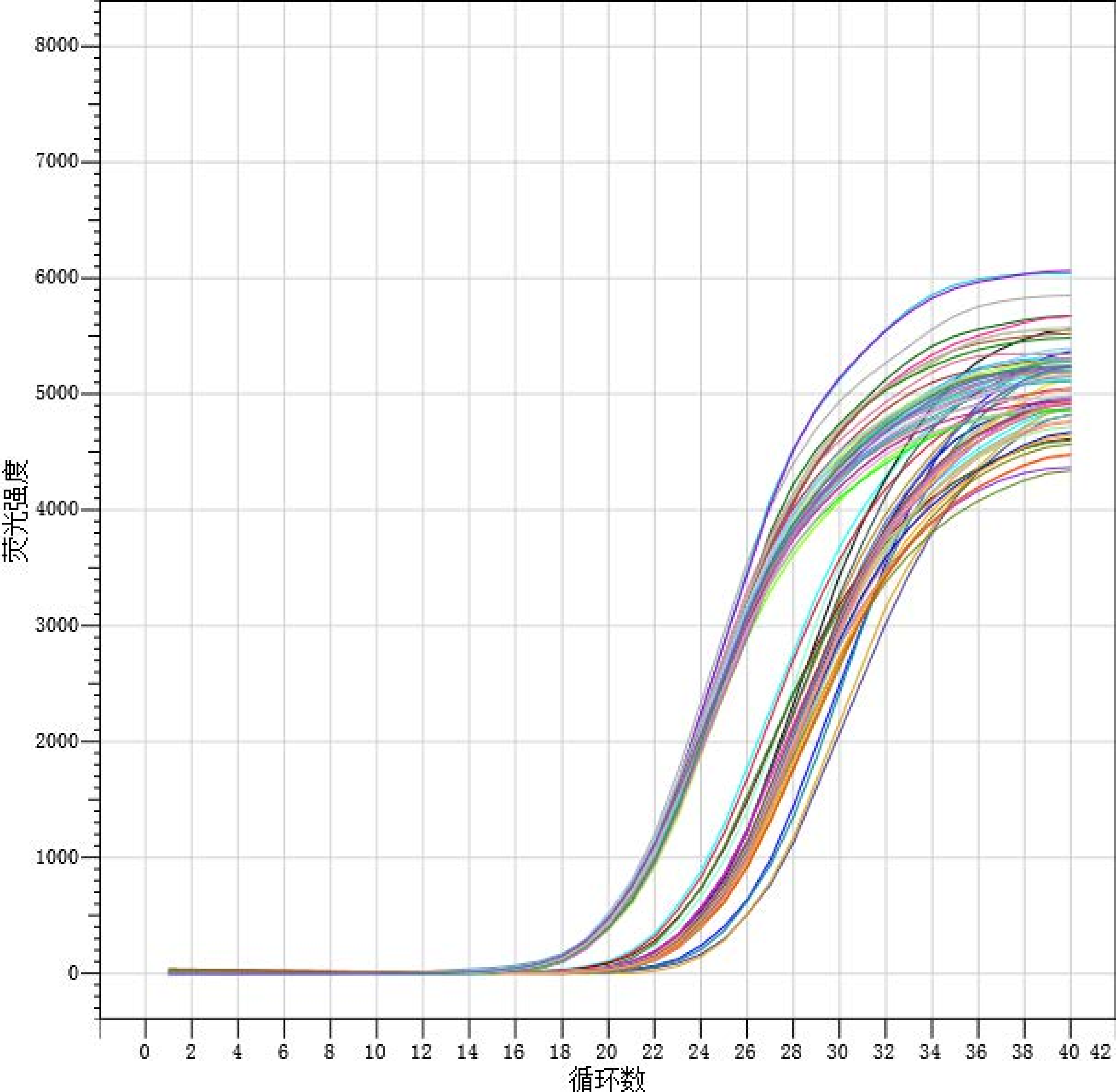

8000
7000
6000
5000
4000
3000
2000
1000
0
荧光强度
0 2 4 6 8 10 12 14 16 18 20 22 24 26 28 30 32 34 36 38 40 42
循环数

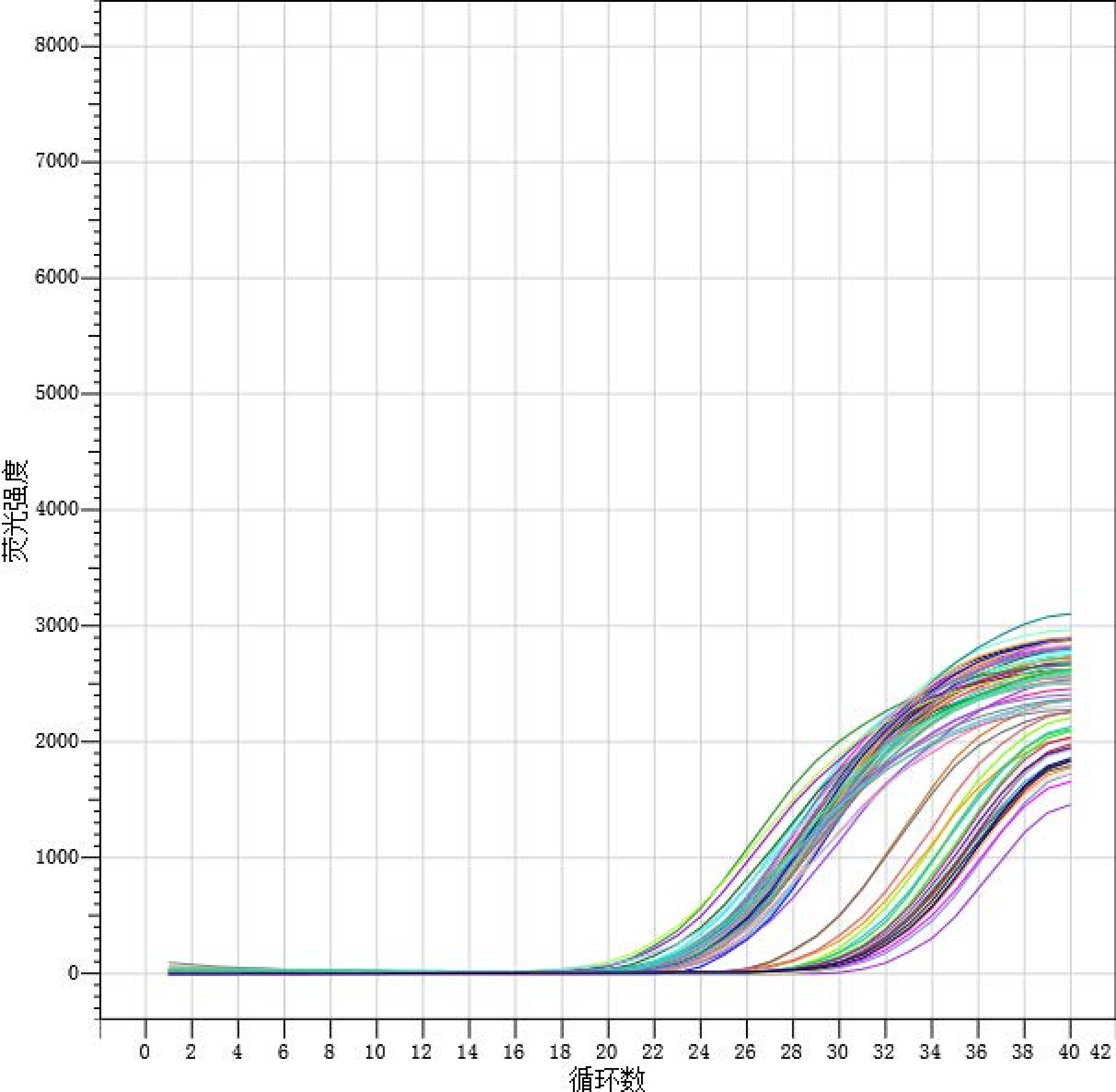

8000
7000
6000
5000
4000
3000
2000
1000
0
荧光强度
0 2 4 6 8 10 12 14 16 18 20 22 24 26 28 30 32 34 36 38 40 42
循环数

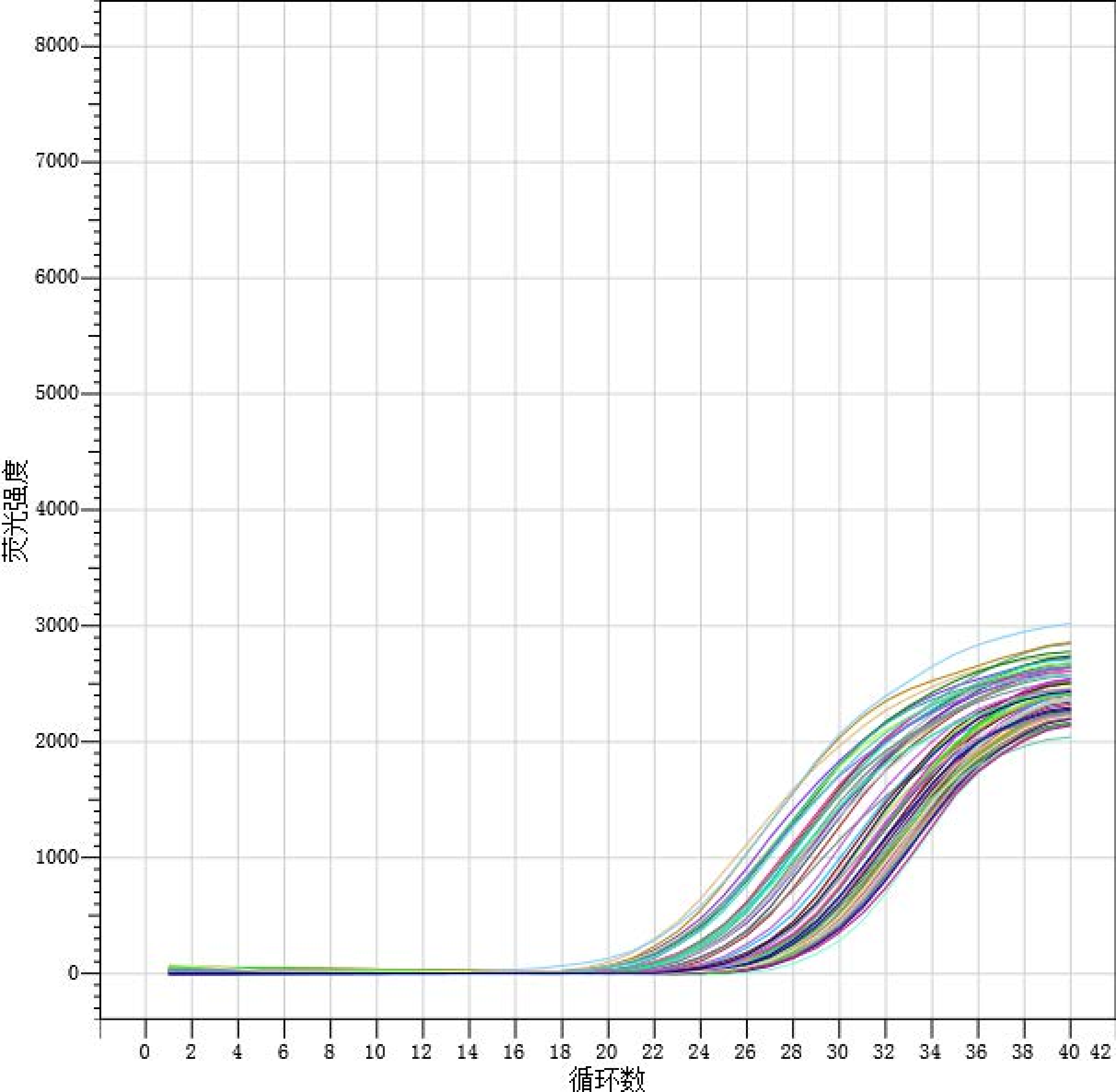

8000
7000
6000
5000
4000
3000
2000
1000
0
荧光强度
0
2
4
6
8
10
12
14
16
18
20
22
24
26
28
30
32
34
36
38
40
42
循环数

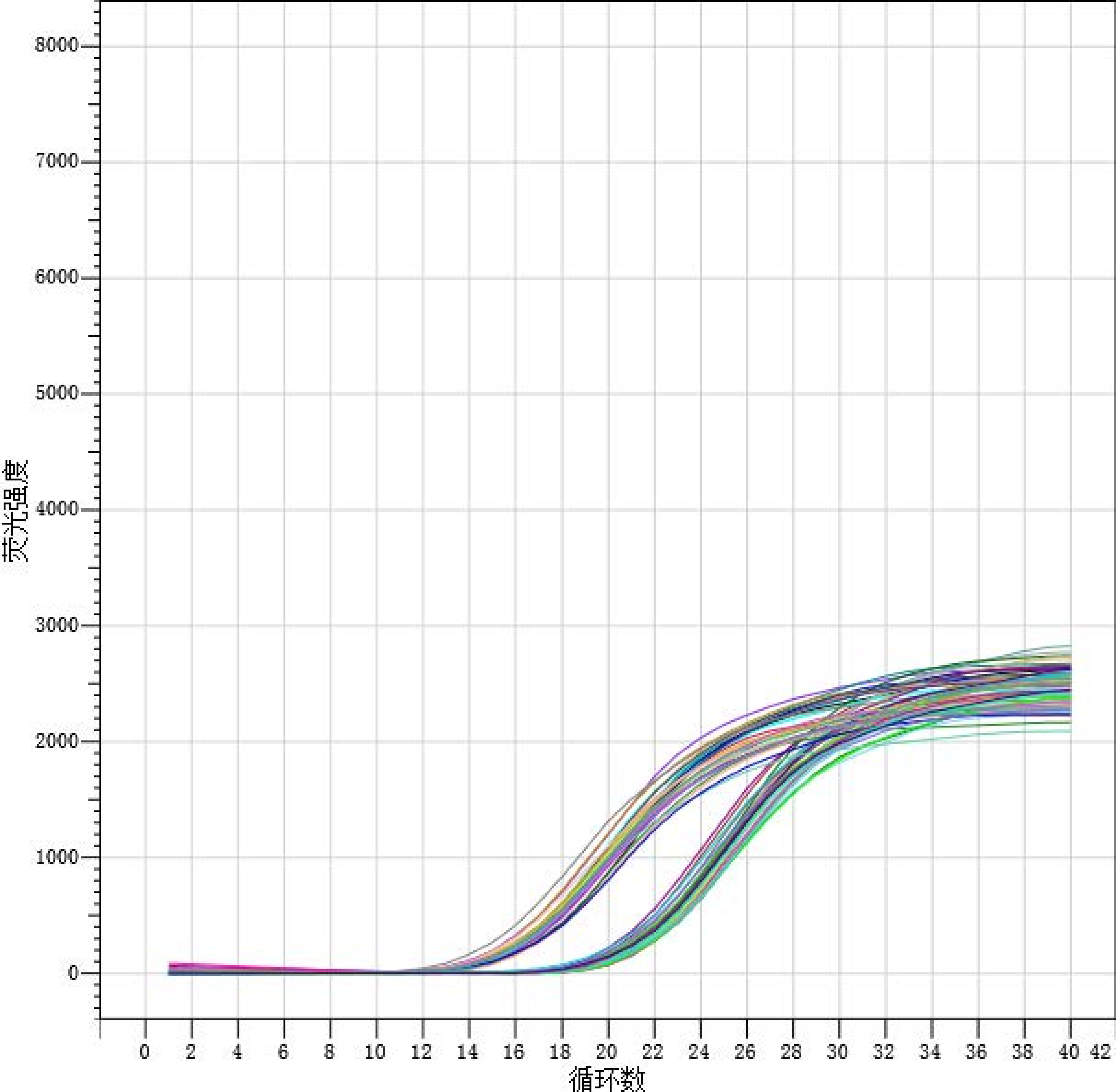
8000
7000
6000
5000
4000
3000
2000
1000
0
荧光强度
0
2
4
6
8
10
12
14
16
18
20
22
24
26
28
30
32
34
36
38
40
42
循环数

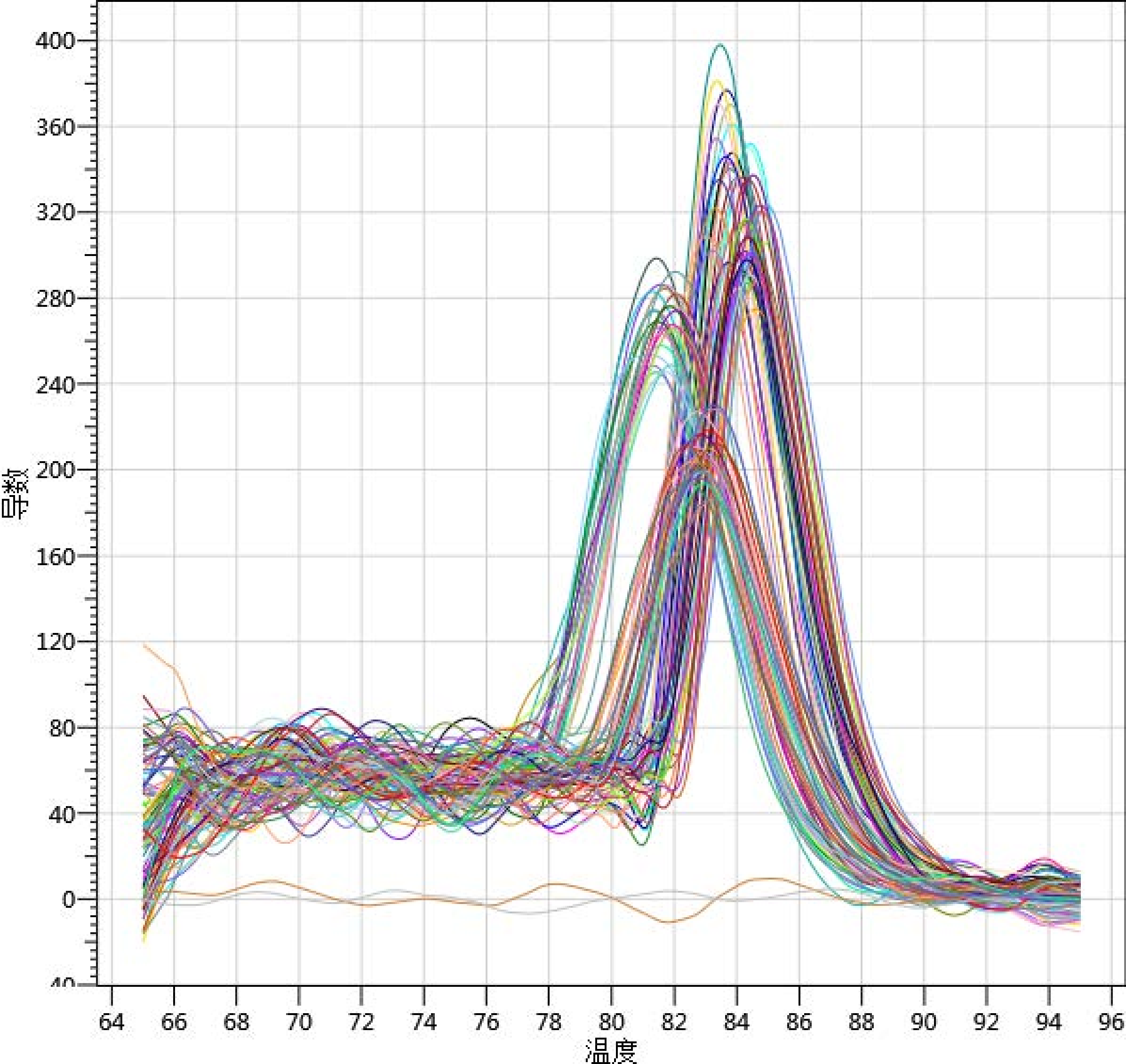

400
360
320
280
240
200
160
120
80
40
0
-40
导数
64
66
68
70
72
74
76
78
80
82
84
86
88
90
92
94
96
温度

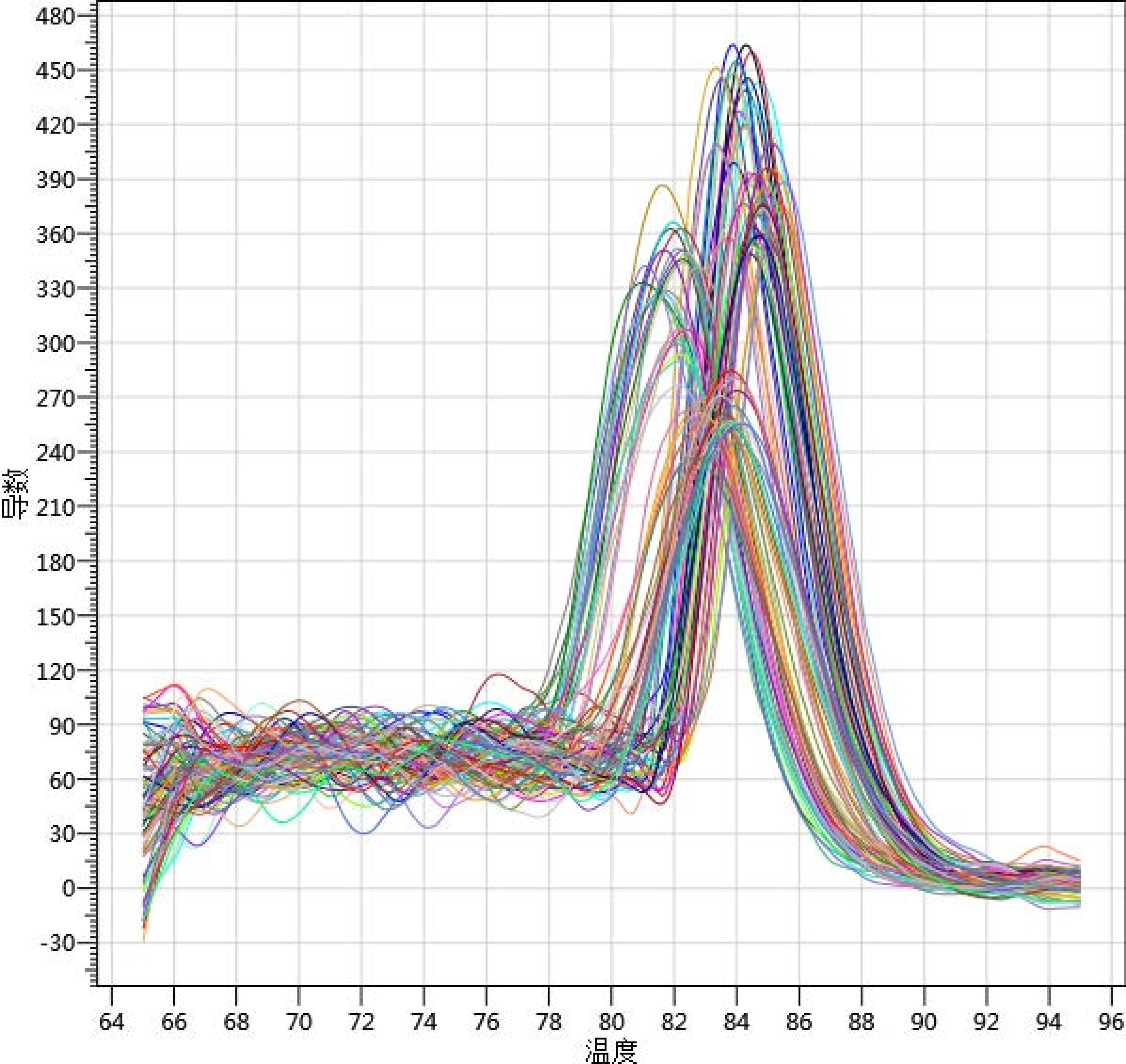

480
450
420
390
360
330
300
270
240
210
180
150
120
90
60
30
0
-30
导数
64
66
68
70
72
74
76
78
80
82
84
86
88
90
92
94
96
温度

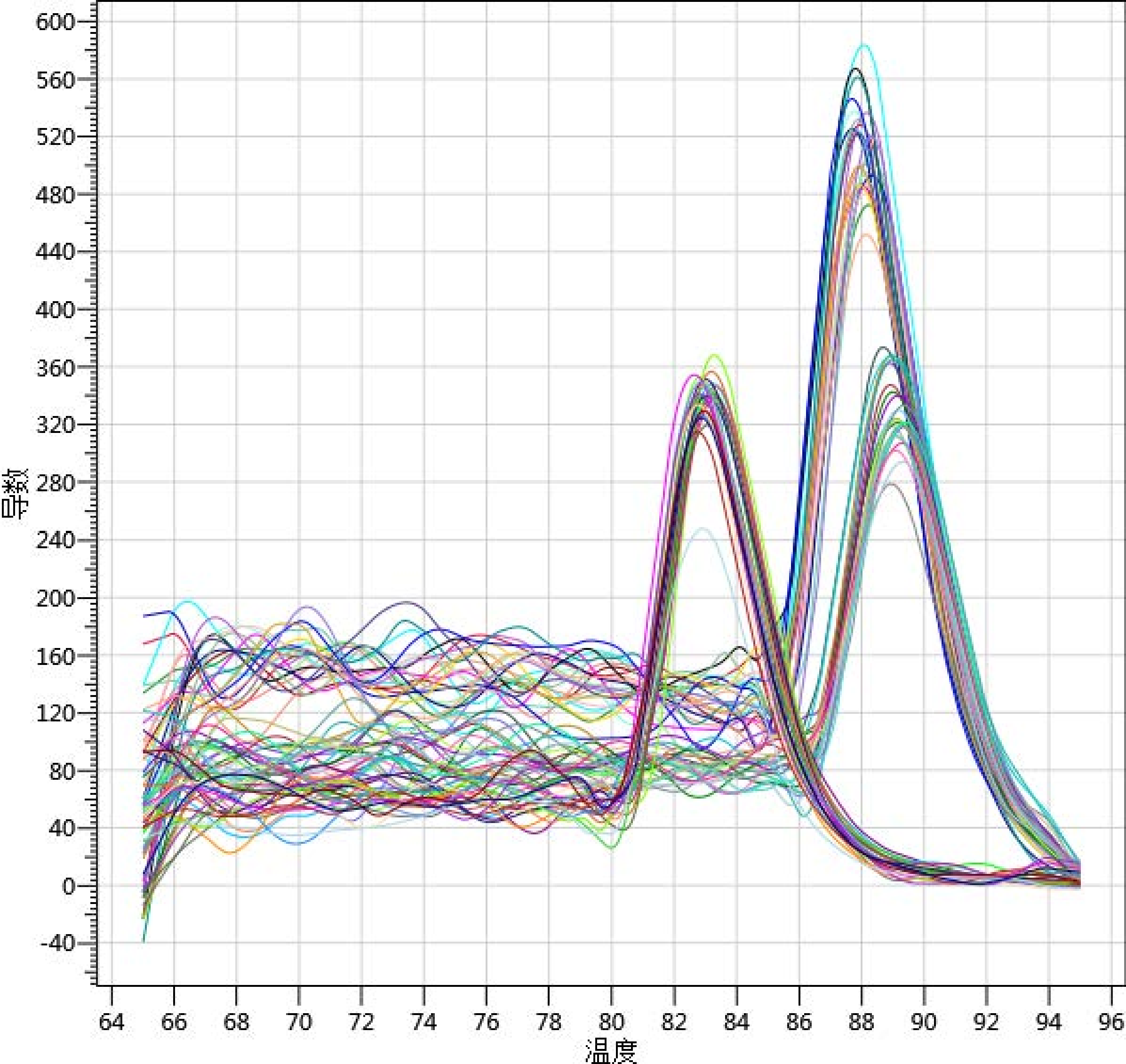

导数
温度
600
560
520
480
440
400
360
320
280
240
200
160
120
80
40
0
-40
64
66
68
70
72
74
76
78
80
82
84
86
88
90
92
94
96

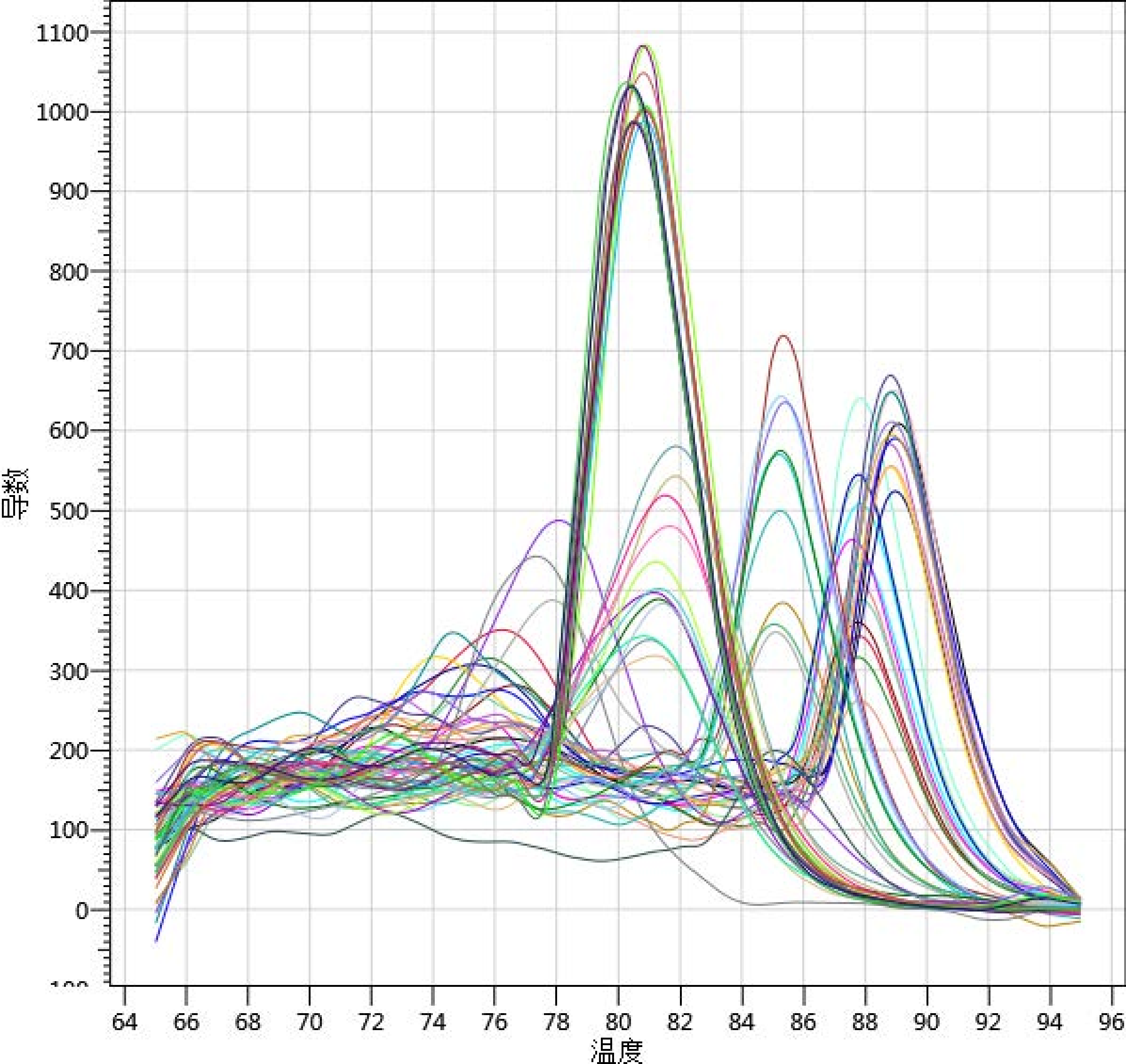

导数
1100
1000
900
800
700
600
500
400
300
200
100
0
64
66
68
70
72
74
76
78
80
82
84
86
88
90
92
94
96
温度

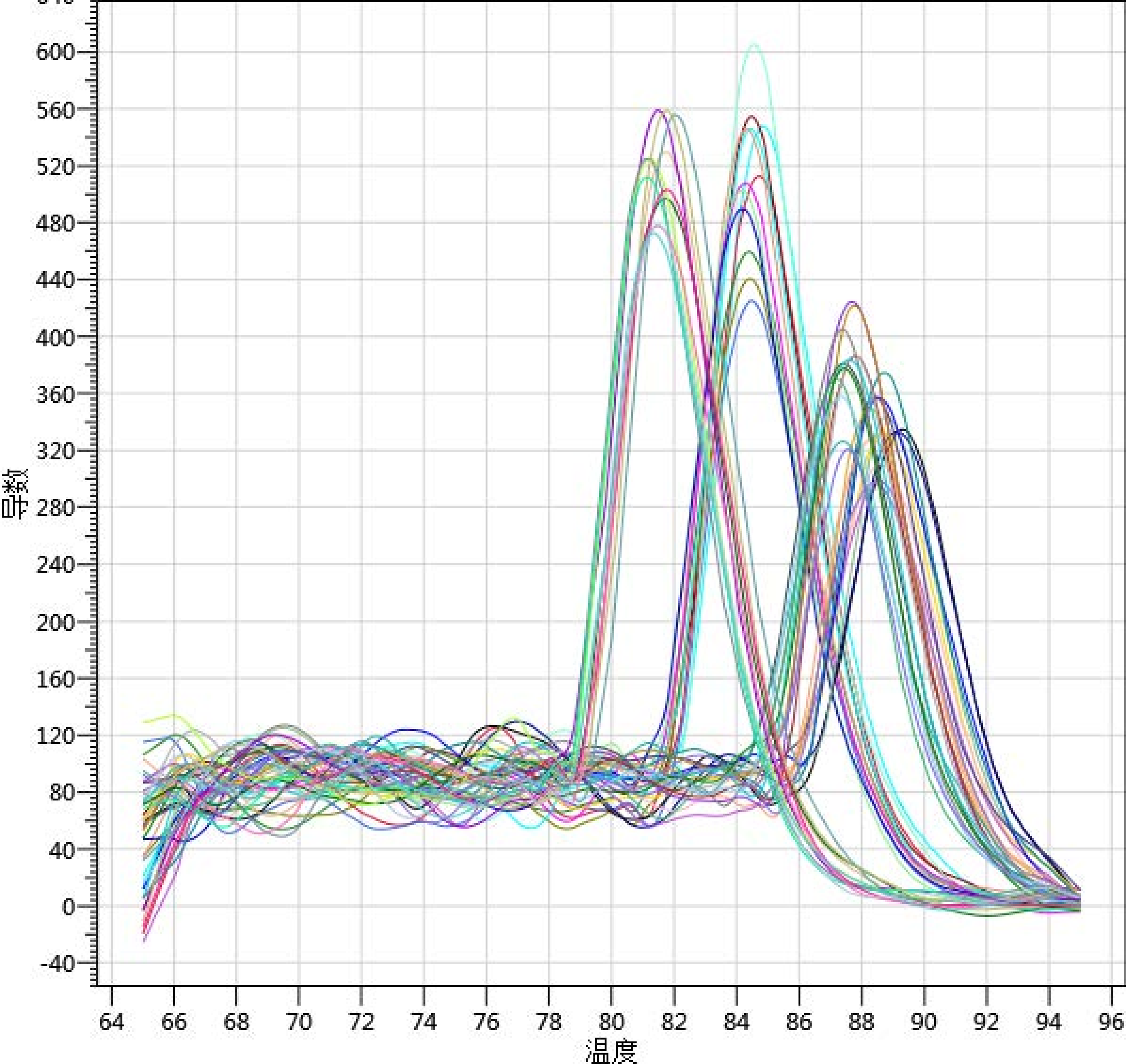

600
560
520
480
440
400
360
320
280
240
200
160
120
80
40
0
-40
导数
64
66
68
70
72
74
76
78
80
82
84
86
88
90
92
94
96
温度

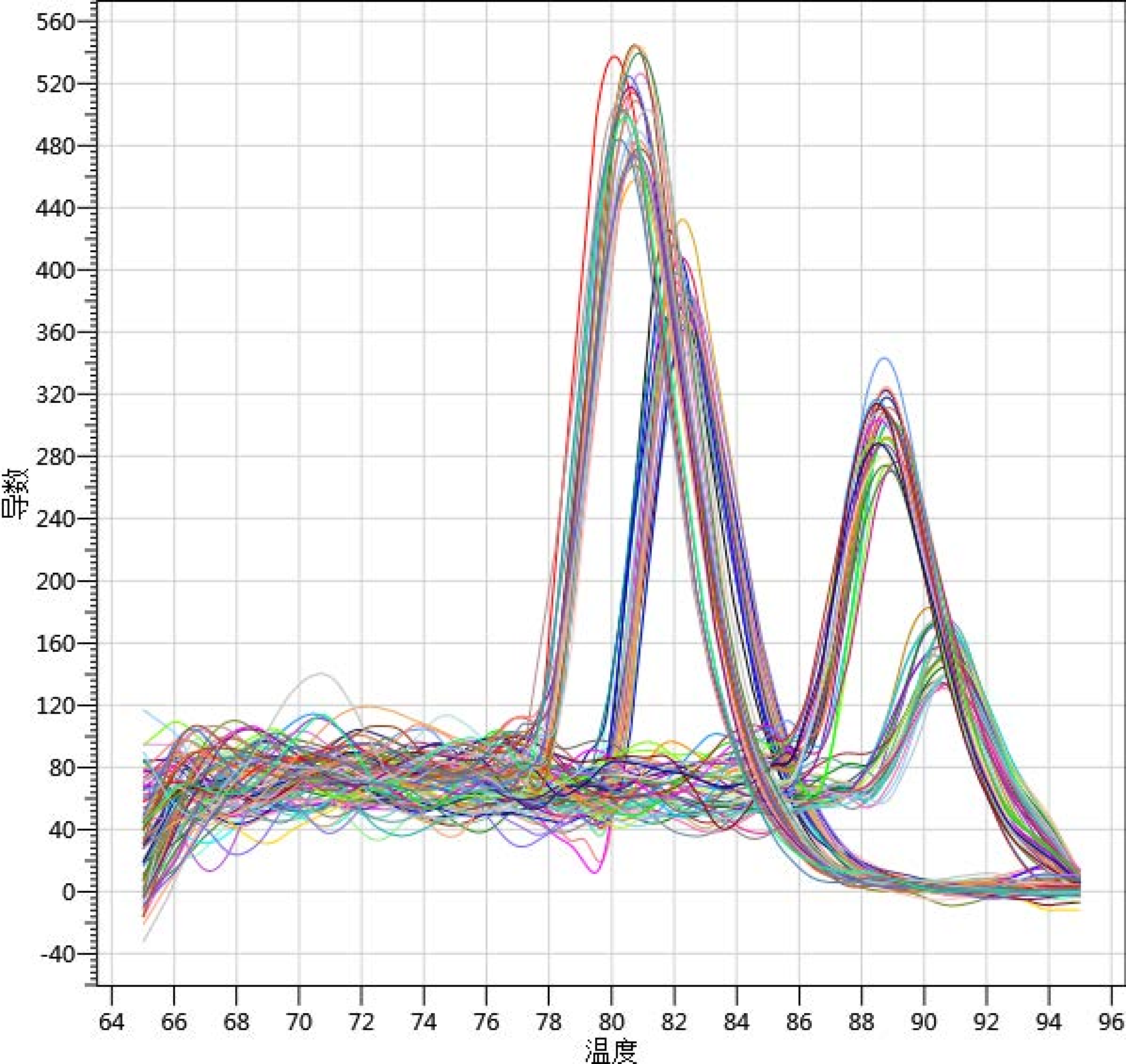

560
520
480
440
400
360
320
280
240
200
160
120
80
40
0
-40
导数
64
66
68
70
72
74
76
78
80
82
84
86
88
90
92
94
96
温度

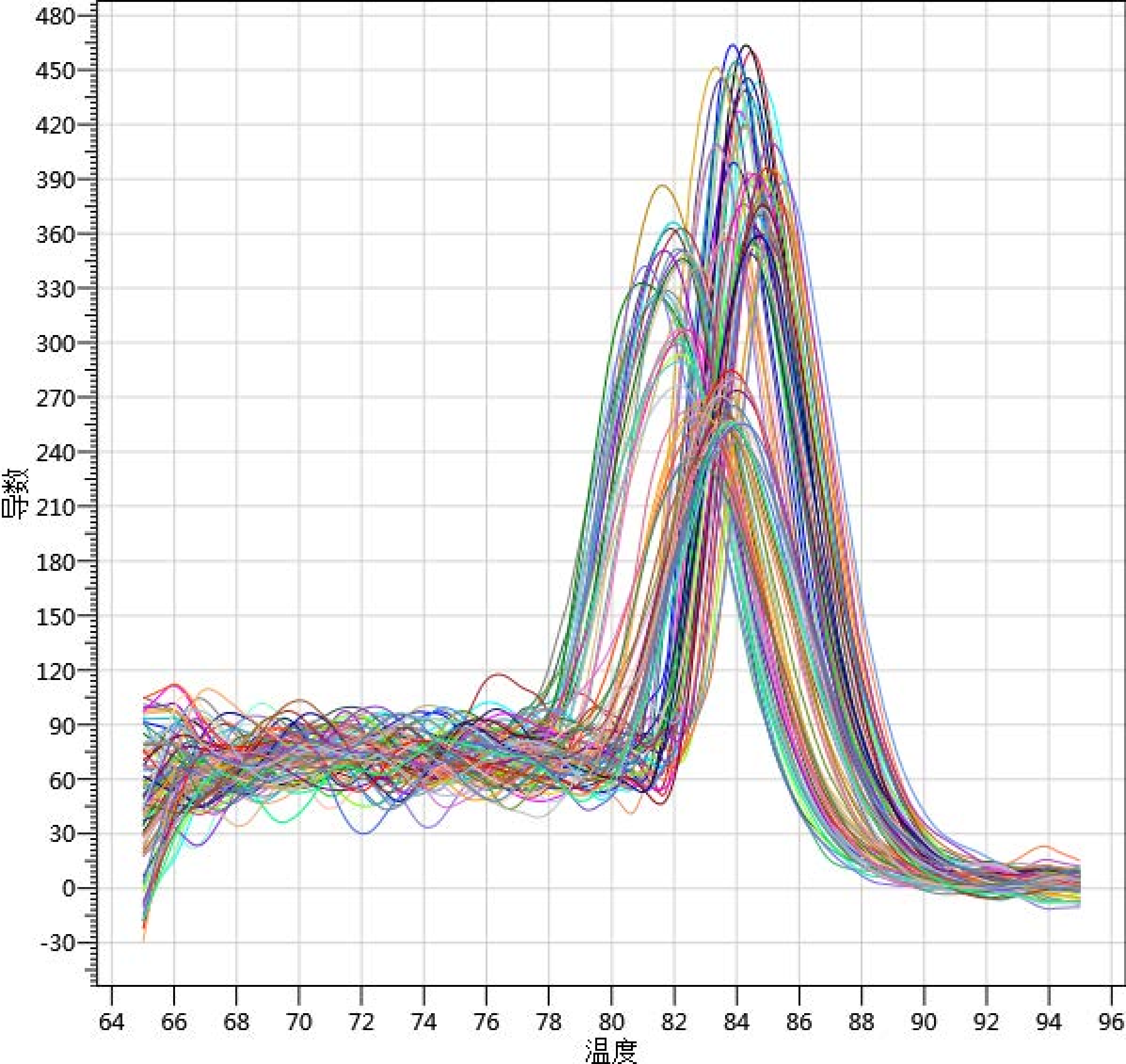

480
450
420
390
360
330
300
270
240
210
180
150
120
90
60
30
0
-30
导数
64
66
68
70
72
74
76
78
80
82
84
86
88
90
92
94
96
温度

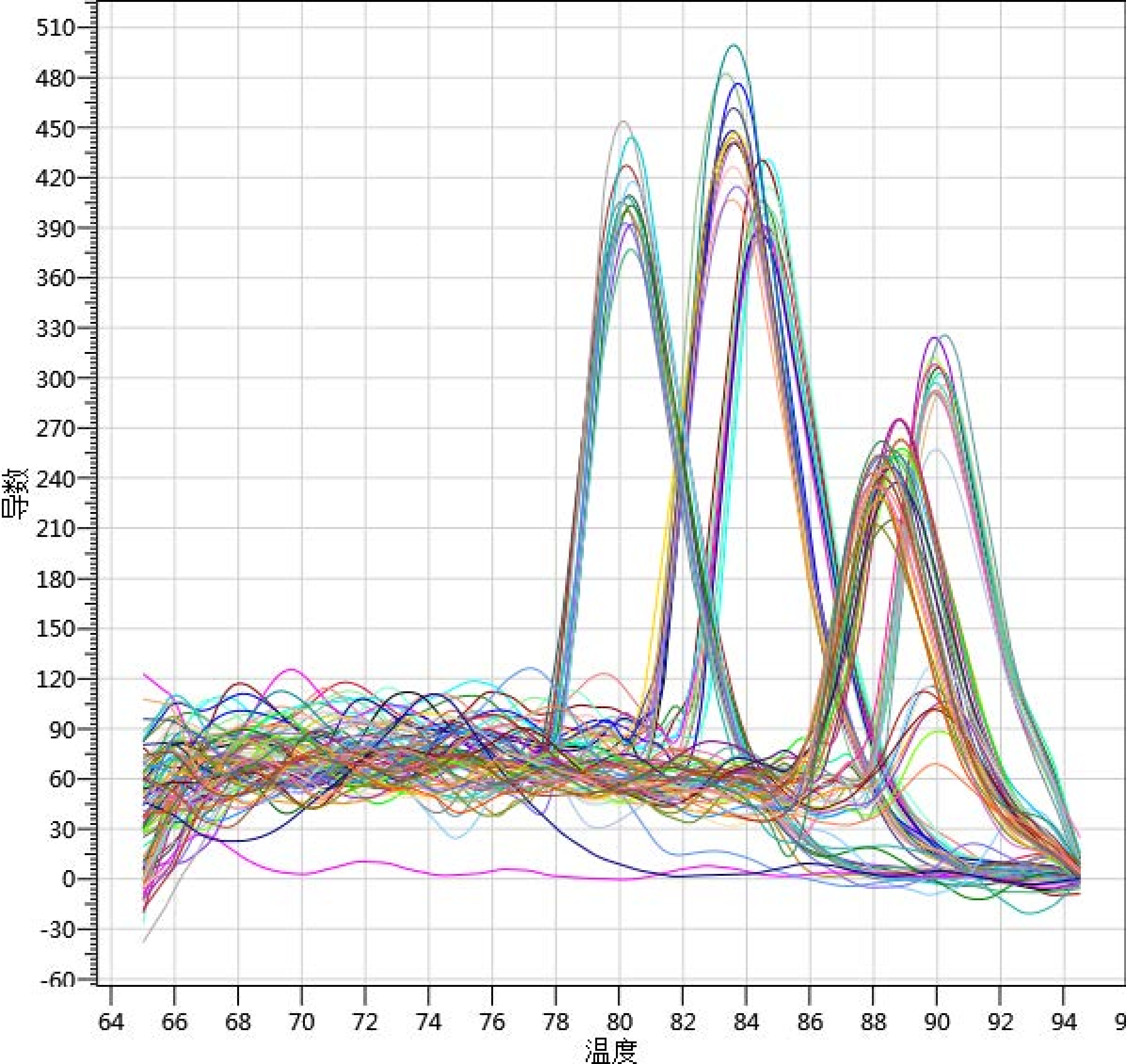

510
480
450
420
390
360
330
300
270
240
210
180
150
120
90
60
30
0
-30
-60
导数
64
66
68
70
72
74
76
78
80
82
84
86
88
90
92
94
温度

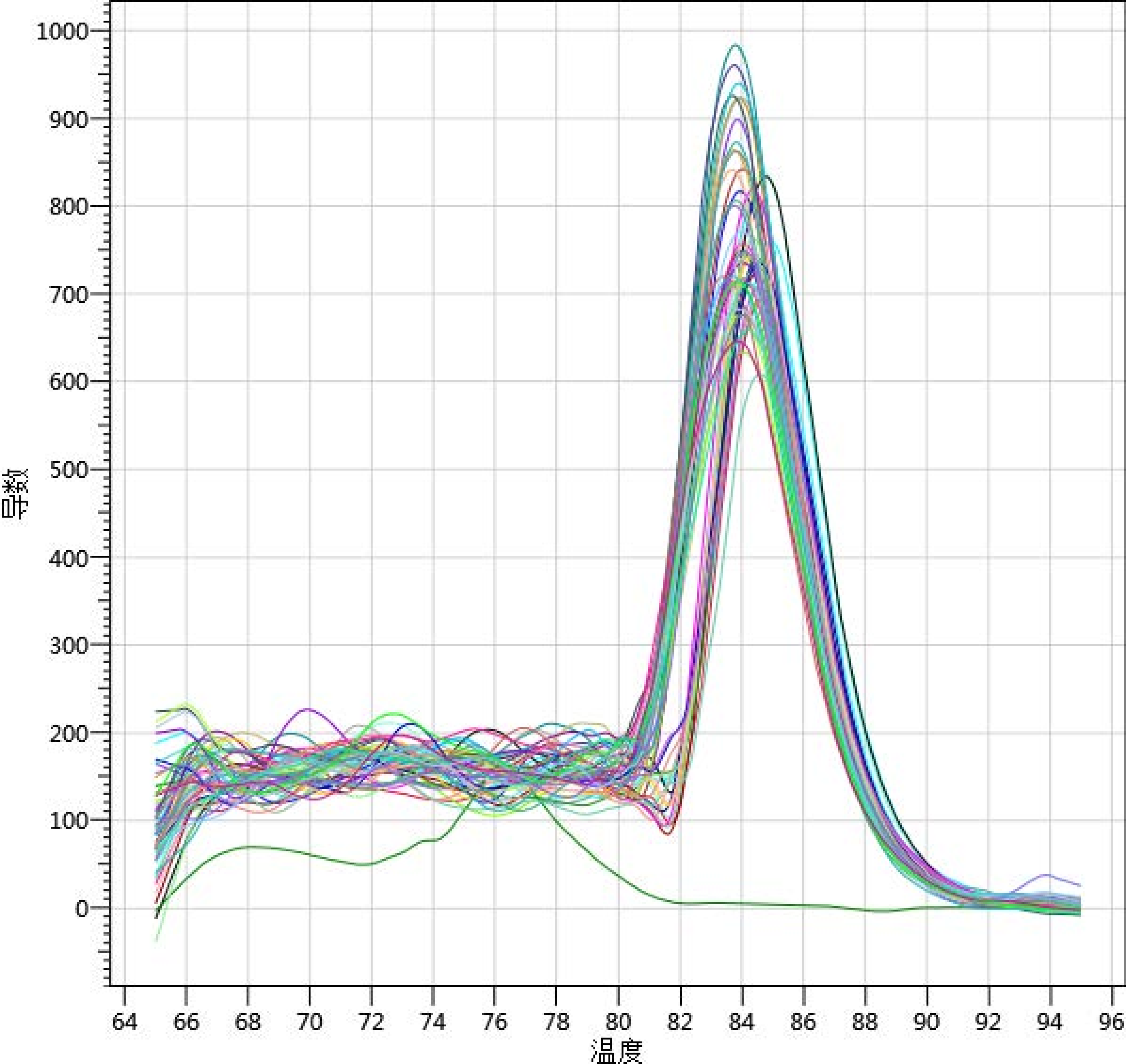

1000
900
800
700
600
500
400
300
200
100
0
导数
64
66
68
70
72
74
76
78
80
82
84
86
88
90
92
94
96
温度

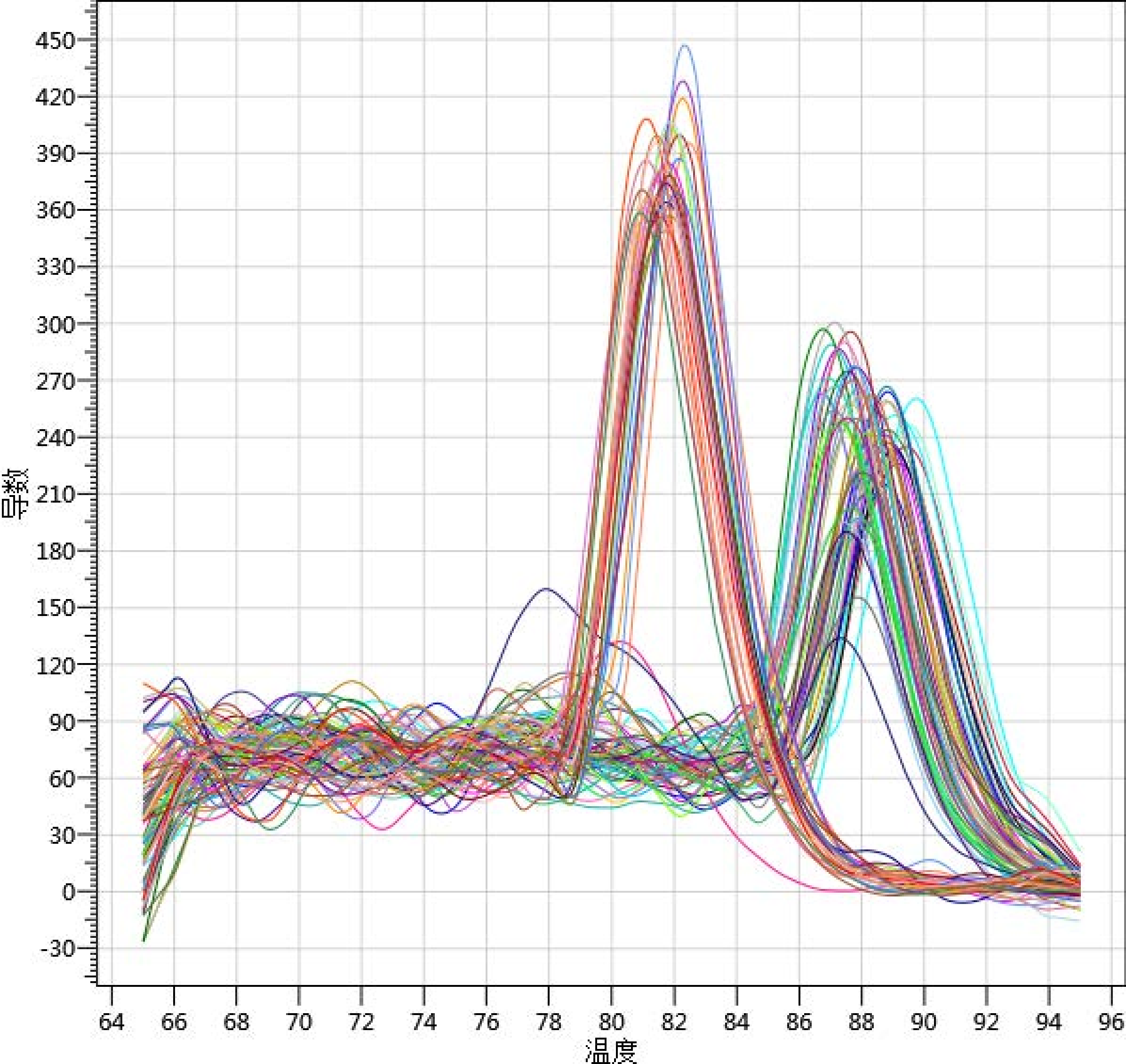

导数
450
420
390
360
330
300
270
240
210
180
150
120
90
60
30
0
-30
64
66
68
70
72
74
76
78
80
82
84
86
88
90
92
94
96
温度

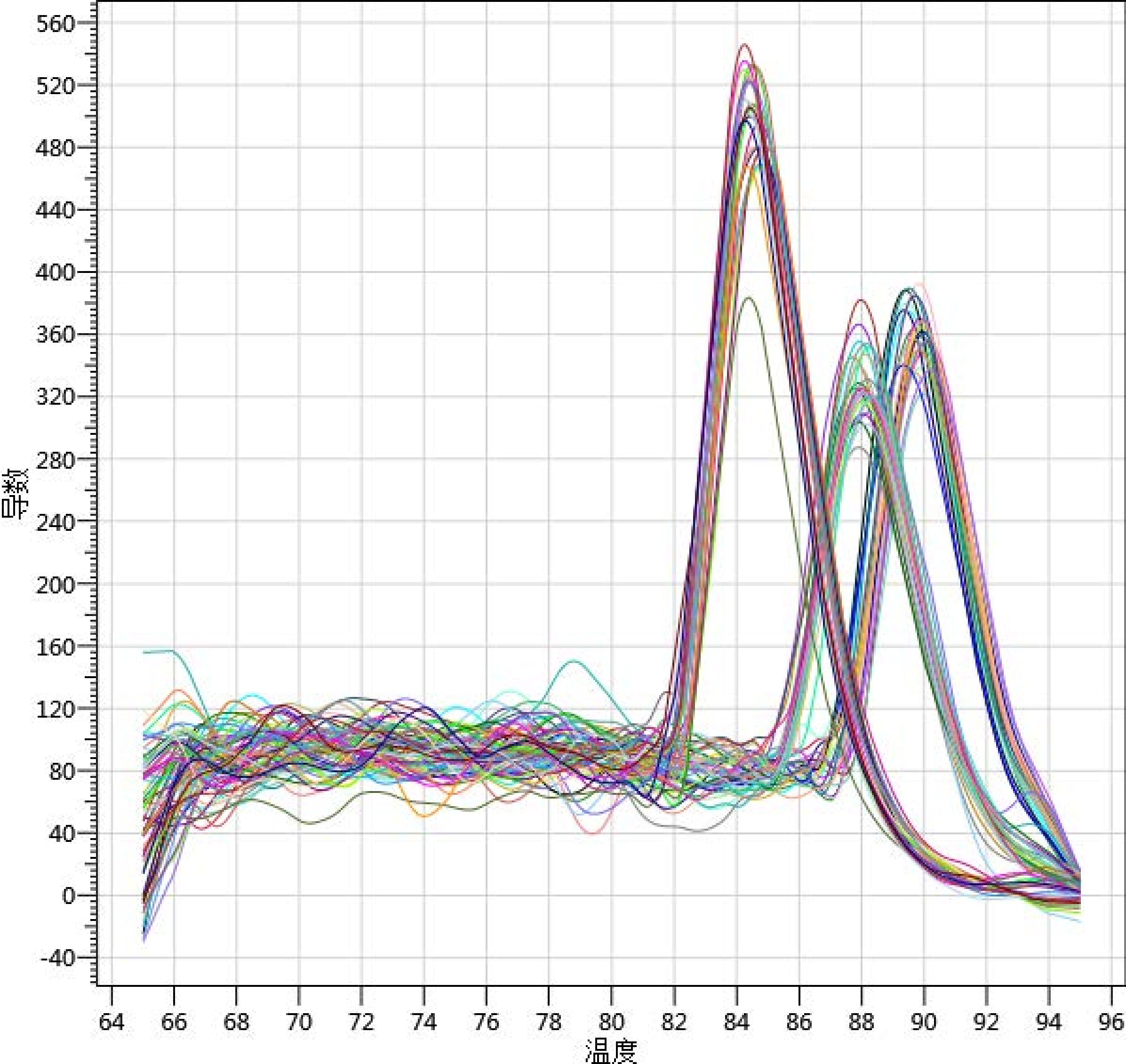

560
520
480
440
400
360
320
280
240
200
160
120
80
40
0
-40
导数
64
66
68
70
72
74
76
78
80
82
84
86
88
90
92
94
96
温度

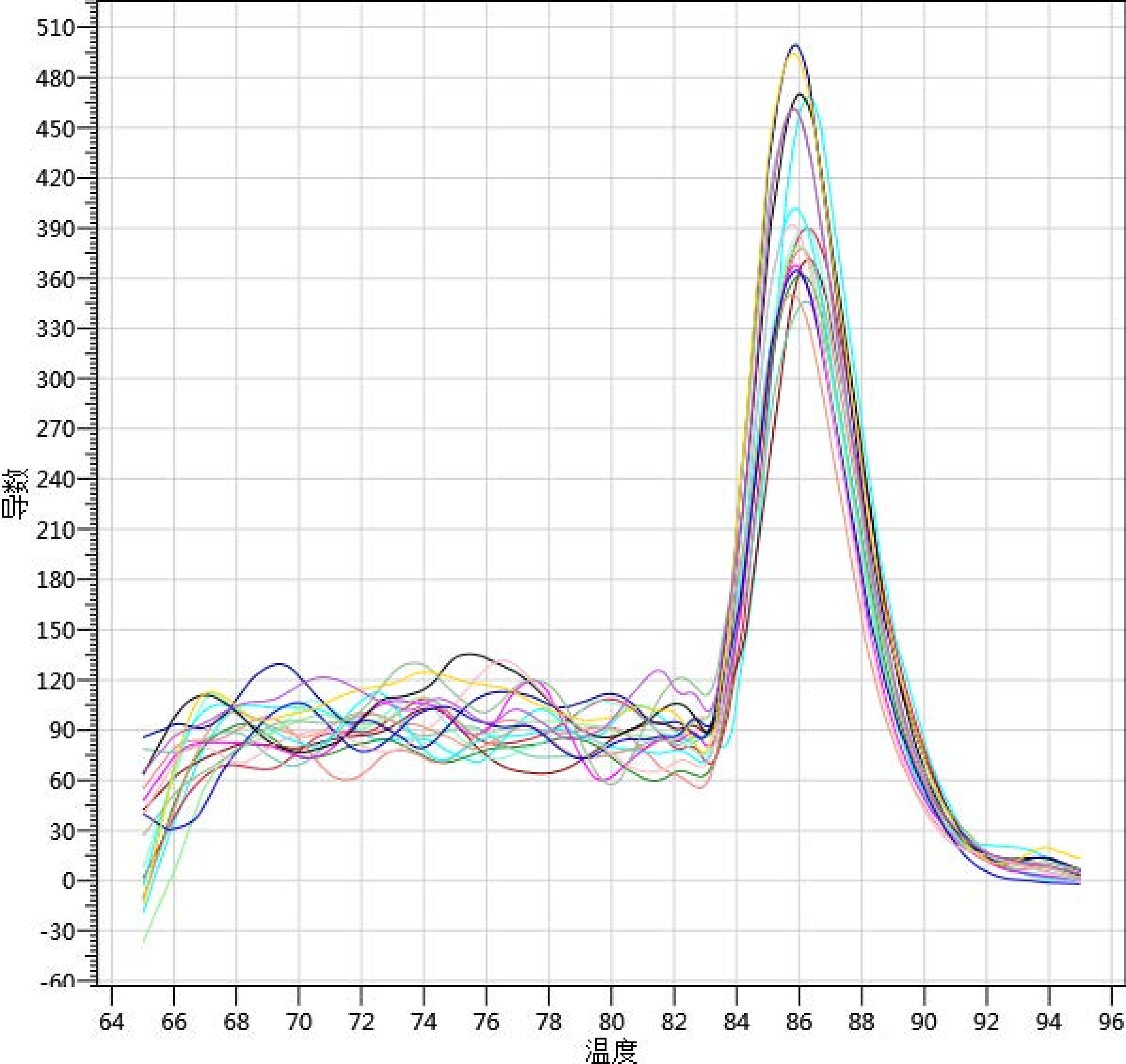

导数
温度
510
480
450
420
390
360
330
300
270
240
210
180
150
120
90
60
30
0
-30
-60
64
66
68
70
72
74
76
78
80
82
84
86
88
90
92
94
96

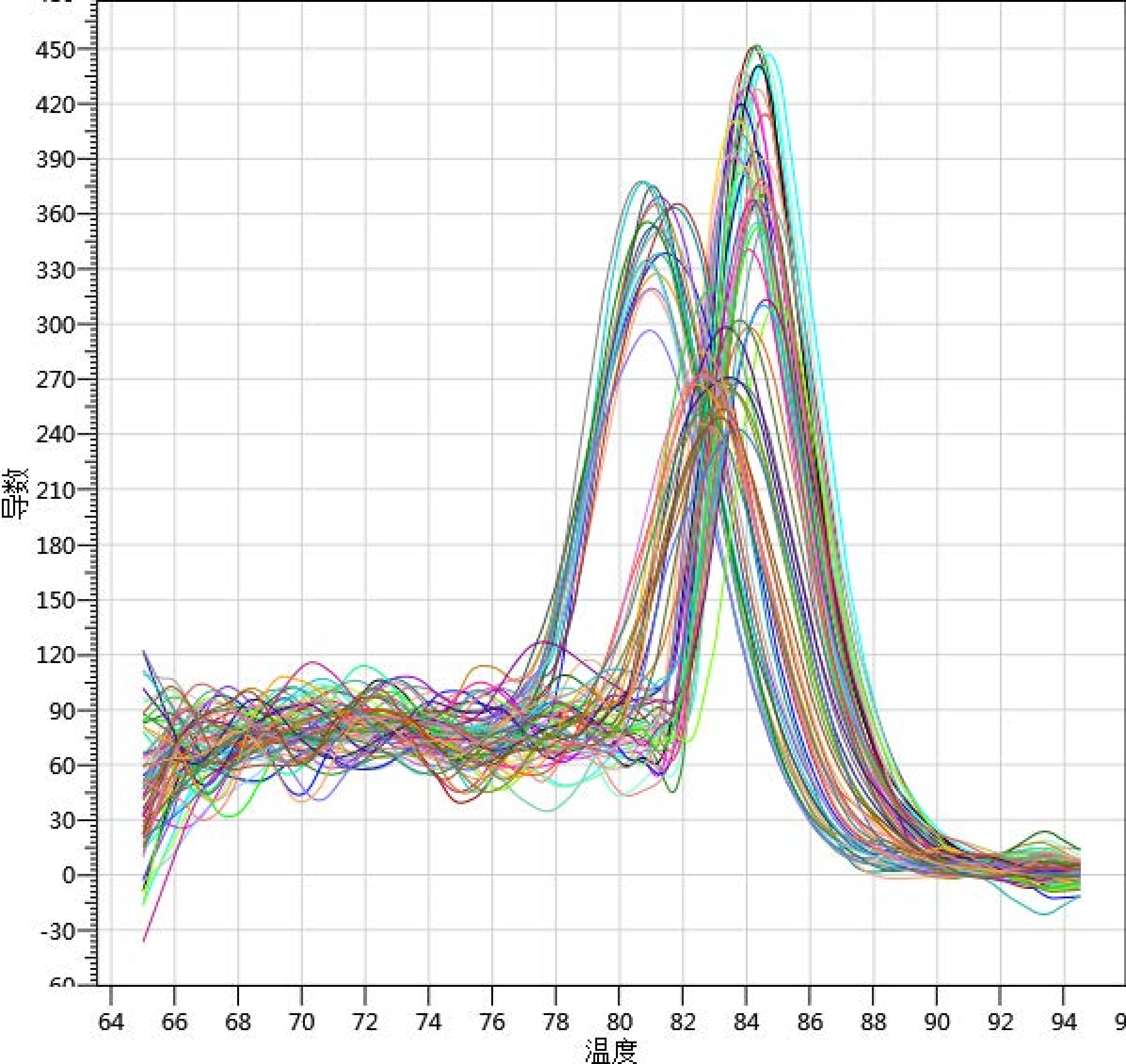

450
420
390
360
330
300
270
240
210
180
150
120
90
60
30
0
-30
导数
64
66
68
70
72
74
76
78
80
82
84
86
88
90
92
94
温度

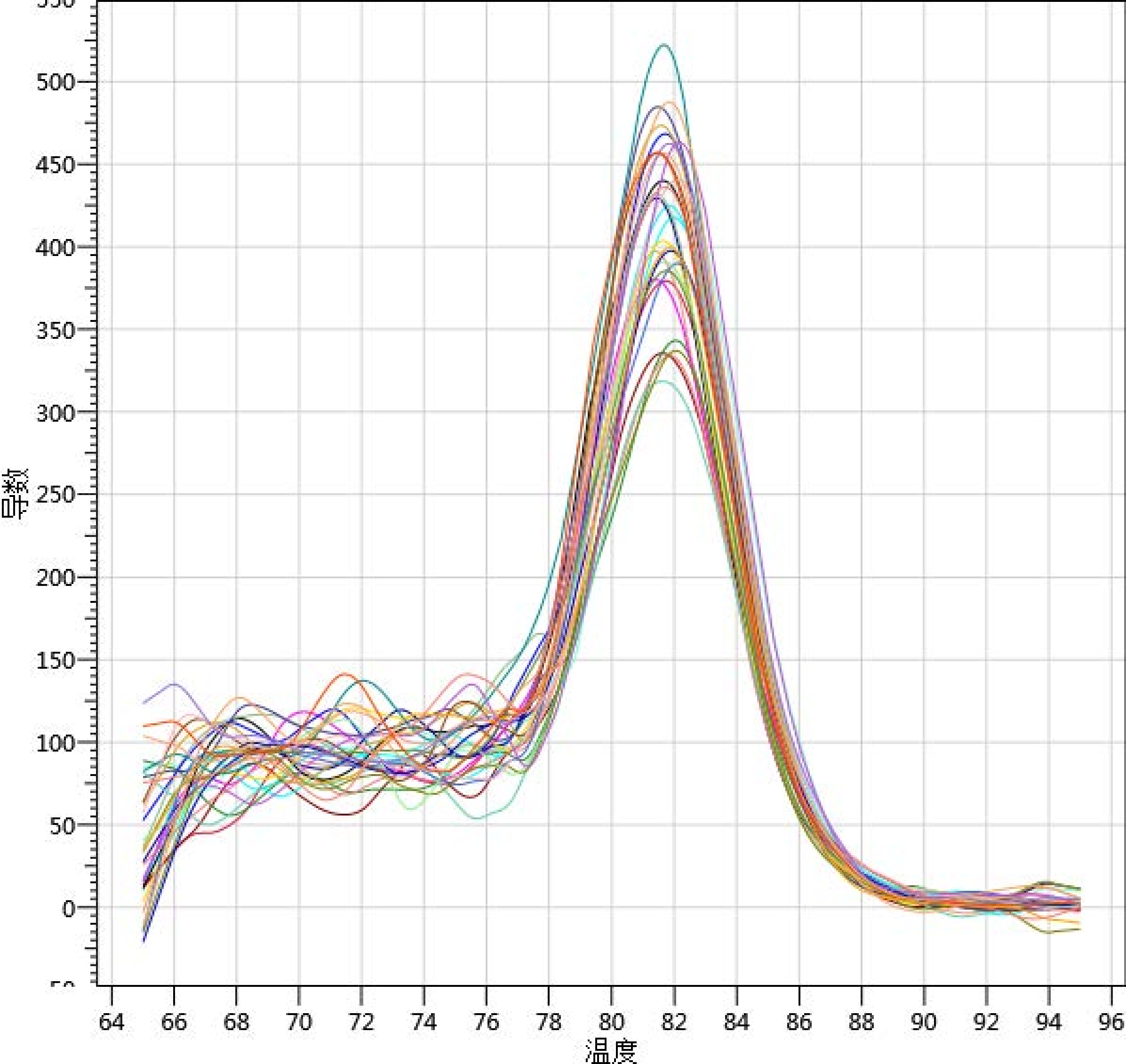

500
450
400
350
300
250
200
150
100
50
0
导数
64
66
68
70
72
74
76
78
80
82
84
86
88
90
92
94
96
温度

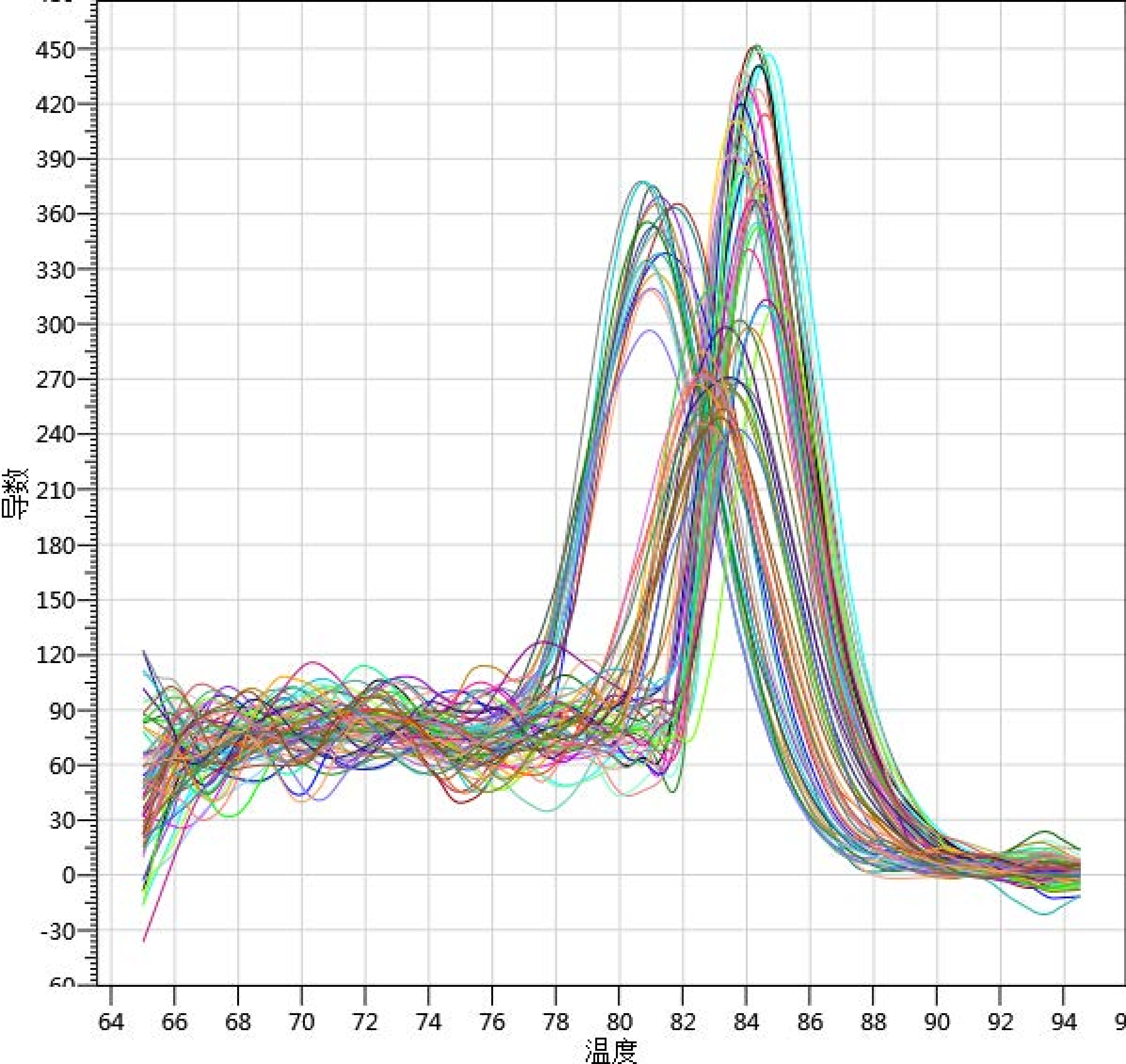

导数
温度
450
420
390
360
330
300
270
240
210
180
150
120
90
60
30
0
-30
64
66
68
70
72
74
76
78
80
82
84
86
88
90
92
94

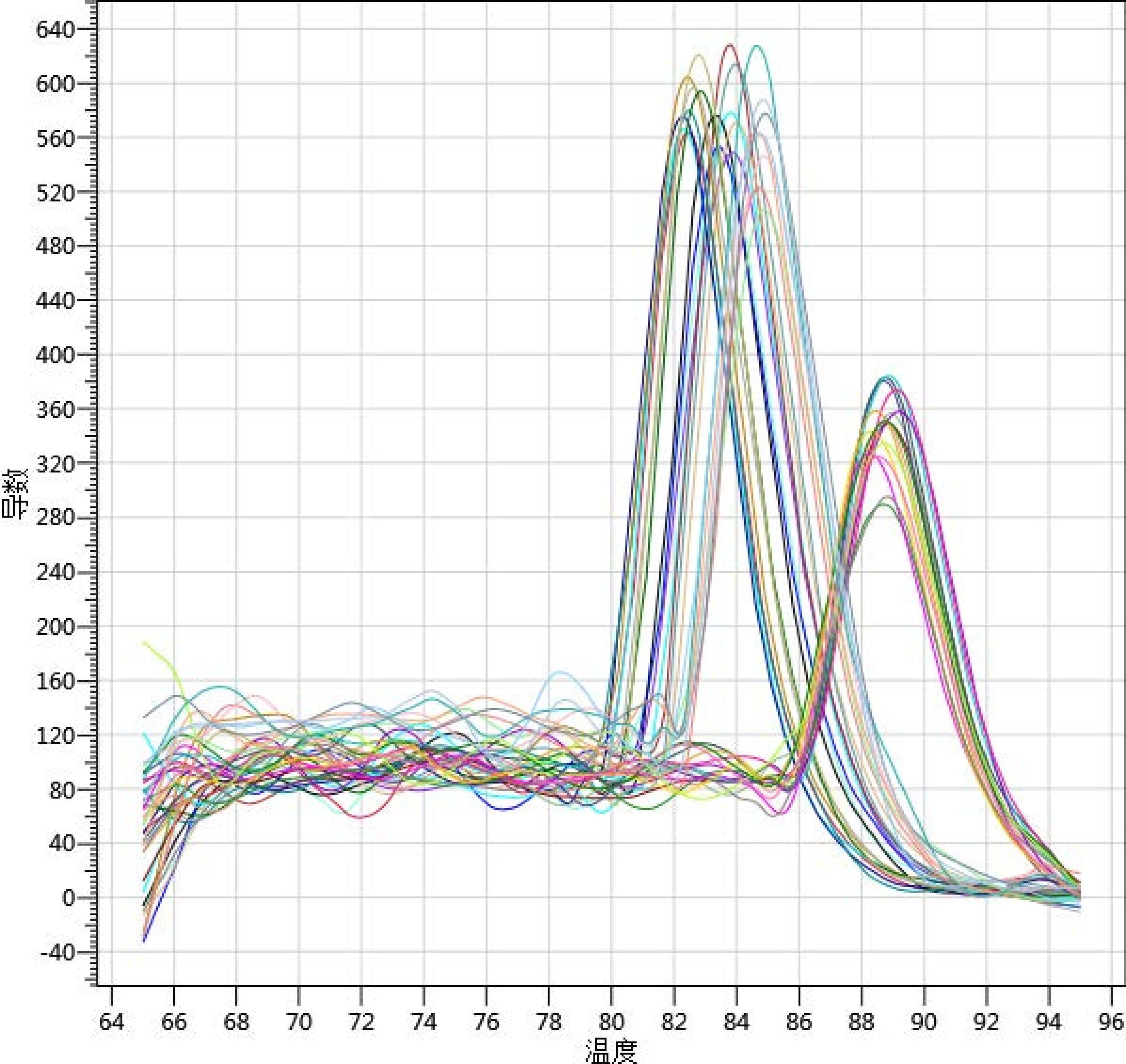

640
600
560
520
480
440
400
360
320
280
240
200
160
120
80
40
0
-40
导数
64
66
68
70
72
74
76
78
80
82
84
86
88
90
92
94
96
温度

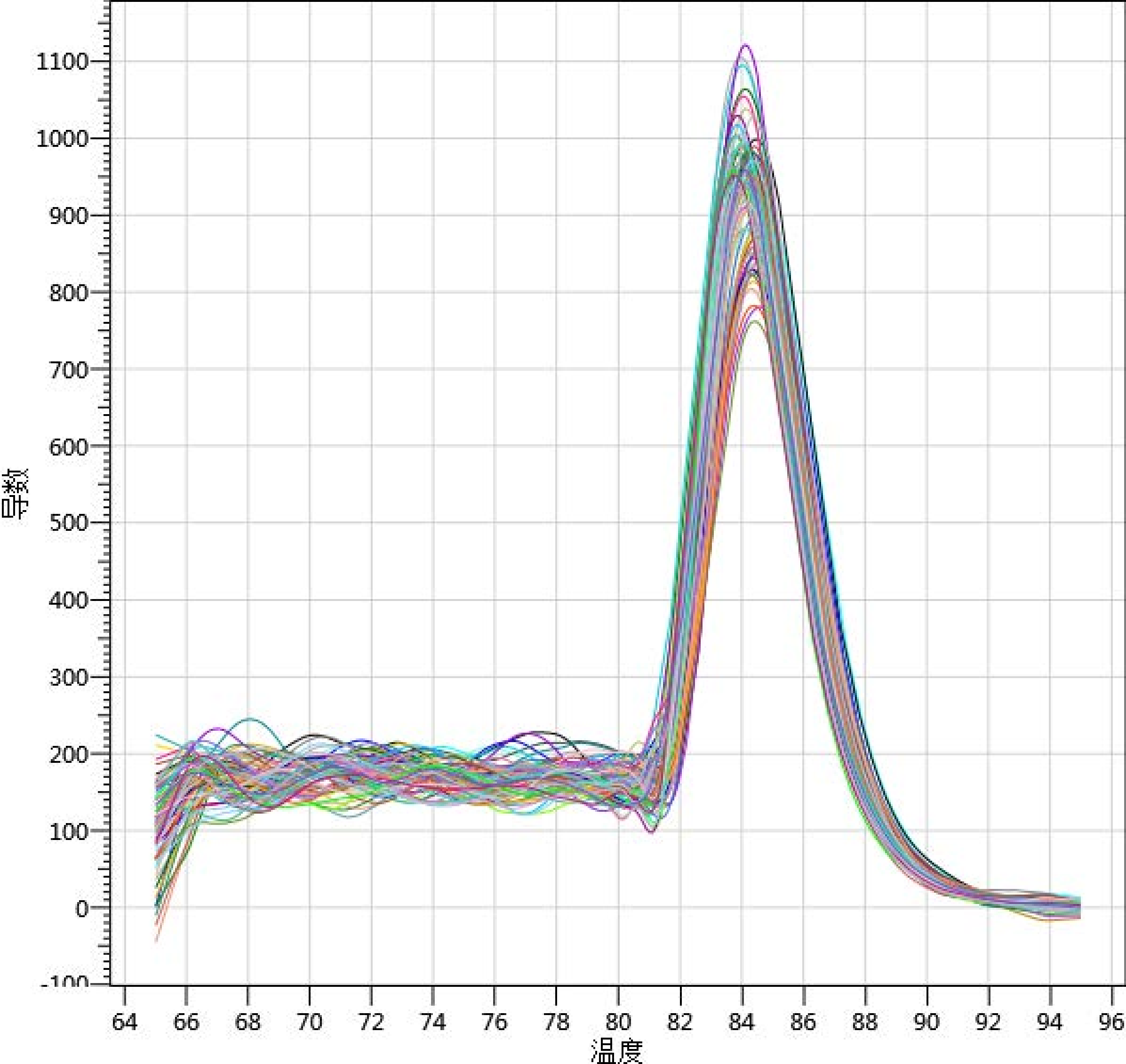

1100
1000
900
800
700
600
500
400
300
200
100
0
-100
导数
64
66
68
70
72
74
76
78
80
82
84
86
88
90
92
94
96
温度

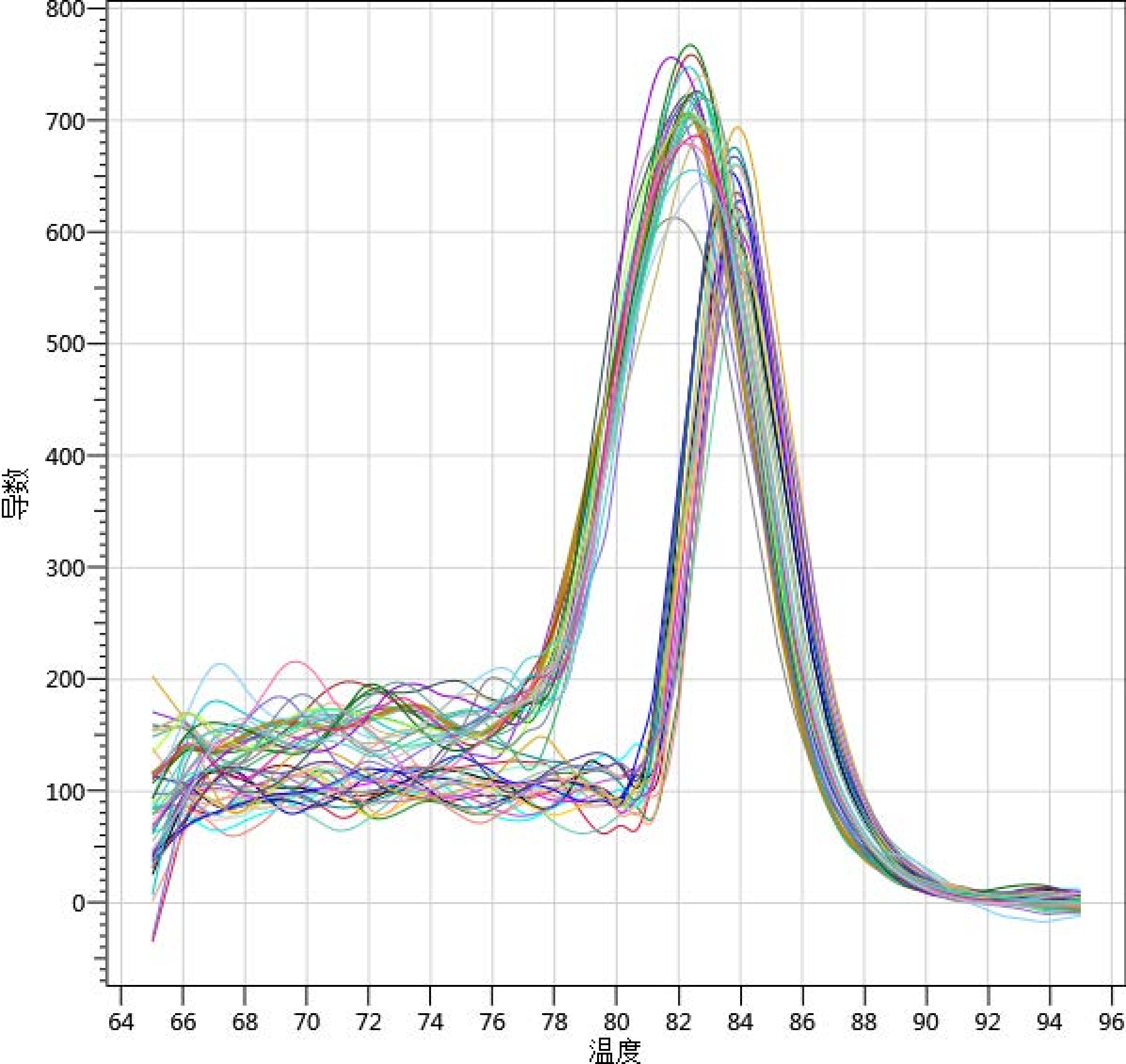

导数
温度
800
700
600
500
400
300
200
100
0
64
66
68
70
72
74
76
78
80
82
84
86
88
90
92
94
96

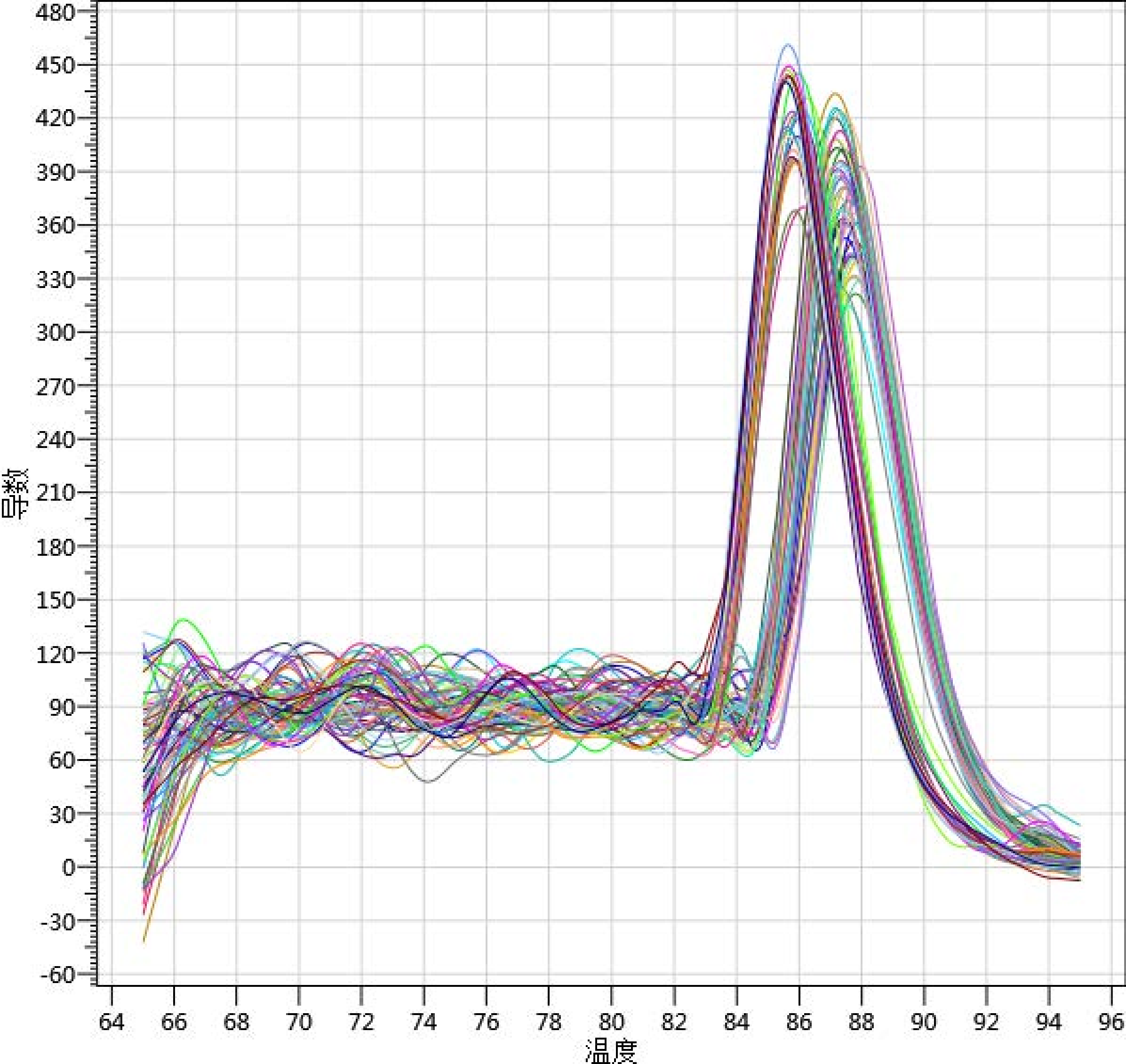

480
450
420
390
360
330
300
270
240
210
180
150
120
90
60
30
0
-30
-60
导数
64
66
68
70
72
74
76
78
80
82
84
86
88
90
92
94
96
温度

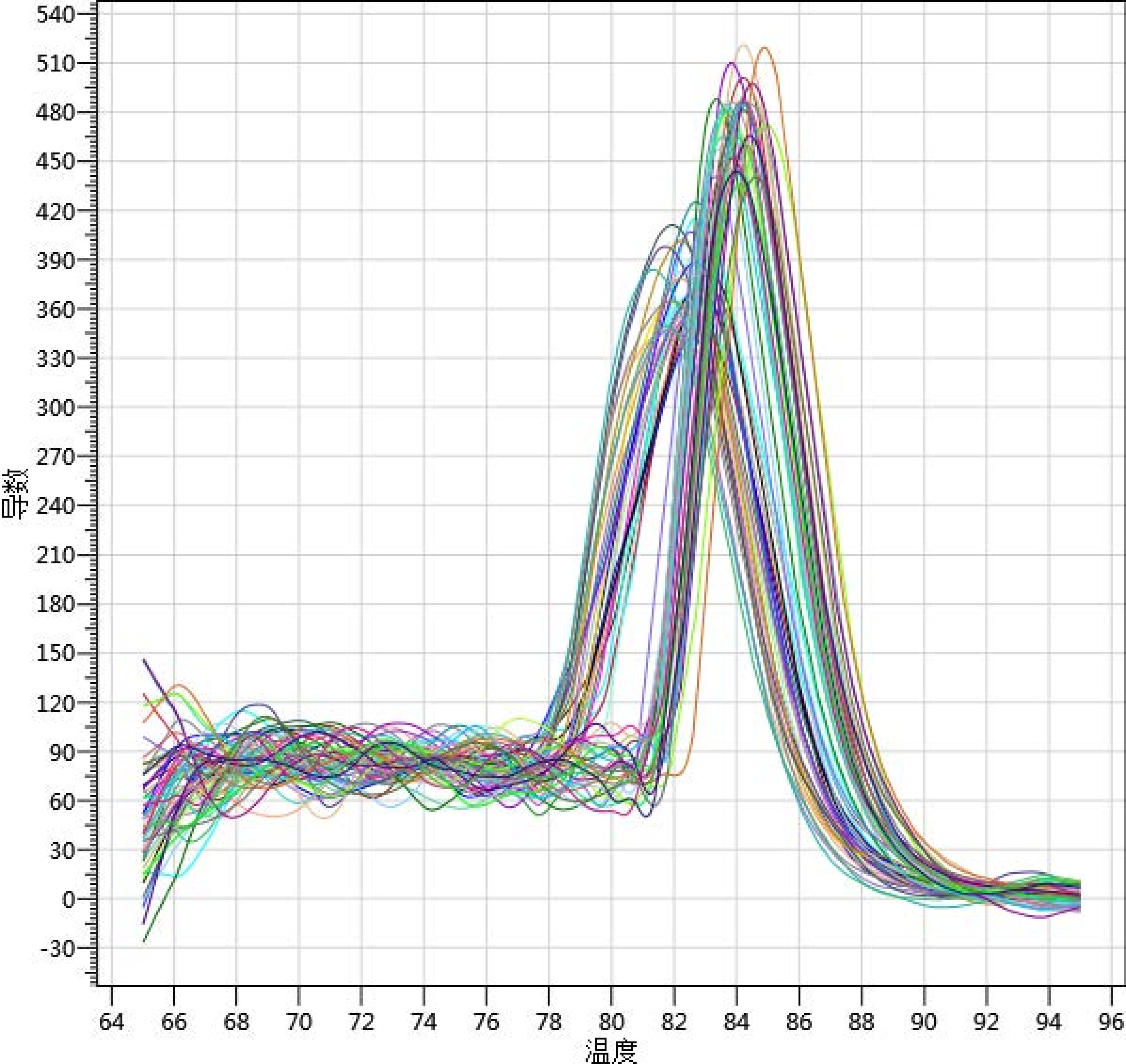

540
510
480
450
420
390
360
330
300
270
240
210
180
150
120
90
60
30
0
-30
导数
64
66
68
70
72
74
76
78
80
82
84
86
88
90
92
94
96
温度

In this study, goat spleen cDNA was used for *KLF*12 ORF clone, and the primers were designed according to the predicted sequence of goat *KLF*12 in GenBank (XM_005687692.3) Table 1. The RT-PCR reaction system shown in Table S1, amplification product shown in Fig. S1. 3'-Full RACE Core Set (TaKaRa, Otsu, Japan) kit was used to amplify the 3'UTR of *KLF*12 (Table S2, Fig. S2). Expression carriers were build using their plasmid respectively, the RT-PCR reaction systems and the pictures of amplification product shown in (Table S3-S5, Fig. S3-S5). Double digestion reaction system (Pmir-GLO-*KLF*12 WT/MT): *KLF*12 3'UTR agarose gel purified product and Pmir-GLO plasmid 1μg respectively, Q.cut XbaI and Q.cut XhoI 1μg respectively, 10×Q.cut Buffer 2 μL, supplement ddH2O to 20 μL, 37°C for 25 min. Double digestion reaction system (pcDNA3.1-OE *KLF*12): *KLF*12 ORF agarose gel purified product and pcDNA3.1 plasmid 1μg respectively, Q.cut XbaI and Q.cut KpnI 1μg respectively, 10×Q.cut Buffer 2 μL, supplement ddH2O to 20 μL, 37°C for 25 min. T4 ligase was used to connect the purified product of the target fragment with the purified product of Pmir-GLO/pcDNA3.1 vector, their concentration ratio was 4: 1 (16 °C for 3 h), then transform into DH5α competent cells.

Table S1 The RT-PCR reaction systems and procedure

RT-PCR system	Volume/μL	RT-PCR program		
cDNA	1	98°C	2min	
*KLF*12-F	1	98°C	10sec	35 Cycles
*KLF*12-R	1	58°C	30sec	
I-5^{TM}2xHigh-Fidelity	12.5	72°C	15sec	
Master Mix	9.5	72°C	5min	
ddH_2O		4°C	∞	

Table S2 The RT-PCR reaction systems and procedure

RT-PCR system-1	Volume/μL	RT-PCR system-2	Volume/μL	RT-PCR program		
cDNA	5	PCR-1 purified product	1	94°C	3min	
*KLF*12-outer	1	*KLF*12-inner	1	94°C	30sec	35 Cycles
Outer Primer	1	Inner Primer	1	60°C	30sec	
2×GC-Rich PCR	12.5	2×GC-Rich PCR	12.5	72°C	2min	
Master Mix	5.5	Master Mix	9.5	72°C	5min	
ddH_2O		ddH_2O		4°C	∞	

Table S3 The RT-PCR reaction systems and procedure

RT-PCR system	Volume/μL	RT-PCR program		
*KLF*12 3′ UTR plasmid	1	94°C	3min	
*KLF*12-WT-F	1	94°C	30sec	35 Cycles
*KLF*12-WT-R	1	64°C	30sec	
2×GC-Rich PCR	12.5	72°C	2min	
Master Mix	9.5	72°C	5min	
ddH_2O		4°C	∞	

Table S4 The RT-PCR reaction systems and procedure

RT-PCR system-1	Volume/μL	RT-PCR system-2	Volume/μL	RT-PCR program		
*KLF*12 3′ UTR plasmid	5	*KLF*12-MTF+MR	1	94°C	3min	
*KLF*12-MTF/MTR	1	*KLF*12-MF+MTR		94°C	30sec	
*KLF*12-MR/MF	1	*KLF*12-MTF/MTR	1	60°C	30sec	35 Cycles
2×GC-Rich PCR	12.5	2×GC-Rich PCR	1	72°C	2min	
Master Mix	5.5	Master Mix	12.5	72°C	5min	
ddH$_2$O		ddH$_2$O	9.5	4°C	∞	

Table S5 The RT-PCR reaction systems and procedure

RT-PCR system	Volume/μL	RT-PCR program		
*KLF*12 plasmid	1	94°C	3min	
*KLF*12-OE-F	1	94°C	30sec	
*KLF*12-OE-R	1	64°C	30sec	35 Cycles
2×GC-Rich PCR	12.5	72°C	2min	
Master Mix	9.5	72°C	5min	
ddH$_2$O		4°C	∞	

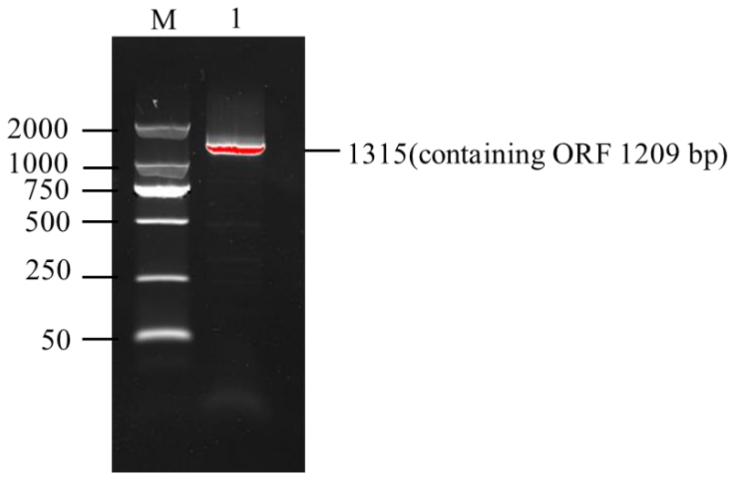

Fig.S1. Amplification products of goat *KLF*12

Not.: M: Marker D2000; 1: The amplified sequence of *KLF*12.

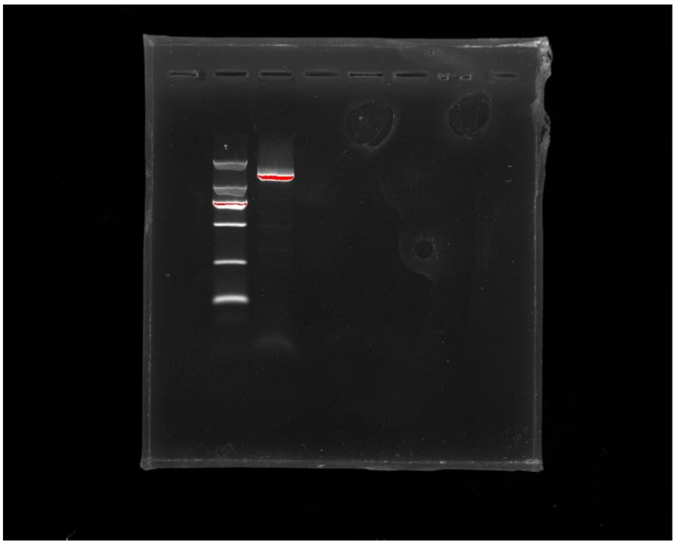

Fig.S1 Original picture

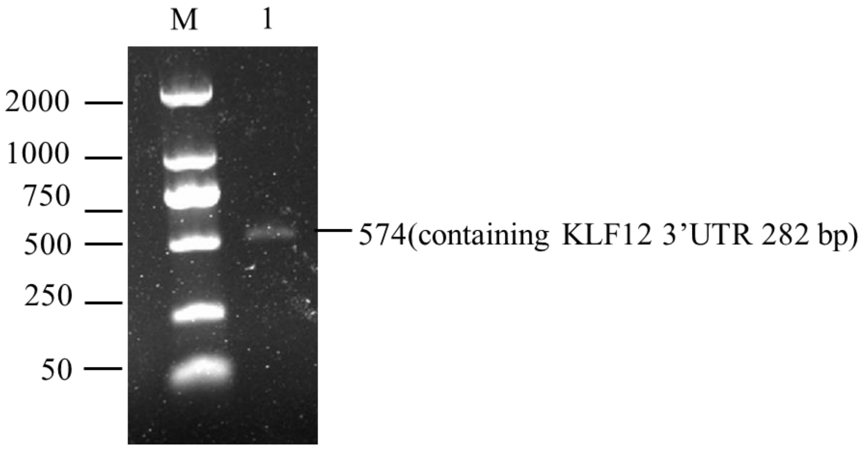

Fig. S2 Amplification products of goat KLF12 3′ UTR
M: Marker D2000; 1: The amplified sequence of KLF12 3′UTR.

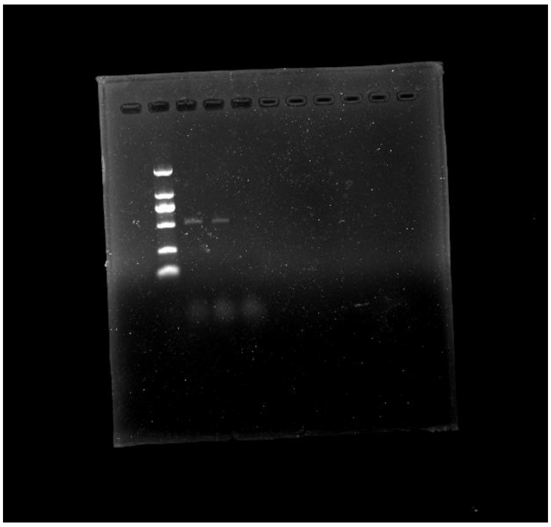

Fig. S2 Original picture

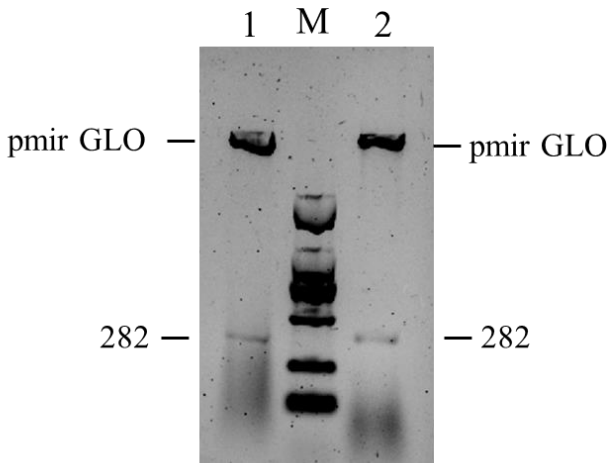

Fig. S3 The identification result of double enzyme digestion, 1: Pmir-GLO-KLF12 WT, M: Marker D2000, 2: Pmir-GLO-KLF12 MT

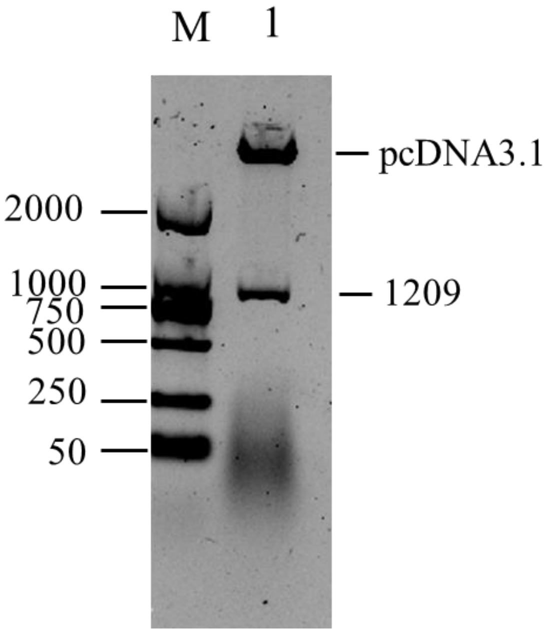

Fig. S4 The identification result of double enzyme digestion, 1: pcDNA3.1-KLF12 ; M: Marker D2000

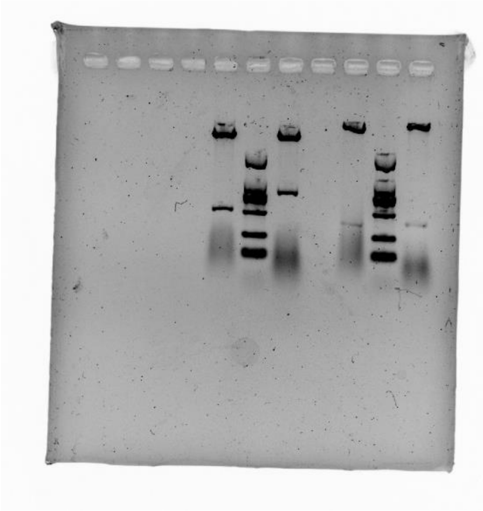

Fig. S5 Original picture of double enzyme digestion
